# Effects of gochujang formulations on menopausal symptoms and gut microbiota in postmenopausal women: an 8-week randomized, double-blind trial

**DOI:** 10.3389/fnut.2026.1849944

**Published:** 2026-09-10

**Authors:** A. Lum Han, Myeong Seon Ryu, Hee-Jong Yang, Do-Youn Jeong, Sae Ron Shin, Keum Ha Choi, Hyemi Lee

**Affiliations:** 1 Department of Family Medicine, Jeonbuk National University Hospital, Jeonju, Republic of Korea; 2 Microbial Institute for Fermentation Industry, Sunchang, Republic of Korea; 3 Department of Family Medicine, Wonkwang University Hospital, Iksan, Republic of Korea; 4 Department of Pathology, Wonkwang University Hospital, Iksan, Republic of Korea; 5 Department of Plastic and Reconstructive Surgery, Wonkwang University School of Medicine and Hospital, Iksan, Republic of Korea

**Keywords:** body composition, fermented food, gochujang, gut microbiota, Kupperman index, menopausal symptoms, probiotics

## Abstract

**Introduction:**

Menopause-associated symptoms impair quality of life, and postmenopausal women increasingly seek safe, non-hormonal, diet-based interventions. Gochujang, a traditional Korean fermented food rich in isoflavone aglycones and capsaicinoids, has biological activities relevant to menopausal health but has never been tested in a randomized trial. We compared three distinct Gochujang formulations.

**Methods:**

In this 8-week, randomized, double-blind, three-arm trial, two Gochujang formulations differing in microbial composition (high-microorganism [HTG], low-microorganism [LTG]) were compared with commercial Gochujang (CG) in 59 postmenopausal women with moderate-to-severe symptoms [Kupperman Menopausal Index (KMI) ≥15]. Change in total KMI score and change in gut microbiota were the two registered co-primary outcomes, and change in total KMI score is the primary endpoint of this report. Because all arms received active Gochujang without a placebo, the trial assessed between-formulation differences rather than efficacy. Secondary outcomes were body composition, metabolic biomarkers, and safety; gut microbiota and fecal short-chain fatty acids (SCFAs) are analyzed here as exploratory outcomes, a *post hoc* reclassification.

**Results:**

All formulations produced significant within-group KMI reductions (HTG: −3.65, 95% CI −4.97 to −2.33; LTG: −4.10, −5.98 to −2.22; CG: −3.42, −4.34 to −2.50; all *p* < 0.0001), with no statistically significant between-group differences, providing no evidence of formulation-specific effects. Percent body fat decreased and fecal SCFA concentrations declined in all groups. Gut microbiota composition shifted within all three groups, but the exploratory analyses did not provide robust evidence of formulation-specific differences. No adverse events occurred.

**Discussion:**

Eight-week Gochujang consumption was safe, well-tolerated, and associated with reduced menopausal symptom severity and percent body fat across all formulations. Efficacy cannot be established without a placebo control; placebo-controlled trials with endocrine and functional microbiome endpoints are warranted.

**Clinical trial registration:**

https://cris.nih.go.kr, identifier KCT0011085.

## 1 Introduction

Menopause represents a major physiological transition characterized by a decline in ovarian function and is frequently accompanied by a broad spectrum of symptoms, including vasomotor instability, sleep disturbance, fatigue, and mood alterations, all of which substantially impair the quality of life (1). Meanwhile, the global rise in obesity and metabolic syndrome has become a particular concern for postmenopausal women, who often experience unfavorable shifts in body fat distribution and increased metabolic risk following the decline in estrogen (2). Conventional management of moderate-to-severe menopausal symptoms primarily relies on hormone replacement therapy; however, long-term use is limited by safety concerns, including increased risks of cardiovascular events, venous thromboembolism, and hormone-dependent cancers (3). Consequently, many women actively seek safe, non-hormonal strategies that can relieve menopausal symptoms while also supporting metabolic health.

Traditional fermented foods have attracted increasing scientific interest due to their enhanced bioactivity resulting from microbial biotransformation during fermentation (4). Gochujang, a traditional Korean fermented red pepper paste, is a representative functional food composed of fermented soybean (Meju), glutinous rice, malt, and red pepper powder (5, 6). This fermentation process generates a complex matrix rich in bioactive compounds, including capsaicinoids from red pepper and isoflavone aglycones derived from fermented soybeans. These components are biologically relevant to both metabolic regulation and endocrine-related symptom modulation.

Preclinical studies have demonstrated that Gochujang supplementation suppresses weight gain, reduces lipid accumulation in adipose tissue and liver, and enhances energy expenditure in diet-induced obesity models (7). Human randomized controlled trials have also reported reductions in visceral fat area and improvements in blood lipid profiles in overweight individuals consuming Gochujang-containing products (7). Mechanistically, capsaicinoids activate transient receptor potential vanilloid 1 (TRPV1)-mediated thermogenesis and promote white adipose tissue browning, while isoflavone aglycones modulate adipogenic transcription factors such as SREBP-1c and PPARγ via AMPK-related pathways (8, 9). In parallel, Gochujang exhibits anti-inflammatory properties, including the suppression of pro-inflammatory cytokines and inhibition of nuclear factor kappa-light-chain-enhancer of activated B-cell signaling, as demonstrated in experimental colitis models (10). Capsaicin has also been shown to enhance intestinal barrier integrity by upregulating tight junction proteins, thereby reducing chronic low-grade inflammation (11). Additionally, Gochujang consumption has been associated with the modulation of gut microbiota composition in preclinical and dietary studies (12), and fermented foods more broadly may influence host physiology through microbial metabolites such as short-chain fatty acids (SCFAs), although inter-individual responses remain heterogeneous.

Beyond metabolic and gut-related effects, Gochujang may also exert cardiovascular benefits. Experimental studies have shown that it can attenuate high-salt-diet–induced increases in blood pressure through modulation of the renin–angiotensin–aldosterone system (13). The fermentation process further enhances the bioavailability of soybean isoflavones (daidzein, genistein, and glycitein), which have demonstrated vasorelaxant effects, partly mediated by increased nitric oxide production in vascular endothelial cells (14). Collectively, these findings indicate that Gochujang possesses a spectrum of biological activities relevant to metabolic regulation, inflammation control, vascular function, and endocrine-related symptom modulation, all of which are particularly relevant to the health concerns of postmenopausal women.

However, despite this biological plausibility, no randomized clinical trial has evaluated the effects of Gochujang in postmenopausal women. This represents a critical gap, as the postmenopausal state is characterized by unique hormonal, metabolic, and gut microbiome dynamics that may substantially influence the clinical response to fermented food interventions. Furthermore, Gochujang is commercially available in multiple formulations that differ markedly in microbial composition and preparation method; whether these differences translate into clinically meaningful differences in safety, tolerability, or health outcomes has never been examined in a controlled human trial.

Therefore, in this study, we aimed to conduct an 8-week, randomized, double-blind, three-arm comparative trial assessing the safety, tolerability, and effects on menopausal symptoms of three Gochujang formulations with differing microbial compositions: traditionally prepared high-beneficial-microorganism Gochujang (HTG), traditionally prepared low-beneficial-microorganism Gochujang (LTG), and commercially prepared Gochujang (CG). The primary endpoint of this report was the change in total Kupperman Menopausal Index (KMI) score, one of the two co-primary outcomes registered prospectively (Section 2.3). Secondary objectives included assessments of body composition, metabolic biomarkers, and health-related quality of life. Additionally, gut microbiota composition and fecal SCFA levels were assessed as exploratory biomarkers to examine potential biological correlates of formulation-specific effects. This study was designed as a formulation-comparative trial; accordingly, within-group changes should be interpreted in the context of shared Gochujang exposure rather than as evidence of causal efficacy.

## 2 Materials and methods

This was an 8-week, randomized, double-blind, three-arm comparative clinical trial comparing three Gochujang formulations (HTG, LTG, and CG). The trial was conducted at the Clinical Research Center of Wonkwang University Hospital (< city>Iksan < /city>, Republic of Korea), a Good Clinical Practice guideline-compliant facility fully equipped for specialized clinical trials. The interventions were delivered by board-certified physicians specialized in functional and family medicine with extensive experience in clinical research. Furthermore, the study was managed by clinical research coordinators board-certified in advanced nursing practice, ensuring a high standard of professional care, participant safety, and data integrity throughout the trial. Participants were required to visit the clinical center on three occasions, i.e., at screening/baseline (week 0), at an interim visit (week 4), and at the final assessment (week 8). The interim visit was conducted to monitor vital signs, assess adherence to the dosing regimen, and document any potential adverse events. The study was registered with the Korean Clinical Research Information Service (KCT0011085) and approved by the Institutional Review Board of Wonkwang University Hospital (WKUH IRB 2025-03-030-002), with all participants providing written informed consent.

The 8-week intervention period was selected based on prior clinical studies of dietary and phytoestrogen-related interventions in postmenopausal women, which demonstrated measurable changes in menopausal symptoms and metabolic parameters within similar timeframes. Practical considerations, including participant adherence, feasibility, and the exploratory nature of the formulation-comparison design, further supported the selection of this duration.

The primary efficacy endpoint of the present report was the change in total KMI score from baseline to week 8, one of the two co-primary outcomes registered prospectively (see “Outcome hierarchy and protocol history” below); the primary analysis compared the magnitude of change among HTG, LTG, and CG. The KMI is a validated rating scale for quantifying the severity of 11 common climacteric symptoms. Each symptom is rated on a 4-point severity scale (0 = none, 1 = mild, 2 = moderate, and 3 = severe), and the total score is calculated as a weighted sum. A higher score indicates greater symptom severity, and a decrease from baseline represents clinical improvement. In this study, a baseline KMI score of ≥15 was adopted as an inclusion criterion for defining moderate-to-severe menopausal syndrome.

Secondary endpoints included changes in body composition, including body weight, body mass index, percent body fat (PBF), and fat-free mass (FFM), measured via a bioelectrical impedance analysis. Standard biochemical assays, including lipid profile assays, were also conducted at baseline and week 8. A sample size of 60 participants (20 per group) was calculated to yield 80% power at a 5% significance level, accounting for a predicted dropout rate of 20%. Participants were recruited through advertisements on the Wonkwang University Hospital official website and institutional notice boards.

**Figure 1:**
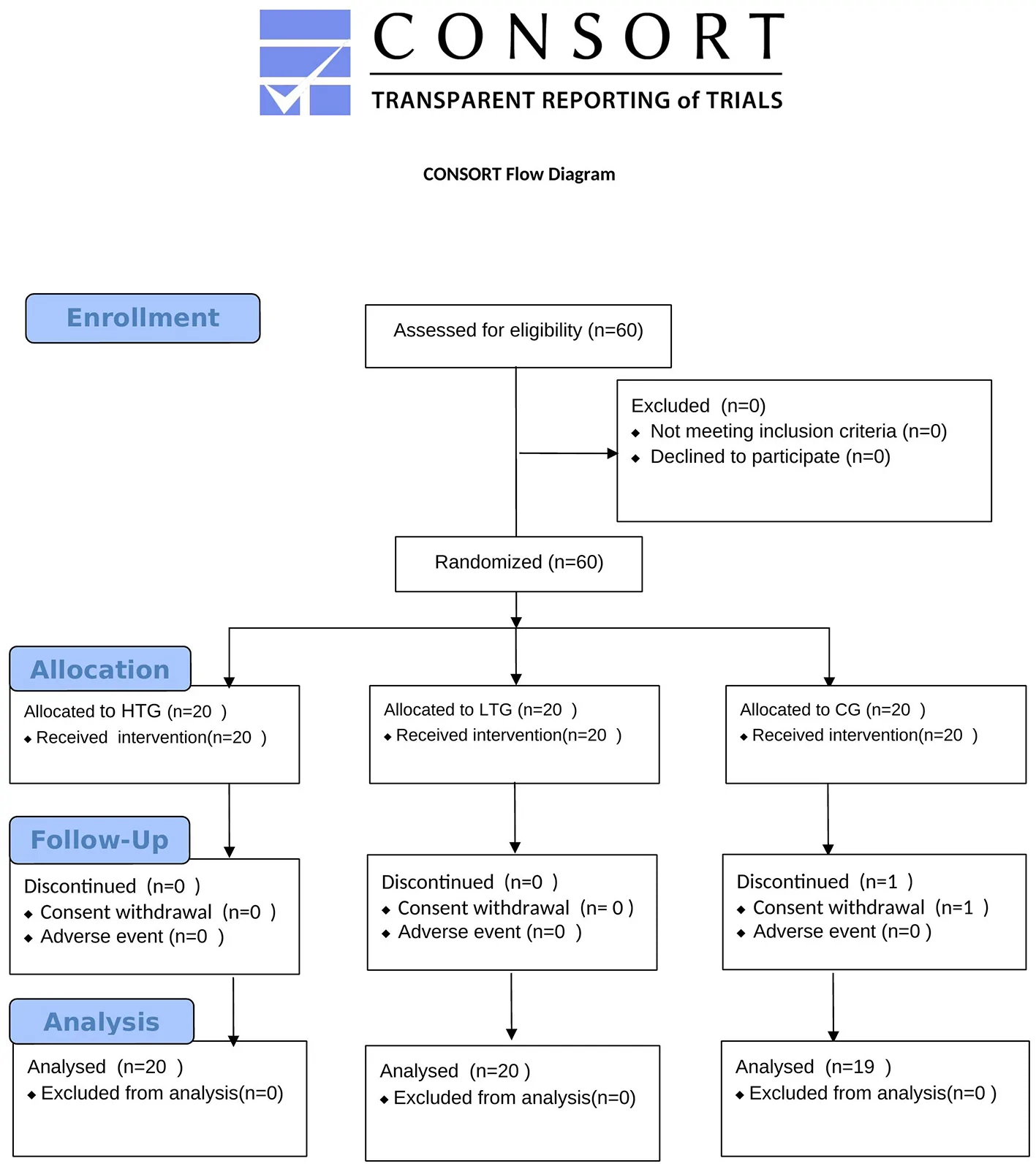
CONSORT Flow Diagram illustrating the enrollment, allocation, follow-up, and analysis of participants across the three Gochujang formulation groups [high-microorganism Gochujang (HTG), low-microorganism Gochujang (LTG), and commercially prepared Gochujang (CG)].

**Figure 2:**
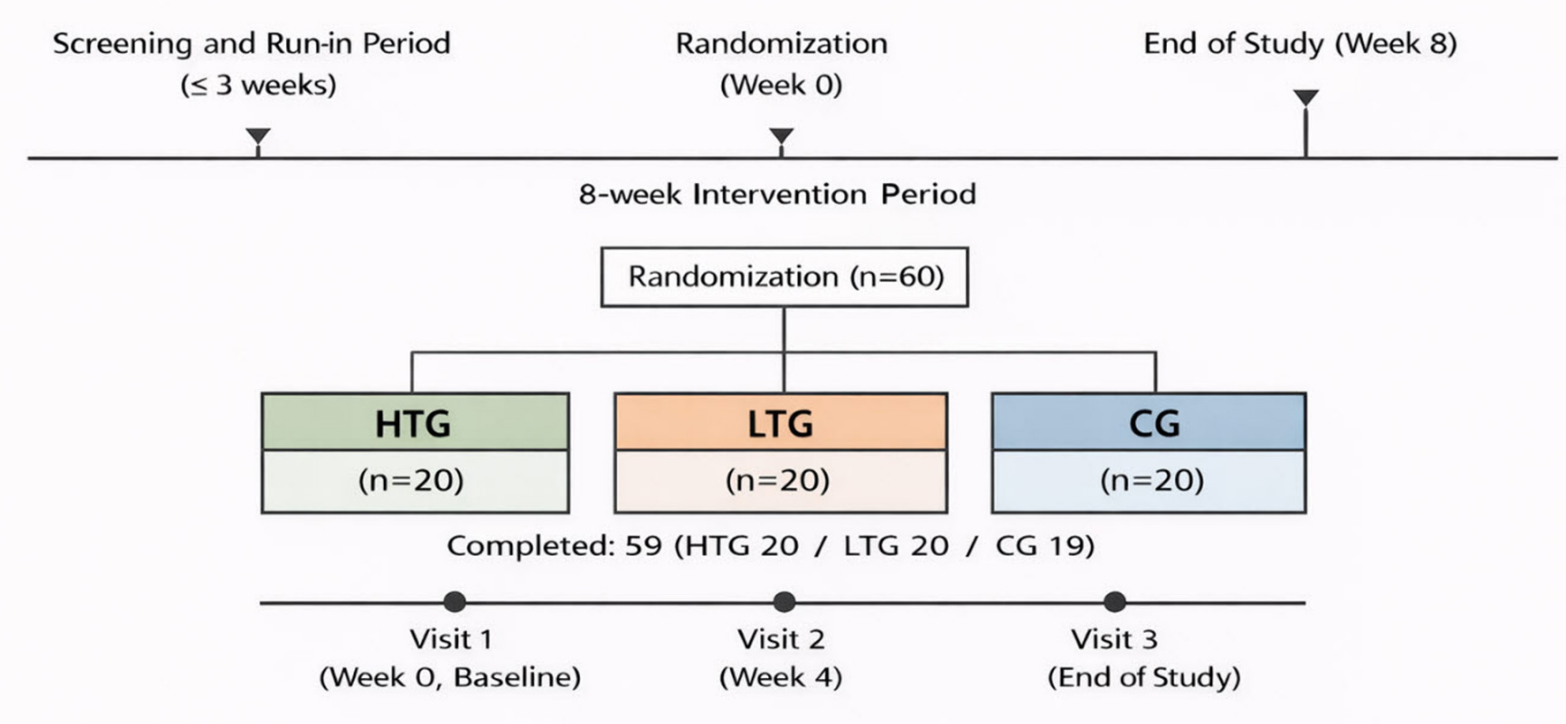
Schematic of the clinical trial design. HTG, Traditional Gochujang with high beneficial microorganism content; LTG, Traditional Gochujang with low beneficial microorganism content; CG, Commercial Gochujang.

Three distinct Gochujang formulations (HTG, LTG, and CG) were prepared as freeze-dried powder packaged in single-dose sachets for oral administration and analyses. Sixty volunteers were recruited and randomly allocated to one of the three treatment arms (n = 20 per group). Of the participants randomized, 59 completed the study protocol. The participant flow is illustrated in Figure 1 (CONSORT Flow Diagram), and the schematic of the clinical trial design is shown in Figure 2.

### 2.1 Study population and dosing protocol

Sixty female volunteers aged 45–70 years were enrolled in the trial. All participants were confirmed to be postmenopausal, defined either as experiencing 12 consecutive months of amenorrhea or through formal medical certification by an obstetrician-gynecologist. Furthermore, all participants were required to have menopausal syndrome of at least moderate severity, defined as a baseline KMI score of ≥15.

Exclusion criteria included: a body weight change of >10% within the previous 3 months; existing cardiovascular disease; known allergy to any component of the test material; chronic gastrointestinal disease; a history of major gastrointestinal surgery; participation in another clinical trial within the previous 2 months; impaired hepatic or renal function; use of antipsychotic medications within the previous 2 months; clinically significant abnormal laboratory results; psychological instability; history of substance abuse; or pregnancy or lactation. Additionally, individuals who had used systemic antibiotics within 4–8 weeks prior to enrollment were excluded due to the potential disruption of gut microbiota and the time required for microbiome recovery.

Table 1 illustrates the composition of the Gochujang products provided to the participants. The study products were prepared in powder form from freeze-dried Gochujang, as described, and packaged as single-dose sachets for oral administration. All three Gochujang formulations (HTG, LTG, and CG) were produced and supplied by the Microbial Institute for Fermentation Industry (MIFI; Sunchang, Republic of Korea) under GMP-compliant manufacturing conditions.

**Table 1:** Composition of the Gochujang powder formulations administered in sachet form.

Ingredient	HTG		LTG		CG	
	Content (g)	**Ratio (%)**	**Content (g)**	**Ratio (%)**	**Content (g)**	**Ratio (%)**
Freeze-dried Gochujang powder	19.0	75	19.0	75	19.0	75
Microcrystalline cellulose	5.1	20	5.1	20	5.1	20
Magnesium stearate	1.1	5	1.1	5	1.1	5
Total	25.2	100	25.2	100	25.2	100

HTG, Traditional Gochujang with high beneficial microbe content; LTG, Traditional Gochujang with low beneficial microbe content; CG, Commercially prepared Gochujang.

Participants were instructed to ingest three sachets twice daily, after their morning and evening meals. This regimen resulted in a total daily supplement intake of 25.2 g, corresponding to 19 g/day of dehydrated Gochujang powder.

This daily target dosage of Gochujang powder (19 g/day) was selected based on the 2014 Korean National Health and Nutrition Examination Survey, which identified the average daily red pepper paste intake and extreme consumption level for the Korean population to be 10.75 g and 36.86 g, respectively (15).

Based on previously reported compositional analyses, Gochujang typically contains approximately 220–250 kcal and 2,300–2,900 mg of sodium per 100 g. In the present study, the daily intake of 19 g/day of dehydrated Gochujang powder corresponds to an estimated energy intake of 40–50 kcal/day and sodium exposure of 440–550 mg/day. These values were derived from published literature and were intended to provide contextual interpretation rather than direct analytical measurements.

Beyond its macronutrient profile, Gochujang is a fermented product rich in bioactive compounds, including isoflavone aglycones and capsaicinoids. Fermentation substantially increases the proportion of isoflavone aglycones (approximately 75–86%), enhancing their bioavailability and potential estrogen-like biological activity (4–7).

Capsaicinoids derived from red pepper are known to promote thermogenesis and increase energy expenditure, while fermentation-derived metabolites may contribute to gut microbiome modulation and metabolic regulation.

An independent statistician used a permuted block design to implement restricted randomization. Allocation concealment was maintained through the use of sequentially numbered, opaque, sealed envelopes, which were inaccessible to enrollment personnel until assignment.

In this double-blind trial, participants, care providers, and outcome evaluators were kept unaware of group assignments. All study products were designed and manufactured to be indistinguishable in appearance, smell, taste, and packaging in order to maintain blinding. Unblinding was permitted only in the event of a medical emergency, although no such event occurred during the study.

Compliance with the intervention was assessed by counting returned sachets at each study visit. No participant was excluded due to non-adherence during the trial. Had adherence fallen below 70% of prescribed doses, the principal investigator would have assessed the case individually for potential discontinuation.

Participants were instructed not to change their usual diet, physical activity, or lifestyle habits during the 8-week intervention. This instruction was reinforced at every study visit through standardized counseling and follow-up questions. These procedures were implemented to minimize potential confounding effects and to ensure that observed outcomes were attributable primarily to the study intervention. Formal dietary assessment by registered dietitians was not undertaken in this trial; dietary and lifestyle counseling was delivered by clinical research coordinators through standardized instructions to maintain habitual eating patterns and physical activity.

### 2.2 Safety assessment

As a traditional Korean fermented food with centuries of continuous dietary use in the Korean population, Gochujang does not require the preclinical safety testing pathway mandated for novel pharmaceutical compounds or isolated chemical entities. Furthermore, prior animal studies have demonstrated that Gochujang consumption is safe, with no evidence of toxicity and documented anti-inflammatory, anti-hypertensive, and metabolic benefits in rodent models (7, 10, 12, 13). These nonclinical data, combined with the long history of human dietary exposure, provided a scientifically and ethically sound basis for conducting a human clinical trial under full Institutional Review Board oversight (WKUH IRB 2025-03-030-002) and trial registration (KCT0011085). Safety was assessed via physical examination and laboratory testing at each visit. Laboratory safety evaluations included a complete blood count and measurement of biochemical indicators of liver [alanine aminotransferase (ALT), aspartate aminotransferase (AST), total protein, and albumin] and kidney [blood urea nitrogen (BUN) and creatinine] function. Vital signs [pulse rate, systolic blood pressure (SBP) and diastolic blood pressure (DBP)] were recorded at every visit after a 10-min rest period.

### 2.3 Efficacy assessment

The primary efficacy endpoint was the change in KMI score, which reflects the severity of 11 climacteric symptoms (16). Secondary endpoints included metabolic, hepatic, and inflammatory biomarker levels, including the levels of complete lipid profile parameters (TC, LDL-C, HDL-C, triglyceride, non-HDL-C, and TG/HDL-C), hepatic enzyme (ALT, AST, and GGT) levels, renal indicator (BUN and creatinine) levels, glucose/insulin metabolism marker (fasting glucose and insulin) levels, and high-sensitivity C-reactive protein levels. Surrogate indices for insulin sensitivity (HOMA-IR, QUICKI) and β-cell function (HOMA-β) were also calculated. Health-related quality of life was additionally assessed using the EQ-5D index. The EQ-5D is a standardized instrument that evaluates five dimensions of health status (mobility, self-care, usual activities, pain/discomfort, and anxiety/depression). Higher EQ-5D index values indicate better perceived health status. Gut microbiota composition and fecal SCFAs were assessed as exploratory biomarkers to examine potential biological correlates of formulation differences.

Outcome hierarchy and protocol history. The trial was registered with the Clinical Research Information Service (KCT0011085), and two co-primary outcome measures were registered: change in gut microbiota, assessed at enrolment and at week 8, and change in the modified Kupperman index (reported here as the total KMI score), assessed at screening and at week 8. Body composition, body measurements, and lipid metabolism markers were registered as secondary outcomes. The protocol approved by the Institutional Review Board (WKUH IRB 2025-03-030-002) likewise listed gut microbiome–related parameters, including fecal SCFA concentrations, among the primary outcome measures. No amendment to the protocol or to the trial registration was made at any stage, and no amendment date therefore exists; the prespecified outcome hierarchy was never formally changed.

In the present report, change in total KMI score is presented as the primary endpoint, and gut microbiota composition and fecal SCFA concentrations are analyzed and interpreted as exploratory outcomes. This reclassification is *post hoc*: it was applied after completion of recruitment, database lock, and outcome analysis, and it is identified as such throughout. It was adopted because the sample size was determined on the KMI, whereas no prespecified analysis plan, target effect size, or strategy for controlling multiplicity had been defined for the microbiome outcomes. Because the reclassification was not prospective, both registered co-primary outcomes are reported in full and irrespective of statistical significance. The original IRB-approved protocol and the IRB and CRIS clarification documents are provided as Supplementary Files 1, 2.

### 2.4 Gut microbiota analysis

Fecal samples were collected at baseline and at the end of the 8-week intervention to assess changes in gut microbiota composition. Participants collected ≥1 g of fecal material using the MICROBE and ME Stool Collection Kit (Macrogen, Seoul, Republic of Korea). After collection, samples were temporarily stored in a household freezer by the participants prior to transport to the laboratory. Upon arrival at the laboratory, all samples were immediately transferred to and stored at −80 °C until analysis.

Microbial genomic DNA was extracted using a standardized extraction kit according to the manufacturer's instructions. DNA concentration was measured by NanoDrop One spectrophotometry (Thermo Fisher Scientific, Waltham, MA, USA). Purity was assessed by A260/A280 and A260/A230 ratios; samples with A260/A280 values outside the range of 1.8–2.0 or A260/A230 below 1.8 were excluded. DNA integrity was verified by 1% agarose gel electrophoresis prior to library preparation. The V3–V4 region of the bacterial 16S rRNA gene was amplified using the universal primers 341F and 805R and sequenced using the Illumina MiSeq platform (Illumina, San Diego, CA, USA).

Raw sequencing data were processed using the QIIME2 pipeline (version 2023.5), including quality filtering and denoising using DADA2, chimera removal, and taxonomic assignment based on the SILVA reference database (version 138). Samples with fewer than 10,000 reads were excluded, and the remaining samples were rarefied to an even sequencing depth prior to diversity analyses.

Microbial diversity was evaluated using the following alpha diversity indices: the Shannon diversity index, observed amplicon sequence variants, and Faith's phylogenetic diversity. Beta diversity metrics based on weighted and unweighted UniFrac distances. Beta diversity differences between groups and over time were assessed using permutational multivariate analysis of variance. For exploratory analysis, bacterial taxa were classified as ‘beneficial bacteria', including *Bifidobacterium, Lactobacillus, Faecalibacterium, Roseburia*, and *Akkermansia* spp.; ‘harmful bacteria', including *Escherichia-Shigella, Fusobacterium, Clostridium cluster I*, and *Desulfovibrio* spp.; and ‘others.' Classification was based on criteria reported in Marco et al. (17) and Koh et al. (18). This binary beneficial/harmful grouping is an operational simplification adopted for exploratory description; the ecological role of many taxa is context-dependent, and this classification is not intended to convey fixed functional attributes. All analyses were performed using standardized analytical procedures to ensure reproducibility.

### 2.5 Fecal SCFA analysis

SCFAs in fecal samples were analyzed using gas chromatography. Briefly, fecal samples were homogenized, acidified with 25% phosphoric acid, and extracted using diethyl ether as the organic solvent. 2-Ethylbutyric acid was used as an internal standard for quantification. The extracted samples were analyzed using a gas chromatography system (Agilent 7890B, Agilent Technologies, Santa Clara, CA, USA) equipped with a flame ionization detector and a capillary column (HP-FFAP, 30 m × 0.32 mm × 0.25 μm; Agilent Technologies). The oven temperature was programmed as follows: an initial temperature of 80 °C held for 1 min, increased at 10 °C/min to 180 °C and held for 5 min. Injector and detector temperatures were set at 220 °C and 250 °C, respectively.

Quantification of major SCFAs, including acetate, propionate, and butyrate, was performed using external standards (Sigma-Aldrich, St. Louis, MO, USA) prepared at five concentration levels (12.5, 25, 50, 100, and 200 mM). All calibration curves demonstrated good linearity (R^2^ > 0.99). The concentration of each SCFA was calculated from the calibration curves using the internal standard method and expressed as μmol/g wet feces.

### 2.6 Statistical analysis and data management

Statistical analyses were performed on an intention-to-treat basis using PASW Statistics version 23.0 (IBM Corporation, Armonk, NY, USA), with missing data handled using the last observation carried forward method, given the low dropout rate and short intervention duration. Continuous and categorical variables were reported as means ± standard deviation and frequencies (%), respectively. To assess baseline homogeneity, cross-tabulation (Chi-square test) was used for categorical variables (e.g., alcohol consumption and smoking status), while one-way analysis of variance (ANOVA) was used for continuous variables.

Normality of continuous variables was assessed using the Shapiro–Wilk test. Homogeneity of variance was evaluated using Levene's test prior to one-way ANOVA. Parametric tests were applied when both assumptions were satisfied; otherwise, nonparametric alternatives were used. To evaluate changes over time within each group, within-group comparisons relative to baseline (week 0) to week 8 were performed using paired sample *t*-tests (or Wilcoxon signed-rank tests, as appropriate). Between-group comparisons were conducted using change-from-baseline values (Δ = week 8 – baseline). Because the objective was to compare changes among three randomized formulations with balanced baseline characteristics, change-from-baseline analyses were prespecified. Depending on data distribution, one-way ANOVA was used for comparisons among the three groups when assumptions were met; otherwise, nonparametric tests were applied as appropriate (e.g., Kruskal–Wallis for overall comparisons and/or pairwise Mann–Whitney U tests). All tests were two-sided, and *p* < 0.05 was considered statistically significant.

To enhance the robustness of the analyses, sensitivity analyses were performed using both parametric and nonparametric methods, as appropriate. The direction and magnitude of the observed changes remained consistent across analytical methods. In addition to p-values, standardized effect size estimates were calculated and reported: Cohen's d was used for between-group comparisons of continuous variables, and eta-squared (η^2^) was calculated for one-way ANOVA analyses. Effect sizes were interpreted as small (d = 0.2; η^2^ = 0.01), medium (d = 0.5; η^2^ = 0.06), or large (d = 0.8; η^2^ = 0.14). All statistical analyses and their appropriateness for this study design were independently reviewed and confirmed by a biostatistician.

## 3 Results

### 3.1 Changes in menopausal symptoms as assessed using the Kupperman index

All three formulation groups (HTG, LTG, and CG) were associated with reductions in total KMI scores over the 8-week period [HTG: −3.65, 95% confidence interval (CI) −4.97, −2.33, *p* < 0.0001; LTG: −4.10, 95% CI −5.98, −2.22, *p* < 0.0001; CG: −3.42, 95% CI −4.34, −2.50, *p* < 0.0001] (Table 2). For individual KMI components, all groups exhibited reductions in key symptoms. For example, vasomotor symptoms decreased in HTG (−2.25, *p* < 0.0001), LTG (−2.00, *p* = 0.002), and CG (−1.68, *p* = 0.002) (Table 2). Paresthesia also declined in all groups (HTG: −0.50, *p* = 0.021; LTG: −0.80, *p* = 0.008; CG: −0.74, *p* = 0.015) (Table 2). EQ-5D index scores showed no statistically significant change in any group (HTG: +0.01, *p* = 0.163; LTG: +0.004, *p* = 0.330; CG: +0.011, *p* = 0.171) (Table 2).

**Table 2:** Changes in menopausal symptoms and health-related quality of life following the intervention.

Value	Group								
	HTG (***n*** = **20)**			**LTG (*****n*** = **20)**			**CG (*****n*** = **19)**		
	**Before**	**After**	***p*** **-value**	**Before**	**After**	***p*** **-value**	**Before**	**After**	***p*** **-value**
Vasomotor symptoms	9 ± 2.55	7 ± 2.2	< 0.0001	8.8 ± 2.78	6.8 ± 2.28	0.002	8.84 ± 2.85	7.16 ± 2.85	0.002
Paresthesia	1.5 ± 1.43	1 ± 1.03	0.021	2 ± 1.3	1.2 ± 1.2	0.008	1.79 ± 1.75	1.05 ± 1.22	0.015
Insomnia	3.7 ± 0.98	3.6 ± 1.39	0.789	3.8 ± 1.94	3.7 ± 1.17	0.853	3.37 ± 1.5	3.47 ± 1.47	0.716
Nervousness	1.1 ± 1.37	0.5 ± 0.89	0.030	0.9 ± 1.37	0.3 ± 0.73	0.055	0.84 ± 1.38	0.32 ± 0.75	0.021
Melancholia	0 ± 0	0 ± 0	–	0.05 ± 0.22	0 ± 0	0.330	0.16 ± 0.5	0 ± 0	0.187
Vertigo	0.95 ± 0.69	0.55 ± 0.83	0.042	0.75 ± 0.64	0.5 ± 0.61	0.056	1 ± 0.82	0.53 ± 0.61	0.001
Fatigue	2.4 ± 0.75	2.45 ± 0.76	0.834	2.3 ± 0.66	2.5 ± 0.61	0.330	2.21 ± 0.71	2.58 ± 0.61	0.049
Headache	0.6 ± 0.75	0.4 ± 0.6	0.258	0.65 ± 0.59	0.35 ± 0.49	0.030	0.89 ± 0.74	0.53 ± 0.51	0.031
Arthralgia and myalgia	1.8 ± 0.95	2.1 ± 0.85	0.267	1.6 ± 0.94	1.6 ± 0.82	1.000	1.84 ± 0.69	1.95 ± 0.62	0.650
Palpitation	0.35 ± 0.88	0.05 ± 0.22	0.110	0.15 ± 0.67	0.05 ± 0.22	0.541	0 ± 0	0 ± 0	–
Formication	0.05 ± 0.22	0.0 ± 0.0	0.330	0.1 ± 0.45	0.0 ± 0.0	0.330	0.05 ± 0.23	0.0 ± 0.0	0.331
Total	21.3 ± 2.39	17.65 ± 2.25	< 0.0001	21.1 ± 3.67	17 ± 3.29	< 0.0001	21 ± 2.87	17.58 ± 2.69	< 0.0001
Vaginal dryness	1.3 ± 0.57	1 ± 0.46	0.010	1.45 ± 0.83	1.15 ± 0.59	0.030	1.21 ± 0.71	1.11 ± 0.46	0.429
EQ-5D	0.99 ± 0.03	1.00 ± 0	0.163	0.996 ± 0.02	1.00 ± 0	0.330	0.989 ± 0.03	1.00 ± 0	0.171

Values are expressed as the mean ± standard deviation. The statistical significance of within-group changes from before to after the intervention was determined using a paired *t*-test. *P*-values < 0.05 were considered statistically significant. HTG, Traditional Gochujang with high beneficial microbe content; LTG, Traditional Gochujang with low beneficial microbe content; CG, Commercially prepared Gochujang.

**Figure 3:**
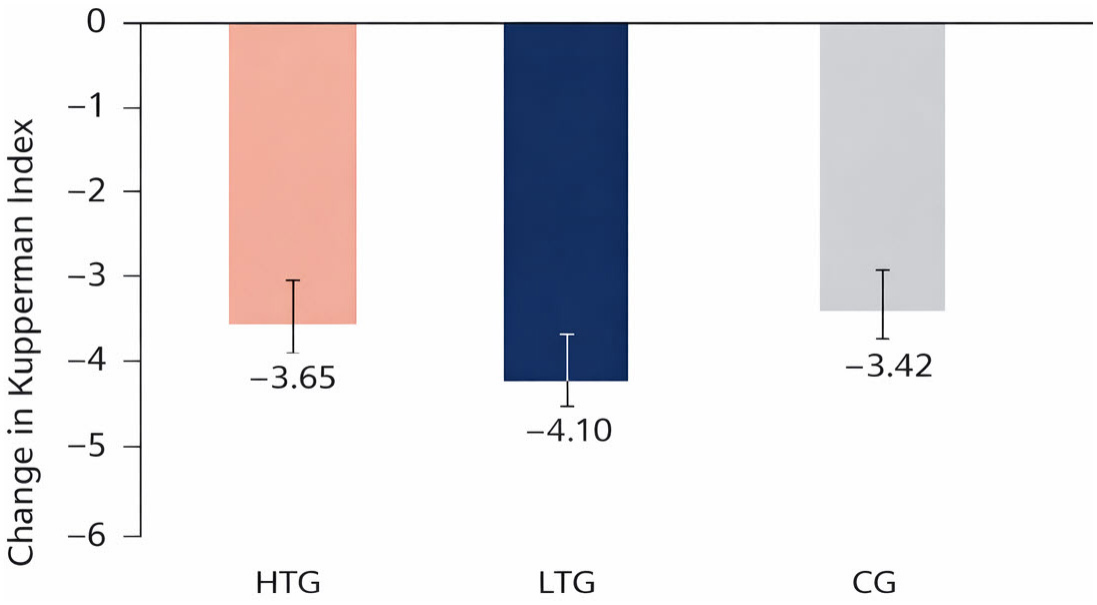
Changes in menopausal symptoms following the intervention.

Between-group comparisons of changes from baseline revealed that the magnitude of total KMI reduction did not differ significantly among HTG, LTG, and CG (Table 3). Figure 3 illustrates that total KMI score improvements were comparable across the three groups, with no statistically significant differences in change from baseline.

**Table 3:** Between-group comparison of changes in key endpoints (Δ = week 8 – baseline).

Endpoint	HTG (mean ±SD)	LTG (mean ±SD)	CG (mean ±SD)	HTG vs. LTG (p)	LTG vs. CG (p)	HTG vs. CG (*p*)
ΔKMI	−3.65 ± 2.82 [95% CI −4.97, −2.33]	−4.10 ± 4.02 [95% CI −5.98, −2.22]	−3.42 ± 1.90 [95% CI −4.34, −2.50]	0.564	0.341	0.676
ΔPBF (%)	−0.63 ± 0.95	−1.47 ± 2.96	−0.67 ± 1.34	0.094	0.129	0.899
ΔBFM (kg)	−0.59 ± 0.71	−2.26 ± 8.00	−0.50 ± 1.24	0.196	0.177	0.696
ΔTotal SCFA	−176.64 ± 190.85	−201.31 ± 168.99	−152.08 ± 130.19	0.376^*^	0.298^*^	0.810^*^
ΔBeneficial bacteria (%)	−5.12 ± 10.09	−10.14 ± 11.92	−5.76 ± 12.12	0.046	0.112	0.800

Values are expressed as mean ± standard deviation (SD). Δ indicates change from baseline to week 8. For the primary outcome (ΔKMI), values in brackets are 95% confidence intervals for the within-group change. Between-group comparisons were performed using independent *t*-tests for normally distributed variables. Variables marked with ^*^were analyzed using Mann–Whitney U tests due to non-normal distribution. No adjustment for multiple comparisons was applied. Statistical significance was defined as *p* < 0.05. HTG, Traditional Gochujang with high beneficial microbe content; LTG, Traditional Gochujang with low beneficial microbe content; CG, Commercially prepared Gochujang. ^aAnalyzed using the Mann–Whitney U test due to non-normal distribution.

### 3.2 Changes in anthropometric and body composition parameters

**Figure 4:**
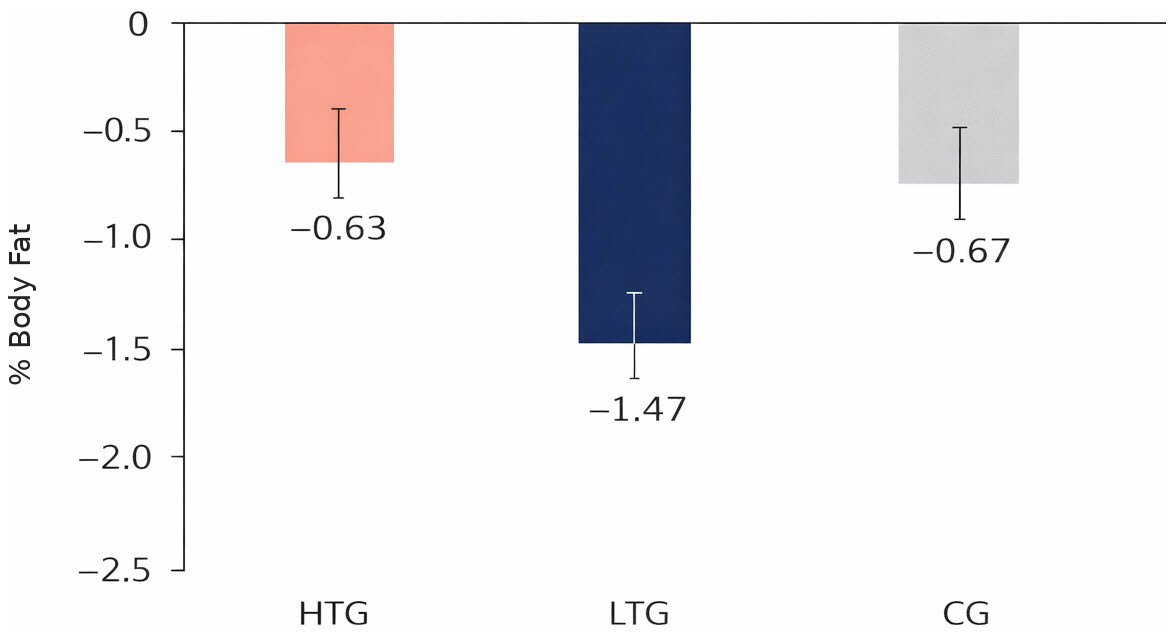
Changes in percentage body fat across the three Gochujang intervention groups. HTG, Traditional Gochujang with high beneficial microbe content; LTG, Traditional Gochujang with low beneficial microbe content; CG, Commercially prepared Gochujang.

PBF decreased in HTG (−0.63%, *p* = 0.008), LTG (−1.47%, *p* = 0.041), and CG (−0.67%, *p* = 0.045) (Table 4 and Figure 4). HTG also showed a significant reduction in body fat mass (BFM: −0.59 kg, *p* = 0.002). In LTG, FFM increased (+0.97 kg, *p* = 0.025), with a corresponding rise in basal metabolic rate (+21.00 kcal, *p* = 0.024). Between-group comparisons of ΔPBF and ΔBFM again showed no significant differences among the groups (Table 3).

**Table 4:** Changes in anthropometric and body composition parameters across the three Gochujang groups.

Value	Group								
	HTG (***n*** = **20)**			**LTG (*****n*** = **20)**			**CG (*****n** =* **19)**		
	**Before**	**After**	***p*** **-value**	**Before**	**After**	***p*** **-value**	**Before**	**After**	***p*** **-value**
WC (cm)	87.18 ± 3.87	87.1 ± 3.92	0.288	88.02 ± 6.24	87.96 ± 6.26	0.713	88.98 ± 6.6	88.93 ± 6.58	0.495
AFR (%)	0.91 ± 0.03	0.9 ± 0.03	0.065	0.92 ± 0.04	0.91 ± 0.05	0.131	0.92 ± 0.04	0.92 ± 0.03	0.772
WHR	0.91 ± 0.04	0.91 ± 0.04	0.577	0.90 ± 0.04	0.9 ± 0.04	0.481	0.92 ± 0.05	0.92 ± 0.05	0.331
Weight (kg)	63.01 ± 5.98	62.52 ± 6.03	0.055	64.83 ± 8.13	65.04 ± 8.69	0.477	64.85 ± 5.75	64.71 ± 5.45	0.648
BMI (kg/m^2^)	26.05 ± 2.05	25.87 ± 2.32	0.069	26.2 ± 2.56	26.29 ± 2.81	0.465	26.94 ± 2.73	26.9 ± 2.79	0.781
BFM (kg)	23.71 ± 3.74	23.12 ± 3.71	0.002	26.42 ± 7.04	24.16 ± 4.82	0.228	25.07 ± 4.48	24.57 ± 4.16	0.099
PBF (%)	37.48 ± 3.63	36.85 ± 3.59	0.008	38.37 ± 2.5	36.90 ± 3.43	0.041	38.51 ± 4.97	37.84 ± 4.68	0.045
FFM (kg)	39.3 ± 3.55	39.4 ± 3.63	0.620	39.91 ± 4.71	40.88 ± 4.57	0.025	39.77 ± 3.59	40.13 ± 3.57	0.064
BMR (kcal)	1,219.1 ± 76.58	1,221.1 ± 78.37	0.648	1,231.85 ± 101.58	1,252.85 ± 98.48	0.024	1,228.68 ± 77.39	1,236.79 ± 76.97	0.772

AFR, Abdominal Fat Rate; BFM, Body Fat Mass; BMI, Body Mass Index; BMR, Basal Metabolic Rate; FFM, Fat-Free Mass; PBF, Percent Body Fat; WC, Waist Circumference; WHR, Waist-to-Hip Ratio; HTG, Traditional Gochujang with high beneficial microbe content; LTG, Traditional Gochujang with low beneficial microbe content; CG, Commercially prepared Gochujang. Values are expressed as mean ± standard deviation (SD). The statistical significance of within-group changes from before to after the intervention was determined using a paired *t*-test. *P-*values < 0.05 were considered statistically significant.

### 3.3 Changes in systemic inflammatory and metabolic biomarkers

No statistically significant changes from baseline were observed in inflammatory markers or glucose metabolism indices (fasting glucose, insulin, HOMA-IR, HOMA-β) in any group (Table 5). Lipid parameters (total cholesterol, LDL-C, and others) remained largely unchanged. Although HDL-C decreased slightly in one group, the absolute change was small and remained within the normal range, rendering it clinically insignificant.

**Table 5:** Changes in systemic inflammatory and metabolic biomarkers after 8 weeks of Gochujang supplementation.

Value	Group								
	HTG (***n*** = **20)**			**LTG (*****n*** = **20)**			**CG (*****n*** = **19)**		
	**Before**	**After**	***p*** **-value**	**Before**	**After**	***p*** **-value**	**Before**	**After**	***p*** **-value**
CRP (mg/L)	0.85 ± 0.7	1.24 ± 1.61	0.163	0.81 ± 0.73	1.85 ± 2.95	0.101	1.47 ± 2.15	1.64 ± 2.03	0.740
ESR (mm/h)	6.65 ± 4.98	7.7 ± 5.94	0.230	6.95 ± 3.93	7.9 ± 5.37	0.510	8.32 ± 6.83	8.63 ± 5.95	0.795
HDL (mg/dL)	57.15 ± 14.69	54.9 ± 11.83	0.131	60.8 ± 11.98	57.4 ± 11.3	0.029	50.74 ± 16.32	53.26 ± 12.68	0.274
LDL (mg/dL)	108.7 ± 45.86	105.75 ± 43.03	0.544	101 ± 31.6	99.4 ± 26.52	0.713	117.74 ± 40.3	110.42 ± 38.62	0.336
TC (mg/dL)	194.35 ± 51.1	184.7 ± 47.61	0.202	187.1 ± 35.43	184.9 ± 27.31	0.630	195.53 ± 36.63	188 ± 39.61	0.327
Triglyceride	157.7 ± 192.01	126.1 ± 52.26	0.395	115.45 ± 74.93	140.0 ± 122.6	0.167	110.84 ± 47.58	112.16 ± 51.12	0.930
Non-HD	137.2 ± 50.16	129.8 ± 45.76	0.350	126.3 ± 37.79	127.5 ± 32.75	0.790	144.79 ± 39.13	134.74 ± 40.21	0.220
TG/HDL	3.56 ± 6.45	2.45 ± 1.29	0.399	2.08 ± 1.67	2.86 ± 3.63	0.162	2.28 ± 1.18	2.14 ± 1.31	0.628
Glucose (mg/dL)	98.2 ± 9.04	101.35 ± 8.89	0.062	106.6 ± 18	107.3 ± 14.28	0.776	104.47 ± 15.69	104 ± 12.41	0.862
Insulin (μU/mL)	6.75 ± 3.88	7.48 ± 3.65	0.298	7.51 ± 4.17	8.84 ± 4.62	0.112	6.43 ± 3.11	8.28 ± 5.62	0.097
HOMA-IR	1.65 ± 1	1.91 ± 1.05	0.198	2.07 ± 1.4	2.40 ± 1.38	0.182	1.7 ± 0.92	2.14 ± 1.41	0.140
HOMA-β	72.69 ± 49.17	70.58 ± 30.24	0.795	62.92 ± 27.96	73.83 ± 33.43	0.081	57.38 ± 27.19	76.17 ± 59.96	0.071
QUICKI	0.38 ± 0.07	0.36 ± 0.03	0.095	0.36 ± 0.04	0.33 ± 0.05	0.059	0.36 ± 0.04	0.35 ± 0.04	0.088

CRP, C-Reactive Protein; ESR, Erythrocyte Sedimentation Rate; HDL, High-Density Lipoprotein; HOMA-β, Homeostatic Model Assessment for β-cell function; HOMA-IR, Homeostatic Model Assessment for Insulin Resistance; LDL, Low-Density Lipoprotein; QUICKI, Quantitative Insulin Sensitivity Check Index; TC, Total Cholesterol; HTG, Traditional Gochujang with high beneficial microbe content; LTG, Traditional Gochujang with low beneficial microbe content; CG, Commercially prepared Gochujang. Values are expressed as mean ± standard deviation (SD). The statistical significance of within-group changes from before to after the intervention was determined using a paired *t-*test. *P*-values < 0.05 were considered statistically significant.

### 3.4 Exploratory changes in gut microbiota composition

**Figure 5:**
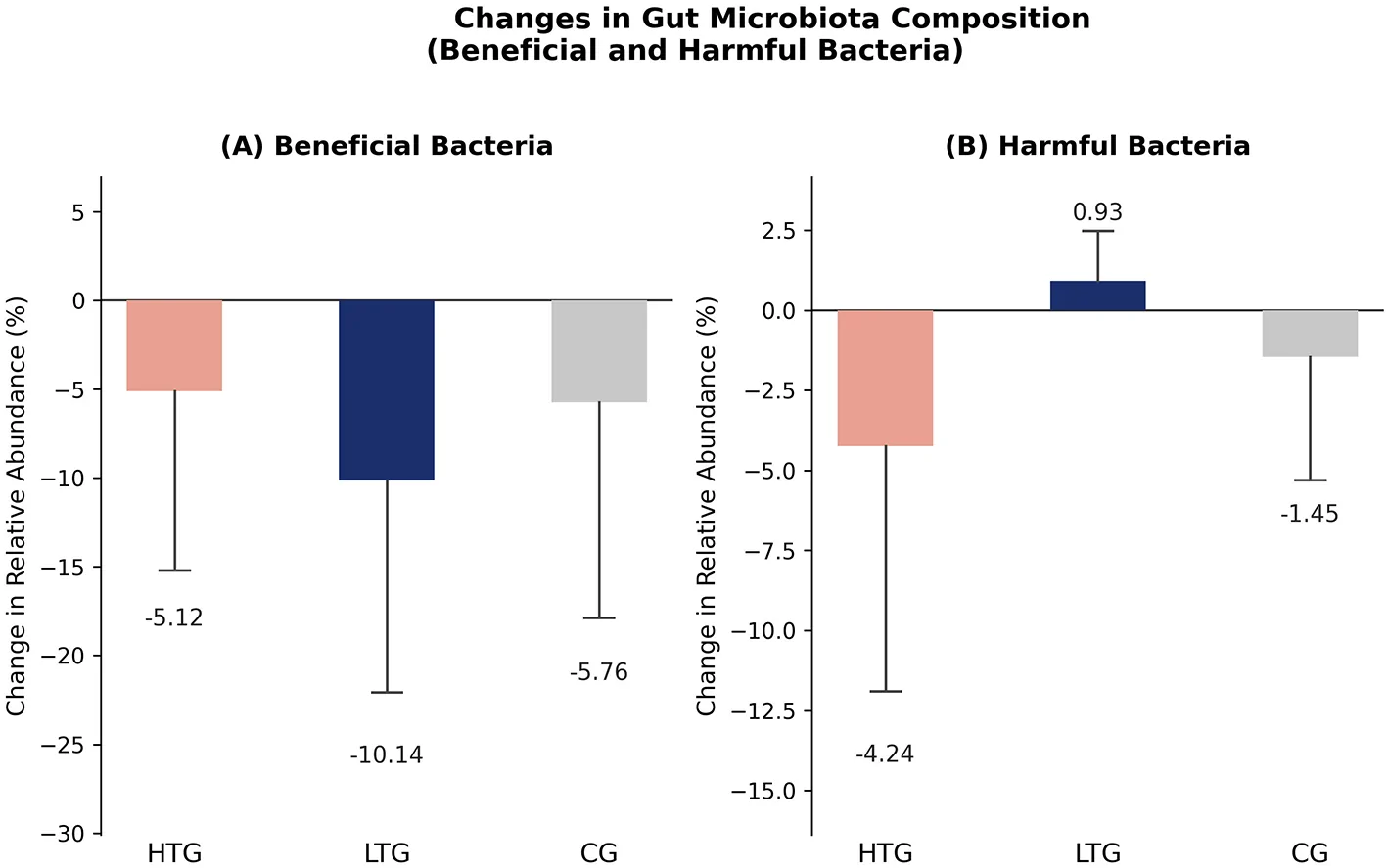
Changes in gut microbiota composition following the 8-week intervention. **(A)** Beneficial bacteria; **(B)** Harmful bacteria. Data are presented as mean change from baseline (Δ). HTG, Traditional Gochujang with high beneficial microorganism content; LTG, Traditional Gochujang with low beneficial microorganism content; CG, Commercial Gochujang.

All groups showed shifts in the proportions of “beneficial” and “harmful” bacteria; the within-group reduction in harmful bacteria was statistically significant in HTG but not in LTG or CG (Table 6 and Figure 5).

**Table 6:** Exploratory changes in fecal gut microbiota composition.

Value	Group								
	HTG (***n*** = **20)**			**LTG (*****n** =* **20)**			**CG (*****n*** = **19)**		
	**Before**	**After**	***p*** **-value**	**Before**	**After**	***p*** **-value**	**Before**	**After**	***p*** **-value**
Beneficial bacteria (%)	33.16 ± 11.07	28.04 ± 6.48	0.037	35.22 ± 11.85	25.08 ± 11.21	0.001	33.77 ± 8.15	28.01 ± 10.22	0.056
Harmful bacteria (%)	6.21 ± 7.65	1.97 ± 1.15	0.013	1.96 ± 1.56	2.89 ± 2.96	0.227	4.18 ± 3.84	2.73 ± 2.31	0.152
Others (%)	60.63 ± 11.31	69.99 ± 6.38	0.003	62.82 ± 12.19	72.03 ± 11.47	0.003	62.05 ± 8.26	69.27 ± 10.34	0.014

Values are expressed as the mean ± standard deviation (SD). The statistical significance of within-group changes from before to after the intervention was determined using a paired *t*-test. Microbial categories (“beneficial,” “harmful,” and “others”) were grouped according to functional classification criteria applied in this study. These analyses were conducted as exploratory assessments to describe potential shifts in gut microbial composition. HTG, Traditional Gochujang with high beneficial microbe content; LTG, Traditional Gochujang with low beneficial microbe content; CG, Commercially prepared Gochujang.

### 3.5 Exploratory changes in fecal SCFAs

**Figure 6:**
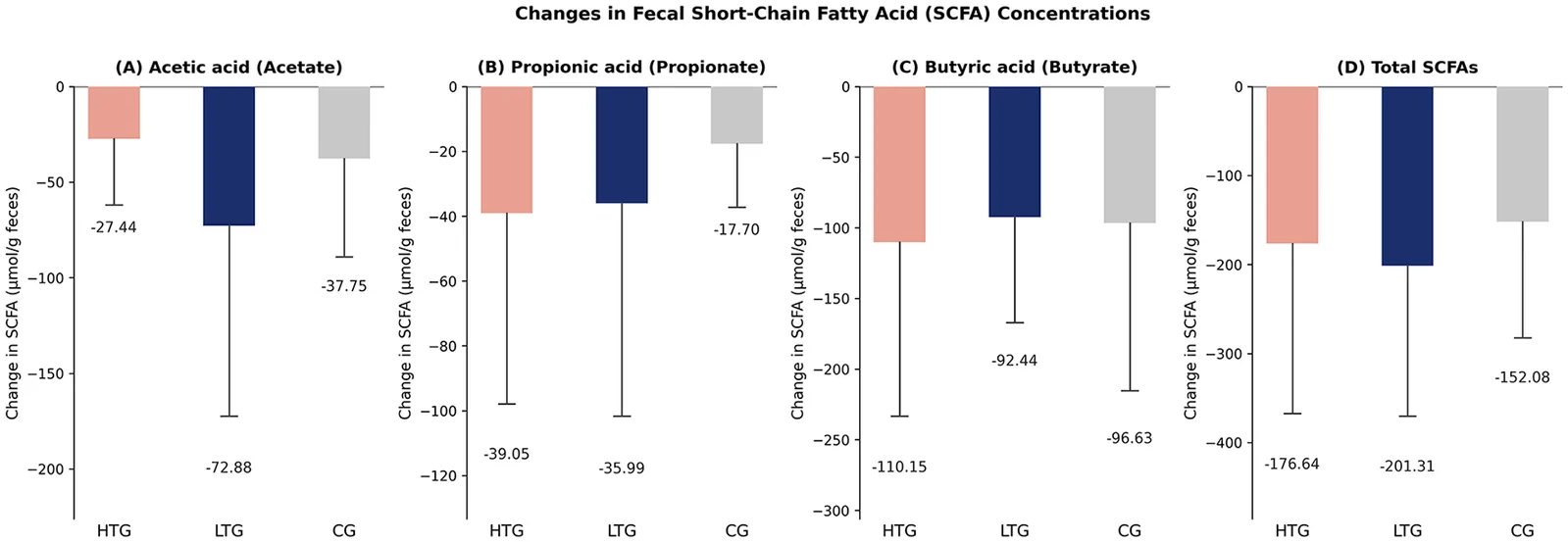
Changes in fecal short-chain fatty acid (SCFA) concentrations following the 8-week intervention. **(A)** Acetic acid (Acetate); **(B)** Propionic acid (Propionate); **(C)** Butyric acid (Butyrate); **(D)** Total SCFAs. Data are presented as mean change from baseline (Δ). HTG, Traditional Gochujang with high beneficial microorganism content; LTG, Traditional Gochujang with low beneficial microorganism content; CG, Commercial Gochujang.

All groups demonstrated significant within-group reductions in acetate, propionate, butyrate, and total SCFAs over the intervention period (Table 7). These changes occurred in parallel across the three formulation groups, with no significant between-group differences reported (Figure 6).

**Table 7:** Exploratory changes in fecal short-chain fatty acids.

Value	Group								
	HTG (***n*** = **20)**			**LTG (*****n*** = **20)**			**CG (*****n*** = **19)**		
	**Before**	**After**	***p*** **-value**	**Before**	**After**	***p*** **-value**	**Before**	**After**	***p*** **-value**
Acetic acid	54.01 ± 34.47	26.57 ± 15.03	0.004	99.56 ± 99.47	26.68 ± 11.96	0.004	64.00 ± 51.26	26.25 ± 14.84	0.005
Propionic acid	52.32 ± 58.85	13.27 ± 8.28	0.006	51.94 ± 65.66	15.95 ± 8.27	0.027	33.72 ± 19.53	16.02 ± 7.66	0.004
Butyric acid	129.13 ± 123.10	18.98 ± 11.25	0.001	114.28 ± 74.71	21.84 ± 10.65	< 0.0001	125.17 ± 118.55	28.54 ± 22.59	0.003
Total SCFAs	235.46 ± 195.71	58.81 ± 28.15	0.001	265.78 ± 170.17	64.47 ± 24.17	< 0.0001	222.89 ± 135.94	70.81 ± 27.71	< 0.0001

Values are expressed as the mean ± standard deviation. The statistical significance of within-group changes from before to after the intervention was determined using a paired *t*-test. Short-chain fatty acid (SCFA) concentrations were analyzed as exploratory markers reflecting gut microbial metabolic activity. HTG, Traditional Gochujang with high beneficial microbe content; LTG, Traditional Gochujang with low beneficial microbe content; CG, Commercially prepared Gochujang.

### 3.6 Safety and tolerability assessment

Throughout the 8-week intervention, vital signs (SBP, DBP, pulse) remained stable in all groups. Key liver enzymes (GGT, AST, and ALT) and other safety laboratory parameters (CBC, creatinine, and others) showed no clinically important changes either. Some parameters changed slightly from baseline in one or more groups, but all values remained within normal reference ranges (Table 8). No adverse events related to the intervention were reported. Overall, the Gochujang formulations were well-tolerated over the study period.

**Table 8:** Safety and tolerability assessment of Gochujang.

Value	Group								
	**HTG (*****n*** = **20)**			**LTG (*****n*** = **20)**			**CG (*****n*** = **19)**		
	**Before**	**After**	***p*** **-value**	**Before**	**After**	***p*** **-value**	**Before**	**After**	***p*** **-value**
SBP (mmHg)	130.2 ± 12.99	128.15 ± 11.94	0.434	128.05 ± 13.2	129.7 ± 12.59	0.621	133.68 ± 9.67	129.63 ± 12.11	0.085
DBP (mmHg)	78.6 ± 7.62	76.1 ± 7.32	0.221	76 ± 9.96	74.05 ± 9.89	0.253	76.79 ± 9.42	74 ± 7.97	0.064
Pulse (bpm)	73.7 ± 7.85	76.75 ± 11.62	0.164	71.1 ± 8.26	70.35 ± 7.65	0.713	75.37 ± 9.8	74.58 ± 8.03	0.662
WBC (10^3^/μL)	6.61 ± 1.42	6.65 ± 1.45	0.903	6.05 ± 1.21	6.01 ± 1.24	0.882	6.51 ± 0.94	6.15 ± 1.23	0.099
RBC (106/μL)	4.55 ± 0.33	4.53 ± 0.42	0.646	4.47 ± 0.25	4.36 ± 0.3	0.083	4.46 ± 0.25	4.35 ± 0.26	0.013
Hemoglobin (g/dL)	13.77 ± 0.88	13.54 ± 0.84	0.029	13.47 ± 0.82	13.39 ± 1.06	0.552	13.46 ± 0.62	13.11 ± 0.55	0.013
Hematocrit (%)	41.11 ± 2.33	40.06 ± 2.48	0.002	40.17 ± 2.19	40.74 ± 5.91	0.700	40.4 ± 1.78	39.12 ± 1.55	0.003
GGT (U/L)	23 ± 10.74	23.45 ± 12.02	0.734	28.7 ± 17.67	32.42 ± 21.06	0.123	28.58 ± 20.1	26.95 ± 16.03	0.517
AST (U/L)	30.5 ± 6.8	29.6 ± 5.1	0.442	28.2 ± 6.54	27.9 ± 7.46	0.844	29.42 ± 5.85	29.21 ± 10.17	0.902
ALT (U/L)	25.45 ± 8.19	26.1 ± 7.4	0.650	23.3 ± 8.81	26.75 ± 10.24	0.110	25.42 ± 8.6	26.58 ± 13.66	0.569
BUN (mg/dL)	14.44 ± 3.2	13.97 ± 2.67	0.554	14.34 ± 4.27	15.11 ± 4.14	0.267	15.95 ± 4.33	14.46 ± 3.82	0.063
Creatinine (mg/dL)	0.61 ± 0.11	0.65 ± 0.11	0.056	0.65 ± 0.1	0.69 ± 0.1	0.048	0.7 ± 0.15	0.7 ± 0.14	0.954
Uric acid (mg/dL)	4.78 ± 1.03	4.72 ± 0.93	0.705	4.65 ± 1.19	4.89 ± 1.35	0.027	4.78 ± 1.21	4.49 ± 1.08	0.035
T. protein (g/dL)	7.08 ± 0.42	7.11 ± 0.42	0.631	7.08 ± 0.29	6.98 ± 0.42	0.110	7.06 ± 0.35	6.91 ± 0.35	0.044
Albumin (g/dL)	4.36 ± 0.19	4.35 ± 0.21	0.797	4.31 ± 0.18	4.18 ± 0.24	0.004	4.27 ± 0.24	4.19 ± 0.18	0.114
T. bilirubin (mg/dL)	0.79 ± 0.28	0.81 ± 0.31	0.534	0.84 ± 0.29	0.77 ± 0.22	0.131	0.67 ± 0.12	0.69 ± 0.13	0.604
LD (U/L)	194.85 ± 47.14	193 ± 38.1	0.706	174.8 ± 23.18	172.55 ± 26.01	0.507	186.63 ± 24.31	190.79 ± 27.24	0.412
ALP (U/L)	71.9 ± 16.13	72.1 ± 17.78	0.900	72.5 ± 21.78	72.65 ± 22.51	0.931	70.95 ± 14.47	69.58 ± 13.63	0.390
CK (U/L)	115.4 ± 41.3	103.55 ± 39.85	0.149	94.95 ± 37.77	96.65 ± 38.85	0.700	110.26 ± 38.18	121.74 ± 46.91	0.236

ALP, Alkaline phosphatase; ALT, Alanine aminotransferase; AST, Aspartate aminotransferase; BUN, Blood urea nitrogen; CK, Creatine kinase; DBP, Diastolic blood pressure; GGT, Gamma-glutamyl transferase; LD, Lactate dehydrogenase; RBC, Red blood cell; SBP, Systolic blood pressure; T. bilirubin, Total bilirubin; T. protein, Total protein; WBC, White blood cell; HTG, Traditional Gochujang with high beneficial microbe content; LTG, Traditional Gochujang with low beneficial microbe content; CG, Commercially prepared Gochujang. Values are expressed as mean ± standard deviation. The statistical significance of within-group changes from before to after the intervention was determined using a paired *t*-test. *P*-values < 0.05 were considered statistically significant.

## 4 Discussion

This 8-week, randomized, double-blind, three-arm comparative trial is first to evaluate three Gochujang formulations in postmenopausal women and was designed to compare formulations rather than to test Gochujang against a placebo. No statistically significant differences were observed among the three formulations (HTG, LTG, and CG) for the primary endpoint. Because between-group comparison is the principal inferential basis of a randomized design, this absence of between-group differences—which extended to the secondary endpoints of body composition and metabolic biomarkers, none of which differed significantly among formulations—is the primary finding of the trial. Among the exploratory endpoints, the only nominally significant between-group difference was a greater reduction in bacteria classified as beneficial in LTG than in HTG (*p* = 0.046; Table 3); this isolated result would not withstand correction for multiple comparisons and is not interpreted as a formulation-specific effect. Within-group reductions in KMI scores were observed consistently across all three groups, with a comparable magnitude of improvement across formulations. This pattern is consistent with the possibility of shared biological effects of Gochujang components, although causal efficacy cannot be inferred from the present comparative design. The within-group changes are reported for completeness and transparency but, on their own, cannot establish treatment efficacy and cannot exclude contributions from placebo response or regression to the mean.

Similarly, significant within-group reductions in PBF were observed across all three groups, with no significant between-group differences, consistent with body composition effects being shared across formulations rather than being formulation-specific. Systemic inflammatory and metabolic biomarkers remained largely unchanged. Fecal SCFA concentrations showed pronounced within-group reductions across all three arms; these within-group changes cannot be causally attributed to Gochujang and may be compatible with an effect of shared bioactive compounds. These may include capsaicinoid-mediated TRPV1 activation, which promotes thermogenesis and white adipose tissue browning, together with capsaicin-induced strengthening of intestinal barrier function that may reduce the chronic low-grade inflammation contributing to adiposity in postmenopausal women (11).

Two group-specific observations warrant further research: a reduction in absolute body fat mass in HTG and an increase in fat-free mass with a corresponding rise in basal metabolic rate in LTG (Table 4); these divergences may reflect baseline variability or unmeasured differences in physical activity rather than formulation-specific effects.

KMI scores decreased significantly within all three arms (Section 3.1; Table 2). As the trial did not include a non-Gochujang placebo arm, these within-group changes should be interpreted in the context of shared Gochujang exposure; their consistency across three independently randomized arms may suggest that Gochujang consumption is associated with reduced menopausal symptom severity, a possibility requiring confirmation in future placebo-controlled trials. These reductions are consistent with the shared bioactive matrix of Gochujang, which involves fermentation-enhanced isoflavone aglycones and capsaicinoids, although this mechanistic account is proposed rather than demonstrated. The absence of between-group differences indicates that the observed changes were not dependent on formulation-specific microbial composition. Reductions were not confined to a single domain: consistent within-group decreases were observed across the principal menopause-specific symptom clusters, including vasomotor symptoms and paresthesia, in all three arms (Table 2), indicating that the aggregate KMI improvement reflected broadly distributed symptom relief rather than change in an isolated component. In contrast, the generic health-related quality-of-life measure (EQ-5D index) did not change significantly in any group, indicating that the symptom-level improvements captured by the KMI were not accompanied by a detectable change in generic quality of life over this 8-week period. This discordance may reflect the limited responsiveness of a generic utility instrument to menopause-specific symptom change, the short intervention duration, or a ceiling effect, and underscores the value of incorporating menopause-specific quality-of-life instruments in future trials.

Previous placebo-controlled trials of phytoestrogen-containing interventions have demonstrated reductions in menopausal symptom severity relative to placebo (19, 20). The consistent within-group reductions in KMI scores observed in the present study are directionally aligned with these prior findings and are consistent with a possible role for isoflavone aglycone-mediated symptom modulation. Of note, the fermentation process in Gochujang substantially increases the bioavailability of isoflavone aglycones (approximately 75–86%) relative to non-fermented soy products, which may enhance their estrogenic activity compared with conventional isoflavone supplements (19, 20). The present evidence is consistent with the possibility that this mechanism extends to postmenopausal women consuming Gochujang (fermented food), although it does not establish it.

Prior clinical studies have demonstrated that capsaicinoid-containing compounds reduce body fat through TRPV1-mediated thermogenesis and enhanced lipid oxidation (21, 22). The significant within-group reductions in PBF observed across all three Gochujang groups in the present study are consistent with these reported mechanisms. Capsaicinoids in Gochujang activate TRPV1, increasing energy expenditure and promoting white adipose tissue browning; additionally, capsaicin has been shown to enhance intestinal barrier integrity by upregulating tight junction proteins, thereby reducing chronic low-grade inflammation that underlies metabolic dysfunction in postmenopausal women (11). The absence of between-group differences in body composition changes indicates that any change is unlikely to depend on formulation-specific microbial content; however, in the absence of a placebo arm these within-group changes cannot be causally attributed to capsaicinoid content and should be regarded as hypothesis-generating.

In addition, fermentation-derived isoflavone aglycones exhibit enhanced bioavailability compared with their glycoside forms and exert estrogen-like biological effects through interactions with estrogen receptors. The relationship between isoflavones and gut microbiota is bidirectional: while isoflavone aglycones influence gut microbial composition, intestinal bacteria play an essential role in isoflavone biotransformation, including the conversion of daidzein to equol, the most estrogenically active isoflavone metabolite (23). Clavel et al. demonstrated in a randomized, double-blind, placebo-controlled study that isoflavone supplementation in postmenopausal women selectively stimulated beneficial microbial populations, including the *Clostridium coccoides–Eubacterium rectale* cluster, the *Lactobacillus–Enterococcus* group, and the *Bifidobacterium* genus (23).

The taxonomic shifts observed in the present study are consistent with these known isoflavone–microbiota interactions and are consistent with the interpretation that Gochujang bioactives may modulate gut microbial ecology in postmenopausal women. As serum estradiol, FSH, and LH were not measured and participants' equol-producer status was not determined, any estrogen-related mechanisms remain speculative and cannot be supported by the present dataset.

SCFAs are the primary end-products of anaerobic bacterial fermentation of dietary substrates in the colon, and serve as key mediators linking gut microbiota to host metabolic and immune regulation (18, 24, 25). SCFA production capacity is directly determined by the abundance of specific bacterial taxa: *Faecalibacterium prausnitzii, Roseburia* species, and members of the Lachnospiraceae family are the principal butyrate producers, while *Bifidobacterium* and *Lactobacillus* species contribute predominantly to acetate production (18, 24). Capsaicin is a primary bioactive component of Gochujang and has been specifically shown to increase butyragenic bacteria and reduce LPS-producing bacteria, thereby shifting the microbial metabolite landscape toward a more favorable SCFA profile (26). Similarly, isoflavones have been demonstrated to alter dominant intestinal microbiota in postmenopausal women in ways that could influence SCFA production capacity (23). However, fecal SCFA concentrations represent the net balance of microbial production, host colonic absorption, and intestinal transit time, rather than a direct measure of total SCFA synthesis capacity (27, 28). Consequently, changes in microbial community structure do not necessarily translate into proportional changes in fecal SCFA levels. In the present study, the pronounced reductions in fecal SCFA concentrations across all three groups may reflect this complex balance rather than a global reduction in microbial metabolic activity. The parallel decrease in SCFA concentrations across all arms, coinciding with shifts in microbial composition, may be explained by altered colonic absorption dynamics or intestinal transit rather than simply reduced microbial fermentation capacity, although this hypothesis was not directly evaluated. These findings highlight the critical importance of not equating fecal SCFA concentrations with colonic SCFA production when interpreting dietary intervention outcomes (17, 23, 26, 29).

The apparent discordance between microbiota shifts and fecal SCFA changes observed in the present study is consistent with findings from several rigorous dietary intervention studies. Oliver et al. demonstrated that a high-fiber, whole-food dietary intervention in healthy adults altered gut microbiome composition, yet produced no corresponding changes in fecal SCFA concentrations, which the authors attributed to increased host absorption of SCFAs rather than reduced production (30). Similarly, Wastyk et al., in a landmark 17-week randomized trial, showed that a high-fermented-food diet increased microbiota diversity and decreased inflammatory markers, but metabolic and SCFA responses were not uniformly concordant with compositional changes (31). Brahe et al. reported microbiota modulation without parallel metabolic outcome changes in postmenopausal women with obesity following dietary intervention (32). Collectively, these findings confirm that microbiome composition, metabolite profiles, and clinical outcomes do not exhibit a simple linear relationship in human studies (30–33), suggesting a need for integrated functional metagenomic and metabolomic analyses beyond taxonomic classification alone to fully characterize the biological consequences of fermented food interventions.

Although the within-group changes observed in the present study are biologically compatible with reported mechanisms for both capsaicinoids and isoflavone aglycones, the absence of significant between-group differences in SCFA concentrations and microbiota composition is consistent with these effects being shared across formulations rather than driven by formulation-specific microbial differences within the 8-week intervention timeframe. Whether longer intervention durations or higher-dose formulations might reveal formulation-specific effects remains an important question for future research.

SCFAs are central microbial metabolites involved in host metabolic and immune regulation, with butyrate serving as the primary energy source for colonocytes, propionate regulating hepatic gluconeogenesis, and acetate influencing peripheral lipid metabolism (18, 24, 25). The significant within-group reductions in acetate, propionate, butyrate, and total SCFA concentrations observed across all three Gochujang groups in this trial are noteworthy. Given that fecal SCFA levels represent the net balance of microbial production, colonic epithelial absorption, and intestinal transit rather than absolute synthesis capacity (27, 28), these reductions may reflect enhanced host utilization of SCFAs rather than reduced microbial fermentation activity. We emphasize that this is a hypothesis, not a demonstrated mechanism: the present study did not measure intestinal SCFA absorption, epithelial transport, gut permeability, transit time, or plasma or metabolomic SCFA profiles, and therefore cannot distinguish enhanced host utilization from reduced microbial production or altered intestinal transit. This explanation is offered as one plausible hypothesis to be tested in future studies incorporating these measures.

Exploratory analyses further revealed significant within-group shifts in gut microbial composition across all three groups, with a notable and statistically significant reduction in bacteria classified as “harmful” in the HTG group (*p* = 0.013), alongside reductions in “beneficial” bacteria in both HTG (*p* = 0.037) and LTG (*p* = 0.001). These taxonomic shifts are consistent with the established effects of both capsaicinoids and isoflavones on gut microbial community structure (23, 26, 29). However, the classifications of bacteria as “beneficial” or “harmful” are simplified and context-dependent and should not be interpreted as definitive indicators of microbial function; taxonomic shifts, moreover, do not necessarily imply functional changes. Because no shotgun metagenomic or metabolomic profiling was performed, any functional interpretation of these compositional changes remains provisional and requires confirmation in future studies employing shotgun metagenomics and untargeted metabolomics.

Between-group comparisons of microbiota change suggested a greater numerical reduction in bacteria classified as “harmful” in the HTG group compared with LTG and CG, although formal pairwise comparisons did not reach statistical significance after accounting for the exploratory nature of these analyses. This pattern is biologically plausible given that HTG contains a higher viable microbial content than the other formulations, and may have directly competed with or suppressed resident harmful taxa through competitive exclusion or bacteriocin-mediated inhibition. These exploratory findings provide a rationale for future trials designed to evaluate the impact of Gochujang microbial content on gut microbiome composition using adequately powered, placebo-controlled designs with comprehensive functional metagenomic approaches.

The short-term safety profile of all three Gochujang formulations was excellent, representing a clinically meaningful finding in its own right given that postmenopausal women carry elevated cardiometabolic risk. No adverse events related to the intervention were reported across the 8-week study period. Key safety parameters remained within normal reference ranges throughout the study in all three groups. Notably, despite the substantial sodium content of Gochujang (estimated 440–550 mg/day in this study), no significant changes in SBP or DBP were observed in any group. This finding is consistent with prior experimental evidence that Gochujang exerts anti-hypertensive effects through modulation of the renin–angiotensin–aldosterone system, which may counteract the pressor effects of its sodium content (13, 34). These data provide the first clinical evidence supporting the tolerability of regular Gochujang consumption in postmenopausal women, which is a prerequisite for the larger, placebo-controlled trials required to establish efficacy.

This study has some limitations. First, the absence of a non-Gochujang placebo control limits causal inference regarding Gochujang-specific efficacy. However, this study was specifically designed as a formulation-comparative trial to evaluate between-formulation differences in a previously unstudied population, so the lack of a placebo arm reflects the intended scientific question rather than a methodological oversight. The consistent and statistically significant within-group reductions in the KMI scores across all three independently randomized arms, combined with the well-established biological plausibility of Gochujang bioactives, nonetheless provide preliminary evidence for Gochujang-associated symptom improvement.

Second, the relatively small sample size (*n* = 59) may have limited statistical power to detect subtle between-group differences; the absence of significant between-group differences should not be interpreted as proof of equivalence, as this study was not powered for formal non-inferiority testing. Third, the 8-week intervention period may be insufficient to capture longer-term endocrine or microbiome adaptations, given that isoflavone effects on menopausal symptoms have been reported to emerge gradually over 12 weeks or longer (19, 20). Fourth, dietary intake and physical activity were not strictly controlled, and no structured dietary assessment was performed during the intervention. In particular, intakes of soy and other isoflavone-containing foods, other fermented foods, dietary fiber, probiotics, and alcohol were not quantified. Because these components strongly influence both menopausal symptoms and gut microbiota, their variation constitutes a potential source of residual confounding, and the observed changes should be interpreted with this limitation in mind.

Fifth, microbiome analyses were taxonomic in nature and lacked functional metagenomic validation; future studies should integrate shotgun metagenomics with targeted metabolomics to fully characterize the host–microbiome interaction. Finally, hormonal and neuroendocrine biomarkers—including serum estradiol, FSH, LH, and urinary equol—were not assessed. The estrogen-related mechanisms discussed above are therefore proposed based on prior literature rather than direct measurement in this cohort, and mechanistic inference should be regarded as tentative.

The outcome hierarchy applied in this report differs from the hierarchy that was prospectively specified, and the difference is described in full in Section 2.3. Change in gut microbiota was one of two co-primary outcomes in the trial registration and was likewise listed among the primary outcome measures, together with fecal SCFA concentrations, in the IRB-approved protocol, whereas both are analyzed here as exploratory outcomes. No amendment to the protocol or to the trial registration was made, and this reclassification is therefore *post hoc*. We accordingly report all microbiome-related outcomes in full and irrespective of statistical significance, and no statistically significant between-group differences were observed for these endpoints.

Clinically, these findings indicate that short-term Gochujang consumption in postmenopausal women is safe, well-tolerated, and associated with statistically significant within-group reductions in menopausal symptom severity and PBF across all three formulations tested. The comparable changes across groups provide no evidence that outcomes differed according to formulation. Whether Gochujang itself provides clinical benefit remains to be established in placebo-controlled trials. Importantly, the present findings also illustrate that variations in microbial composition and preparation methods do not necessarily translate into differential clinical outcomes over short-term periods (30–33) and highlight the need for integrated functional and longitudinal approaches in future research.

## 5 Conclusion

In conclusion, this randomized, double-blind comparative trial is first to evaluate Gochujang formulations in postmenopausal women and suggests that 8-week consumption of three distinct Gochujang formulations is safe, well-tolerated, and associated with significant reductions in menopausal symptom severity (KMI scores) and PBF, with comparable effects across all three formulations. Exploratory microbiome analyses identified within-group taxonomic changes, but did not provide robust evidence of formulation-specific differences. The pronounced within-group reductions in fecal SCFA concentrations are consistent with an interaction between Gochujang consumption and gut microbiome function, and should be interpreted as hypothesis-generating. These findings provide a foundation for larger, placebo-controlled randomized trials that incorporate endocrine biomarkers (serum estradiol, FSH, urinary equol), functional metagenomics, and extended follow-up periods to establish the clinical efficacy and mechanistic basis of Gochujang as a dietary intervention for postmenopausal health.

## Data Availability

The raw data supporting the conclusions of this article will be made available by the authors, without undue reservation.
