# Supplementary material for: Effects of gochujang formulations on menopausal symptoms and gut microbiota in postmenopausal women: an 8-week randomized, double-blind trial
Source: Front Nutr. 2026 Sep 10;13:1849944. doi: 10.3389/fnut.2026.1849944 (PMC13600830; doi:10.3389/fnut.2026.1849944)
Supplement: Supplementary file 1 [file Data_Sheet_1.PDF]

인 체 적 용 시 험 계 획 서

고추발효물(고추장분말)의 장내 균총 및 갱년기 여성건강에 대한
유효성 및 안전성을 평가하기 위한 8 주, 무작위배정, 이중눈가림 인체적용시험
A 8 weeks, randomized, double-blind human trial to evaluate the efficacy and safety of Fermented hot pepper(Gochujang powder)on intestinal microflora and menopausal women's health

인체적용시험 의뢰기관: (재)발효미생물산업진흥원

(재)발효미생물산업진흥원
MIFI Microbial Institute for Fermentation Industry

인체적용시험용제품 1: 고추발효물 TCG25-26(유용미생물 고함량 고추장분말)
인체적용시험용제품 2: 고추발효물 TMG25-2(유용미생물 저함량 고추장분말)
인체적용시험용제품 3: 고추발효물 TFG25-2(시판용 고추장분말)
인체적용시험계획서번호: MIFI-MWH-GCJ
버전: 1.0
인체적용시험계획서 작성일: 2025.03.20
단계: 인체적용시험

Confidentiality statement

이 인체적용시험 계획서에 포함된 정보는 시험기관장, 시험책임자, 시험담당자, 제품관리자, 관련 행정기관, 기관생명윤리위원회(IRB)를 위한 guidance 로 단독으로 계획된 것입니다.
이 계획서는 (재)발효미생물산업진흥원의 서면동의를 받지 않은 채 시험에 관련되지 않는 자에게 공개될 수 없습니다. 단 연구대상자 서면동의를 받기 위해 필요한 경우는 예외일 수 있습니다. 이 계획서를 제공 받은 분은 문서 기밀사항을 보장해 주시기 바랍니다.

● 용어 및 약어의 정의

용어 및 약어	정의
ADR	Adverse Drug Reaction
AE	Adverse Event
ALP	Alkaline Phosphatase
ALT	Alanine Transaminase
ANCOVA	Analysis of Covariance
AST	Aspartate Transaminase
BMI	Body Mass Index
BUN	Blood Urea Nitrogen
CK	Creatine Kinase
CRF	Case Report Form
CRP	High sensitivity C-reactive protein
ESR	Erythrocyte sedinentation rate
EQ-5D	EuroQol-5 dimension
FA	Full Analysis
GCP	Good Clinical Practice
GPAQ	Global Physical Activity Questionnaire
Hb	Hemoglobin
Hct	Hematocrit
HDL	High Density Lipoprotein
HOMA-IR	Homeostasis model assessment for insulin resistance
HOMA-β	Homeostatic model assessment of β cell function
ICD	Informed Consent Document
ICH	International Conference on Harmonization
ID	Identification

IRB	Institutional Review Board
KI	Modified Kupperman's index
LD	Lactate dehydrogenase
LDL	Low Density Lipoprotein
LOCF	Last Observation Carried Forward
PLT	Platelets Count
PP	Per Protocol
QUICKI	Quantitative insulin check index
RBC	Red Blood Cell
RM-ANOVA	Repeated Measure Analysis of variance
SAE	Serious Adverse Event
SD, std	Standard Deviation
WBC	White Blood Cell
WHR	Waist Hip Ratio

● 인체적용시험계획서 개요

제 목	고추발효물(고추장분말)의 장내 균총 및 갱년기 여성건강에 대한 유효성 및 안전성을 평가하기 위한 8 주, 무작위배정, 이중눈가림 인체적용시험
목 적	고추발효물(고추장분말)을 8 주간 섭취하였을 때 장내 균총 및 갱년기 여성건강에 대한 유효성 및 안전성을 비교 평가하고자 한다.
계획서번호	MIFI-MWH-GCJ
시 험 단 계	인체적용시험
시 험 기 간	IRB 승인일로부터 12 개월
시험디자인	8 주, 무작위배정, 이중눈가림 인체적용시험
실 시 기 관 및 연 구 자	<별첨 1> 인체적용시험 실시기관, 시험책임자, 시험담당자 및 인체적용시험용제품 관리자 목록 참조
의 뢰 자	(재)발효미생물산업진흥원
연구대상자 수	총 60 명<br>•고추발효물 TCG25-26(유용미생물 고함량 고추장분말)군: 20 명<br>•고추발효물 TMG25-2(유용미생물 저함량 고추장분말)군: 20 명<br>•고추발효물 TFG25-2(시판용 고추장분말)군: 20 명
연구대상자 선 정 기 준	1) 스크리닝 검사 당시 연령이 만 45 세 이상, 70 세 이하인 성인 여성<br>2) 폐경(12 개월 이상 무월경 상태)인 자<br>3) 쿠퍼만지수(Modified Kupperman’s index)점수가 15 점 이상인 자<br>4) 스크리닝 검사 당시 BMI 가 23 kg/m^2 이상인 자<br>5) 본 인체적용시험에 대한 자세한 설명을 듣고 완전히 이해한 후, 자의로 참여를 결정하고 주의사항을 준수하기로 서면

	동의한 자
연구대상자 제외기준	1) 호르몬 대체요법을 시행하고 있거나, 최근 6 개월 이내에 호르몬 대체요법을 시행한 경험이 있는 자<br>2) 자궁내막증식증, 자궁암, 자궁내막암, 유방암 또는 유방질환의증, 성호르몬 관련암 등이 있거나 과거력이 있는 자<br>3) 난소 기능에 영향을 줄 수 있는 화학요법이나 방사선요법을 받거나 받은 경험이 있는 자<br>4) 양측난소절제술의 과거력이 있는 자<br>5) 치료를 요하는 임상적으로 유의한 급성 또는 만성 심뇌혈관계, 내분비계, 면역계, 호흡기계, 간담도계, 신장 및 비뇨기계, 신경정신계, 근골격계, 염증성 및 혈액·종양성, 위장관계 질환 등이 있는 자<br>6) 스크리닝 검사 전 1 개월 이내에 장내 균총 및 갱년기 여성건강과 관련된 의약품 및 건강기능식품을 복용한 자(병용금지약물 참고)<br>7) 고추, 고추장 성분에 유의한 과민반응의 병력이 있는 자<br>8) 스크리닝 검사 전 3 개월 이내에 항정신병 약물치료를 받은 경험이 있는 자<br>9) 알코올 중독 또는 약물 남용이 있거나 의심되는 자<br>10) 스크리닝 검사 전 3 개월 이내에 타 인체적용시험에 참여한 자<br>11) 진단검사의학 검사에서 다음에 해당하는 결과를 보이는 자<br>☞ AST, ALT > 참고범위 상한치의 3 배<br>☞ Serum Creatinine > 2.0 mg/dL<br>12) 진단검사의학 검사결과를 비롯한 기타 사유로 인하여 시험책임자가 연구 참여에 부적합하다고 판단한 자
섭취방법 및 용량	연구대상자는 다음 세 군 중 하나에 무작위배정 되어 8 주 동안 인체적용시험용제품을 1 일 2 회 경구 섭취한다.<br>• 고추발효물 TCG25-26(유용미생물 고함량 고추장분말)군: 1일 2회, 1회 3포, 아침/저녁 식후 경구섭취(25.2 g/day, 고추장분말로써 19 g/day)<br>• 고추발효물 TMG25-2(유용미생물 저함량 고추장분말)군: 1일

	2회, 1회 3포, 아침/저녁 식후 경구섭취(25.2 g/day, 고추장분말로써 19 g/day)<br>• 고추발효물 TFG25-2(시판용 고추장분말)군: 1일 2회, 1회 3포, 아침/저녁 식후 경구섭취(25.2 g/day, 고추장분말로써 19 g/day)
섭 취 기 간	8 주
시 험 방 법	시험자는 서면동의서를 작성한 자원자에 한하여 스크리닝 검사를 시행하여 선정기준에 부합하는 연구대상자를 선정한다. 연구대상자는 스크리닝 방문으로부터 4 주 이내에 1 차 방문하여 연구대상자 선정/제외기준 적합성을 재검토 받은 후 인체적용시험에 등록되며, 고추발효물 TCG25-26(유용미생물 고함량 고추장분말)군, 고추발효물 TMG25-2(유용미생물 저함량 고추장분말)군, 고추발효물 TFG25-2(시판용 고추장분말)군에 무작위배정 되어 1 차 방문일까지 기초평가(Baseline)를 완료한다. 이후 시험용제품을 하루 2 번씩 8 주간 매일 섭취하면서 4 주마다 수행기관에 방문하여 유효성평가, 활력징후, 약물투여력 및 의학적상태변화 조사, 이상사례 조사 등을 수행한다.<br>시험자는 인체적용시험용제품 최종 섭취 후 혹은 조기종료 방문 후 필요에 따라 연구대상자 추적 관찰을 실시할 수 있다.

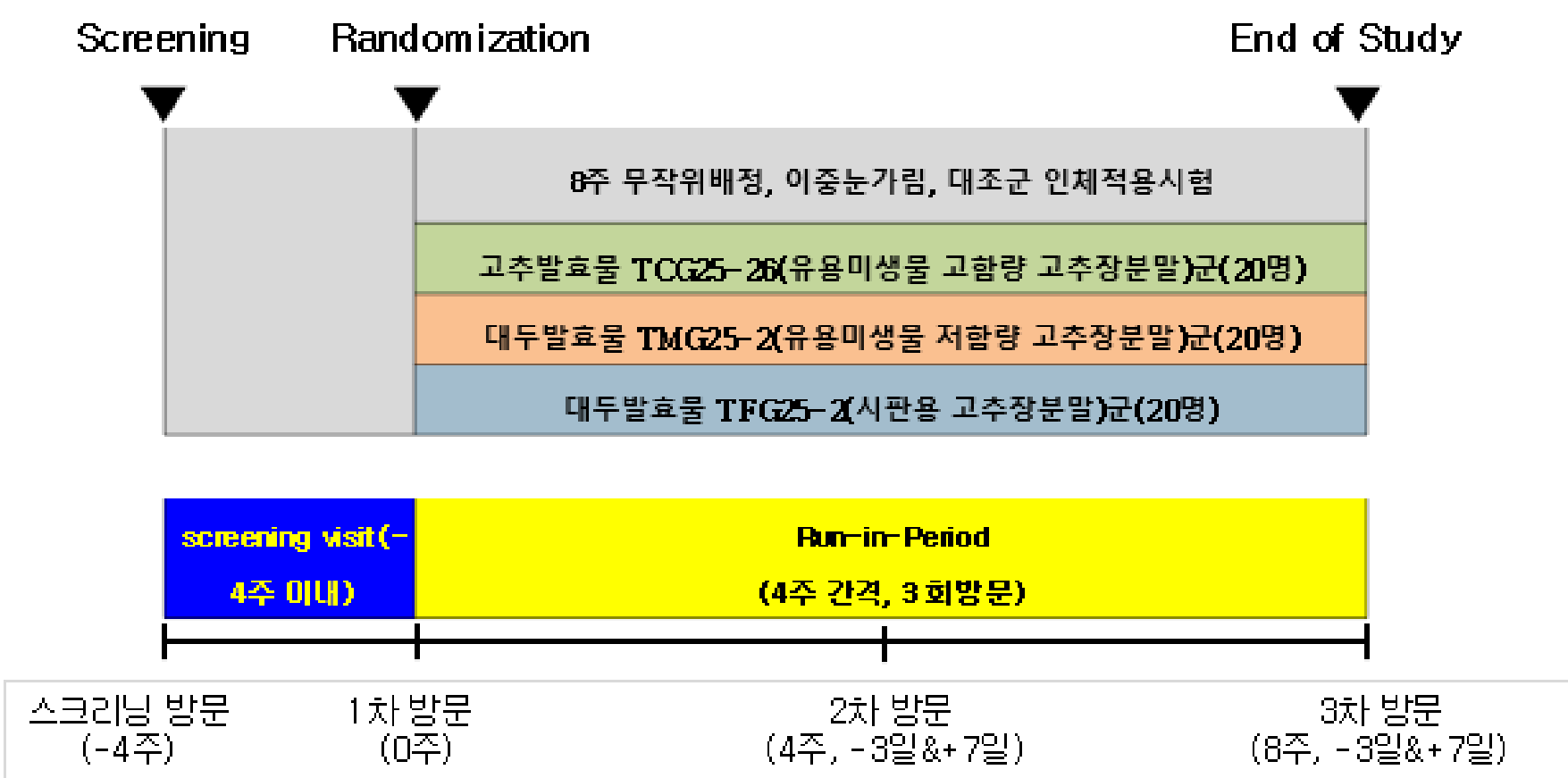

유 효 성 평 가	◆ 1차 유효성 평가<br>• 장내 균총 변화<br>• 쿠퍼만지수(KI; Modified Kupperman's index)<br>◆ 2차 유효성 평가<br>• 체성분 분석: 체중, 체질량지수, 체지방량, 체지방률, 제지방량, 복부지방률, 기초대사율<br>• 신체계측지표: 허리둘레, 엉덩이둘레, 허리-엉덩이둘레비<br>• 지질대사지표: Total cholesterol, Triglyceride, LDL-C, HDL-C, Non HDL-C<br>• 염증관련지표: CRP, ESR<br>• 혈당관련지표: glucose, insulin, HOMA-IR, HOMA-β, QUICKI<br>• 단쇄지방산<br>• EQ-5D(EuroQol-5 dimension)
안 전 성 평 가	◆ 자·타각 증상 등 이상사례 모니터링<br>◆ 진단검사의학 검사<br>◆ 활력징후, 신체검진
통계분석	○ 분석에 사용할 프로그램은 Window 용 SPSS 또는 SAS 를 이용하고, 통계학적 유의수준은 *p* 값이 0.05 미만으로 설정한다.<br>○ 유효성 평가에 대한 자료는 PP 군을 주 분석대상으로 하되, ITT 군 및 FA 군을 추가적으로 분석하여 유효성을 평가할 수 있다. 안전성 평가에 대한 자료는 Safety 군에 대해 분석하여 본 인체적용시험용제품에 대한 안전성을 평가한다.<br>○ 집단 간 기저치 분포의 차이가 있는지를 확인하기 위한 검정은 Chi-Square test 또는 Fisher's exact test 와 Independent t-test 를 이용한다.<br>○ 유효성 분석/안전성 분석<br><u>유효성 평가 변수</u><br>1 차 유효성 평가 변수(장내 균총 변화, 쿠퍼만지수) 및 2 차 유효성 평가 변수(체성분 분석, 신체계측지표, 지질대사지표,

염증관련지표, 혈당관련지표, 단쇄지방산, EQ-5D)결과에 대해서 섭취군 별로 기술통계량을 제시하고, 섭취군 간 비교와 섭취군 내 비교를 실시한다. 섭취군 간 비교의 경우, 정규성 검정을 수행하여 정규성을 만족하였을 때, 변화량(섭취 8 주 후-섭취 전)에 대해서 independent t-test 를 이용하고, 만족하지 못하였을 때 Mann-Whitney U test 를 이용하여 분석한다. 고추발효물 TCG25-26(유용미생물 고함량 고추장분말)군과 고추발효물 TFG25-2(시판용 고추장분말)군, 고추발효물 TMG25-2(유용미생물 저함량 고추장분말)군과 고추발효물 TFG25-2(시판용 고추장분말)군, 고추발효물 TCG25-26(유용미생물 고함량 고추장분말)군과 고추발효물 TMG25-2(유용미생물 저함량 고추장분말)군 간 변화량(섭취 8 주 후-섭취 전)에 대해서 independent t test 또는 Mann-Whitney U test 를 이용하여 분석한다.

또한 고추발효물 TCG25-26(유용미생물 고함량 고추장분말)군, 고추발효물 TMG25-2(유용미생물 저함량 고추장분말)군, 고추발효물 TFG25-2(시판용 고추장분말)군 간 차이에 대해서 정규성 만족하였을 때 Linear mixed model 을 이용하고, 만족하지 못하였을 때 Friedman’s test 를 이용하여 분석한다.

섭취군 내 비교의 경우, 정규성 검정을 수행하여 정규성을 만족하였을 때, 섭취 전 대비 8 주 시점의 변화에 대하여 paired t test 를 이용하고, 만족하지 못하였을 때 Wilcoxon signed-rank test 를 이용하여 분석한다. 각 유효성 평가 항목간 상관관계를 비교하고자 Pearson correlation test 를 실시할 수 있다.

체중의 반복측정에 대해서 섭취군 간 비교와 섭취군 내 비교를 RM-ANOVA/Linear mixed model 또는 Friedman’s test 를 이용하여 분석한다. 섭취군 간 차이가 있는 항목에 대하여 사후검정(post-hoc analysis)을 실시한다. 장내 균총 변화 결과에 대해서 반응자/비반응자를 분류하여 분석할 수 있으며, chi-square test 또는 Fisher’s exact test 를 이용하여 분석할 수 있다.

단, 정규성이 심각하게 위배되는 경우 로그변환방법(logarithm

transformation) 등의 변수변환을 실시한 후 검정하거나 비모수검정법인 Wilcoxon signed-rank test, Mann-whitney test 등을 실시할 수 있다.

연속형 자료의 경우 분포에서 비정상적으로 벗어난 유효성 평가지표에 대해 이상치(±1.5SD 이상)로 정의하고, 분석에서 제외할 수 있다.

생활습관(식이섭취, 신체활동, 음주, 흡연 등) 및 동질하지 않은 각 유효성 평가 항목의 기저치 및 인구학적 정보 항목은 covariate 로 보정하여 ANCOVA, ranked ANOVA 를 실시할 수 있다. 유효성 평가에 영향을 미칠 것으로 판단되는 항목(BMI, 쿠퍼만지수 등)에 대하여 층화분석(sub group-analysis)을 실시할 수 있다. 잠재적 혼동요인(나이, 식이섭취, 신체활동, 음주, 흡연, 염증지표, 이상사례, 병용약물, 병력, 이상치 등)에 따라 추가분석을 실시할 수 있다.

<u>안전성 평가 변수</u>

인체적용시험 기간 동안 발생한 모든 이상사례에 대하여 전체와 섭취군 별로 빈도, 퍼센트로 요약한다. 섭취군 간 이상사례의 발생 양상을 chi-square test 또는 Fisher’s exact test 를 이용하여 분석한다.

진단검사의학 검사와 활력징후 결과에 대하여 섭취군 별로 기술통계량을 제시하고, 섭취군 간 비교와 섭취군 내 비교를 실시한다. 섭취군 간 비교의 경우, 변화량(섭취 8 주 후-섭취 전)에 대하여 ANOVA/Linear mixed model 를 이용하여 분석한다. 섭취군 내 비교의 경우, 섭취 전 대비 8 주 시점의 변화에 대하여 paired t test 를 이용하여 분석한다. 또한, 활력징후의 반복측정에 대해서 각 섭취군 간 비교와 섭취군 내 비교를 RM-ANOVA/Linear mixed model 등을 적용하여 분석한다. 단, 정규성검정을 통하여 정규성 만족을 하지 못할 경우, 비모수적 분석방법을 이용한다 (각 섭취군 내 비모수적 분석방법: Wilcoxon signedrank test, 섭취군 간 비모수적 분석방법: Mann-Whitney test).

● 인체적용시험 일정요약

Period	스크리닝 검사	섭취기간		
Visit	스크리닝 방문	1 차 방문	2 차 방문	3 차 방문
Week	-4 이내	0	4	8
서면동의서 1)	●			
인구학적 조사	●			
병력 조사	●			
폐경관련 조사	●			
약물투여력 조사	●	●	●	●
의학적 상태 변화 확인		●		
음주/흡연력 조사	●			●
신체검진	●			●
활력징후	●	●	●	●
신체계측 2)	●			●
선정/제외기준 조사	●	●		
장내 균총 변화		●		●
쿠퍼만지수	●			●
체성분 분석 3)	●		●	●
지질대사지표 4)	●			●
염증관련지표 5)	●			●
혈당관련지표 6)	●			●
단쇄지방산		●		●
EQ-5D	●			●
진단검사의학 검사 7)	●			●
식이섭취조사 및 신체활동조사 8)		●		●
무작위배정		●		
시험용제품 제공		●	●	
이상사례 모니터링			●	●
제품순응도 평가			●	●

* 선정/제외기준을 만족하지 않은 연구대상자에 대해서는 이후 계획한 검사를 실시하지 않을 수 있다.
* 스크리닝 방문을 제외하고 각 방문은 전 3 일, 후 7 일을 허용한다.
  단, 인체적용시험용제품 여유처방을 초과하는 방문은 허용하지 않는다.
* 1 개월은 28 일 기준임

1) 연구대상자가 동의서에 서명을 한 후에 본 연구에 참여할 수 있다.
2) 신체계측: 신장, 허리둘레, 엉덩이둘레, 허리-엉덩이둘레비(WHR)
   단, 신장은 스크리닝 방문 일의 측정값을 연구 종료 시까지 사용한다.
3) 체성분 분석: 체중, 체질량지수(BMI), 체지방량, 체지방률, 제지방량, 복부지방률, 기초대사율
   단, 2 차 방문 시에는 체중만 측정한다.
4) 지질대사지표: Total cholesterol, Triglyceride, LDL-cholesterol, HDL-cholesterol, Non HDL-cholesterol
   * Non-HDL-cholesterol 은 계산공식에 의해 산출한다.
5) 염증관련지표: CRP, ESR
6) 혈당관련지표: glucose, insulin, HOMA-IR, HOMA-β, QUICKI
   * HOMA-IR, HOMA-β, QUICKI 는 계산공식에 의해 산출한다.
7) 진단검사의학 검사 항목
   ▮ 혈액학적 검사: WBC, RBC, Hb, Hct, PLT
   ▮ 혈액생화학적 검사: ALP, gamma-GT, AST, ALT, total bilirubin, total protein, albumin, BUN, creatinine, CK, LD,

uric acid<br>8) 식이섭취조사는 식사기록법에 따르며, 방문 전 3 일(평일 2 일, 주말 1 일)의 식이기록지를 작성한다.<br>신체활동조사는 세계신체활동설문(Global Physical Activity Questionnaire: GPAQ)에 의한다.<br>* 스크리닝 검사는 스크리닝 방문 전 최근 1 개월 이내 진단검사의학 검사 결과로 대체할 수 있다.<br>추가로 필요한 진단검사의학 검사항목은 해당 인체적용시험 스크리닝 방문일에 검사한다.<br>단, 유효성평가항목은 예외임(재검사 필요함).

목 차

● 용어 및 약어의 정의 2
● 인체적용시험계획서 개요 4
● 인체적용시험 일정요약 10
● 제·개정 이력(DOCUMENT HISTORY) 15
1. 인체적용시험의 명칭 및 단계 16
1.1. 인체적용시험의 명칭 16
1.2. 인체적용시험 단계 16
2. 인체적용시험의 실시기관명 및 주소, 시험책임자, 시험담당자 및 인체적용시험용제품 관리자 17
3. 인체적용시험 의뢰자명 및 주소 18
3.1. 인체적용시험 의뢰자명 18
3.2. 인체적용시험 의뢰자 주소 18
4. 인체적용시험수탁기관(CRO) 또는 모니터기관........ 19
4.1. 인체적용시험수탁기관(CRO) 또는 모니터기관명........ 19
4.2. 인체적용시험수탁기관(CRO) 또는 모니터기관 주소........ 19
5. 인체적용시험의 목적 및 배경 20
5.1. 인체적용시험의 목적 20
5.2. 인체적용시험의 배경 20
6. 인체적용시험에 사용되는 제품 22
6.1. 인체적용시험용제품의 개요 22
6.2. 섭취방법 및 일정 22
6.3. 섭취용량 설정 근거 23
6.4. 인체적용시험에 사용하는 제품의 포장........ 23
6.5. 인체적용시험에 사용하는 제품의 라벨링........ 24
6.6. 인체적용시험용제품의 관리 24
6.7. 무작위배정 25
6.8. 순응도 확인 25
7. 대상 연구대상자 26
8. 연구대상자의 선정기준, 제외기준, 목표한 연구대상자의 수 및 그 근거........ 27
8.1. 선정기준 27
8.2. 제외기준 27
8.3. 목표한 연구대상자 수 28
8.4. 연구대상자 수 산정 근거 28
8.5. 인체적용시험 기간 29

9. 인체적용시험 방법 30
9.1. 인체적용시험의 설계 30
9.2. 병용 투여 30
10. 관찰 · 임상검사방법 및 관찰 · 임상검사항목 32
10.1. 관찰 · 임상검사방법 32
10.2. 채혈량 39
10.3. 관찰 · 임상검사항목(방문별) 39
11. 예측 부작용 및 사용상의 주의사항 42
12. 인체적용시험의 중지 및 탈락기준 43
12.1. 인체적용시험 종료 43
12.2. 인체적용시험 중지 및 탈락기준 43
13. 효과 평가기준, 평가방법 및 해석방법(통계 분석 방법) 44
13.1. 유효성평가 44
13.2. 안전성분석 46
14. 이상사례 47
14.1. 이상사례(ADVERSE EVENT, AE) 47
14.2. 약물이상반응(ADVERSE DRUG REACTION, ADR) 47
14.3. 중대한 이상사례(SERIOUS ADVERSE EVENT, SAE) 47
14.4. 시험책임자/담당자에 의한 이상사례의 평가 및 보고 50
15. 인체적용시험 후 연구대상자의 진료 및 치료기준 53
16. 연구대상자의 안전 보호에 관한 대책 54
16.1. 인체적용시험 실시기관 54
16.2. 시험책임자 54
16.3. 기관생명윤리위원회 (IRB) 54
16.4. 인체적용시험 의뢰자와 응급연락 55
16.5. 응급상황 시 인체적용시험용제품 확인 (이중눈가림 해제) 55
16.6. 응급 치료 56
16.7. 이상사례 발생시 조치 56
17. 그 밖에 인체적용시험을 안전하게 과학적으로 실시하기 위하여 필요한 사항 57
17.1. 인체적용시험계획서의 숙지 57
17.2. 연구대상자 모집 방법 57
17.3. 인체적용시험의 동의 57
17.4. 정확한 연구대상자의 선정 57
17.5. 인체적용시험의 진행 점검 57
17.6. 인체적용시험 실시기관의 모니터링 58
17.7. 연구대상자의 비밀유지 58

17.8. 연구대상자를 위한 보험가입 59
17.9. 인체적용시험 자료 및 기록보관 59
18. 참고문헌 60
<부록 1> 쿠퍼만 지수(MODIFIED KUPPERMAN'S INDEX, KI) 62
<부록 2> 삶의 질 평가 설문지(EQ-5D; EUROQOL-5 DIMENSIONS) 63
<부록 3> 식이섭취조사 64
<부록 4> 신체활동조사 69
<부록 5> 헬싱키 선언 71

● 제·개정 이력(Document History)

VERSION NO.	DATE OF ISSUE	SUMMARY OF CHANGES
1.0	2025.03.20	· 제정(최초 version)

1. 인체적용시험의 명칭 및 단계

1.1. 인체적용시험의 명칭

고추발효물(고추장분말)의 장내 균총 및 갱년기 여성건강에 대한 유효성 및 안전성을 평가하기 위한 8 주, 무작위배정, 이중눈가림 인체적용시험

A 8 weeks, randomized, double-blind human trial to evaluate the efficacy and safety of Fermented hot pepper(Gochujang powder)on intestinal microflora and menopausal women's health.

1.2. 인체적용시험 단계

시험단계: 인체적용시험

2. 인체적용시험의 실시기관명 및 주소, 시험책임자, 시험담당자 및 인체적용시험용제품 관리자

<별첨 1> 인체적용시험 실시기관, 시험책임자, 시험담당자 및 인체적용시험용제품 관리자 목록 참조

3. 인체적용시험 의뢰자명 및 주소

3.1. 인체적용시험 의뢰자명

(재)발효미생물산업진흥원

3.2. 인체적용시험 의뢰자 주소

전라북도 순창군 순창읍 민속마을길 61-28

☎ 063-650-2034/ FAX: 063-653-9590

4. 인체적용시험수탁기관(CRO) 또는 모니터기관

4.1. 인체적용시험수탁기관(CRO) 또는 모니터기관명

주식회사 헬스케어크레임스

4.2. 인체적용시험수탁기관(CRO) 또는 모니터기관 주소

전라북도 전주시 덕진구 정여립로 1115 나눔동지타운 506-1 호

☎ 063-212-8284/213-8283/ Fax: 063-276-8287

5. 인체적용시험의 목적 및 배경

5.1. 인체적용시험의 목적

고추발효물(고추장분말)을 8 주간 섭취하였을 때 장내 균총 및 갱년기 여성건강에 대한 유효성 및 안전성을 비교 평가하고자 한다.

5.2. 인체적용시험의 배경

현대사회는 식생활 개선 및 의료기술의 발달로 평균수명이 늘어나 중년기 이후의 삶의 질에 관심이 높아지고 있다. 한국 여성 인구의 중위연령은 2022년 기준으로 45.6세로 2015년 41.4세, 2020년 43.9세에 비해 1.7세높아졌으며 앞으로 10년 후인 2033년에는 50.5세로 5~6세가량 높아질 것으로 예측되며[1], 전체 여성의 3분의 1인 32.5%가 갱년기 여성이 차지하고 있다.

갱년기는 폐경이 되는 45~65세를 전후하여 신체조직이 점차 노화하는 과정에서 생식기에서 비생식기로 이행하는 시기를 말하며[2], 평균 여성의 30% 이상이 겪고 있고 그 비율은 매년 증가하고 있다[3]. 이 시기에는 에스트로젠 저하를 중심으로 하는 내분비계의 변화로 안면홍조(hot flush), 체지방률 증가, 무기질 함량 및 골밀도 감소, 혈중 지질 이상, 우울감 등 다양한 증상이 나타난다[4,5]. 폐경 전, 후에 발생하는 이러한 여러 문제에 대해 양방의 임상적 관점은 호르몬 대체 요법에 집중되어 있다[3,6]. 그러나 호르몬 대체 요법은 정맥혈전색전증과 뇌중풍의 빈도를 증가시키며, 장기 투여시 유방암 발병위험을 증가시킬 뿐 아니라 관상동맥질환, 불규칙 질출혈, 체중 증가, 소화 장애 등의 부작용이 나타날 수 있다[7,8]. 이에 반해 아마씨, 칡, 석류, 오미자 등 우리 주변에서 접할 수 있는 식물자원 중에서도 여성갱년기에 효과가 있다는 연구가 보고되고 있을 정도로 건강기능식품의 개발이 꾸준히 진행되고 있다[9].

장내 세균은 인체의 장내에서 상호공생 또는 길항 관계를 유지하며 사람의 나이와 식이 등에 대해 크게 영향을 받는다. 인간이 노쇄하면 소화흡수, 장운동, 면역기능 저하 등에 의해 유해균의 번식이 증가할 수 있다. 따라서 이런 유해균의 생육을 억제시킬 수 있는 소재에 대한 연구가 미비한 실정이므로 장내 균총에 대한 식품 소재 연구가 필요하다[10].

고추(*Capsicum annuum* L.)는 매운맛을 내는 capsaicin 과 dihydrocapsaicin 등의 캡사이시노이드(capsaicinoid)라는 페놀산(phenolic acid) 성분이 알려져 있다. 특히, capsaicin 은 우수한 생리활성효과가 밝혀지면서 류마티스 관절염, 수술 후 통증, 만성 신경병증 및 근골격 통증치료의 임상시험 파이프라인에 있으며, 이외에도 항산화, 항염증, 항암, 항비만, 심장 보호 및 대사조절에 대한 약리학적 효과와 작용기전이 광범위하게 연구되고 있다 [11].

고추장은 간장 및 된장과 더불어 주로 조리를 목적으로 예전부터 널리 애용되어 온 발효식품 중의 하나로 그 맛은 전분질의 가수분해로 생성되는 당류와 단백질의 가수분해로부터 생성되는 아미노산에 기인하는 것으로 알려져 있다. 또한 vitamin B1, B2, C 및 folic acid 등 다량 함유되어 있어 비타민의 주요 공급원이기도 하다. 또한 미생물의 대사 및 발효작용으로 생성되는 유기산, 핵산, 알코올 및 색소 등 미량 성분이 맛, 향 및 색 등에 조화를 이룬 발효 식품이다[12].

이에 따라 본 연구를 통해 인체에서 고추발효물(고추장분말)의 장내 균총 및 갱년기 여성건강에 대한 효과를 과학적으로 평가하고자 한다.

6. 인체적용시험에 사용되는 제품

6.1. 인체적용시험용제품의 개요

(1) 고추발효물(고추장분말)

인체적용시험용제품명:

- 고추발효물 TCG25-26(유용미생물 고함량 고추장분말)
- 고추발효물 TMG25-2(유용미생물 저함량 고추장분말)
- 고추발효물 TFG25-2(시판용 고추장분말)

성상 및 제형: 고유의 향미가 있는 진한 갈색의 분말 / 환

사용기간: 24 개월

저장방법: 실온보관

성분	고추발효물 TCG25-26 (유용미생물 고함량 고추장분말)군		고추발효물 TMG25-2 (유용미생물 저함량 고추장분말)군		고추발효물 TFG25-2 (시판용 고추장분말)군	
	함량(g/환)	배합비(%)	함량(g/환)	배합비(%)	함량(g/환)	배합비(%)
고추장분말	3.15	75	3.15	75	3.15	75
찹쌀가루	0.21	5	0.21	5	0.21	5
결정셀룰로오스	0.84	20	0.84	20	0.84	20
합계	4.2	100	4.2	100	4.2	100

✓ 고추발효물 TCG25-26(유용미생물 고함량 고추장분말), 고추발효물 TMG25-2(유용미생물 저함량 고추장분말)은 전통방식으로 제조 및 관리 공정을 통해 만들어진 재래식 고추장으로 자연발효과정에서 생성된 미생물 군집분석을 통해 미생물 분포도를 조사하였으며, 조사를 통해 유용미생물{식약처 고시 식품 소재 사용 가능 미생물(GRAS)} 함량 차이로 저함량(유용미생물 함량: 19.55%)/고함량(유용미생물 함량: 72.60%)을 구분하여 설정하였습니다. 고추발효물 TFG25-2(시판용 고추장분말)은 시중에 판매되고 있는 종균 처리를 통한 인위적 발효과정을 거쳐 제조 및 관리 공정을 통해 만들어진 공장식 고추장으로서, 재래식 고추장군과 비교하기 위해 대조군으로 설정하였습니다.

6.2. 섭취방법 및 일정

1 차 방문 시 연구대상자 번호가 부여되며 다음 세 군 중 하나에 무작위배정되어 8 주 동안 인체적용시험용제품을 1 일 2 회 경구 섭취한다.

- 고추발효물 TCG25-26(유용미생물 고함량 고추장분말)군: 1 일 2 회, 1 회 3 포, 아침/저녁 식후 경구섭취(25.2 g/day, 고추장분말로써 19 g/day)
- 고추발효물 TMG25-2(유용미생물 저함량 고추장분말)군: 1 일 2 회, 1 회

3 포, 아침/저녁 식후 경구섭취(25.2 g/day, 고추장분말로써 19 g/day)

- 고추발효물 TFG25-2(시판용 고추장분말)군: 1 일 2 회, 1 회 3 포, 아침/저녁 식후 경구섭취(25.2 g/day, 고추장분말로써 19 g/day)

6.3. 섭취용량 설정 근거

- 원재료 고추는 「식품공전」 식품원재료로써, '식품공전 상 식품에 사용할 수 있는 원료의 목록에 등재(고유번호: A 가 017100)' 되어 있으며, 식품원재료 DB 검색결과 '고추' 의 잎, 열매가 식용 가능한 것으로 등재되어 있다.
- 고추장은 「식품공전」에 '두류 또는 곡류 등을 주원료로 하여 누룩균 등을 배양한 후 고춧가루(6% 이상), 식염 등을 가하여 발효 숙성하거나 숙성 후 고춧가루(6% 이상), 식염 등을 가한 것을 말한다' 로 명시되어 있다.
- '05 년 식이노출량 평가를 위한 식품별 섭취량 분포 - 식품별 1 인 1 일당 섭취량(한국보건산업진흥원)에서 평균 섭취량은 4.376 g, 극단 섭취량은 1000 g 로 조사되었으며, '14 년 국민건강영양조사를 이용한 식품별 1 인 1 일 섭취량 분포 - 섭취자(한국보건산업진흥원)에서 평균 섭취량은 10.75 g, 극단 섭취량은 3686 g 으로 조사되었으며, 국민영양통계에서 2019 년도 다빈도식품 17 위에 해당하며, 식품별 섭취량 평균은 6.4 g 으로 조사되었다.
- 「식품등의 표시기준(식품의약품안전처 고시 2021-7 호, 2021.2.5.)」에서 고추장 1 회 섭취 참고량은 10 g 으로 나와있다.
- 유사원료로 진행된 1 건의 in vivo 시험에서 고추장 2 g/kg 을 2 주간 경구투여시킨 결과, 특이한 이상사례에 대한 내용은 언급되지 않았고, 이를 인체섭취용량으로 환산 시 약 19.2 g/일에 해당한다 [13].
- 따라서 건강기능식품 기준 및 규격, 판매현황 및 섭취경험, 동물시험 결과 등을 토대로 본 연구에서는 고추발효물(고추장분말)로써 19 g 을 1 일 섭취량으로 설정하고자 한다.

6.4. 인체적용시험에 사용하는 제품의 포장

인체적용시험용제품은 제조 후 인체적용시험기관 제품관리자에게 공급한다. 각각의 인체적용시험용제품은 외형 및 성상이 동일하여 육안으로는 차이가 관찰되지 않아야 하며, 중량 차이도 크지 않아야 한다. 인체적용시험용제품은 28 일 간격으로 제공하도록 하고, 7 일분의 여유분을 합하여 1 회에 35 일분을 포장하여 연구대상자에게 제공한다.

6.5. 인체적용시험에 사용하는 제품의 라벨링

인체적용시험용제품의 라벨에는 다음의 정보가 포함되어야 한다.

- "인체적용시험용"이라는 표시
- 연구계획서 번호
- 제품의 코드명 또는 주성분명의 일반명
- 연구대상자 코드
- 방문 번호
- 유효기간
- 제조번호 또는 배치번호
- 저장방법
- 의뢰자 명칭 (위탁제조의 경우에는 제조원 포함)
- "인체적용시험 외의 목적으로 사용할 수 없음"이라는 표시
- "어린이의 손이 닿지 않는 곳에 보관"이라는 표시

6.6. 인체적용시험용제품의 관리

인체적용시험책임자 또는 관리자는 시험기관에서 수령한 모든 인체적용시험용제품을 인체적용시험 기간 중 보관하고 수량을 관리할 책임이 있다. 연구대상자에 대한 인체적용시험용제품의 배분 및 회수는 인체적용시험용제품 수량 관리 양식에 기록되어야 한다. 연구대상자 또는 법적으로 허용되는 대리인은 본래의 용기에 인체적용시험용제품의 유·무에 상관없이 모든 용기를 반납하도록 하여야 한다. 연구대상자로부터 회수된 인체적용시험용제품은 보관되어 인체적용시험 의뢰자의 지시사항에 따라 처리한다.

인체적용시험용제품의 관리는 인체적용시험계획서와 용기 라벨에 따라 엄격하게 행해져야 하며, 적합한 환경조건에서 접근이 제한된 장소나 시건장치가 부착된 캐비닛 혹은 냉장고에 보관되어야 한다. 사용되지 않은 인체적용시험용제품과 연구대상자에게서 회수된 인체적용시험용제품은 시험의뢰자나 모니터링 방문 시 시험의뢰자의 모니터에 의해 확인되어야 하며, 회수된 모든 인체적용시험용제품들은 인체적용시험용제품 회수 양식에 기록되어야 한다.

인체적용시험용제품은 인체적용시험책임자/담당자 또는 제품관리자의 감독하에 배분되어야 한다. 인체적용시험용제품은 연구에 참여하는 연구대상자들에게만 공급되며, 회수된 인체적용시험용제품은 다시 사용할 수 없으며,

동일한 연구대상자라도 사용될 수 없다. 인체적용시험용제품은 다른 연구대상자에게 사용하기 위해 다시 라벨링 되거나 재배분 될 수 없다. 시험책임자는 인체적용시험용제품을 시험의뢰자와 사전 동의하지 않은 장소에서 배분하거나 보관하지 않을 것임에 동의해야 한다.

6.7. 무작위배정

무작위배정은 이중눈가림 단계에서 사용되어 연구대상자의 시험군 배정 시 편견을 피하고, 각 시험군마다 알려지거나 미지수인 연구대상자의 특징(즉, 인구학적 특성과 기초평가일 특성치)이 골고루 균형을 이루며, 시험군 간의 통계적 비교의 유효성을 증가시킨다. 무작위배정은 일정한 크기(예를 들어 3 명, 6 명, 9 명 등)의 블록을 만들고 각 블록 내에서 일정한 배정비(1:1:1)에 따라 배정하는 방법인 블록 무작위배정 방법을 사용한다. 이중눈가림 섭취는 자료수집과 시험 종료 시점의 평가에서 잠재적인 편견을 줄이기 위해 사용된다. 시험 기간 동안은 연구자나 연구대상자가 연구대상자의 무작위배정 정보를 알 수 없다. 이것이 알려지면 인체적용시험 이중눈가림이 유지될 수 없기 때문이다. 무작위배정 코드는 의뢰자가 유지보관하는 것을 원칙으로 한다. 일반적인 경우 모든 연구대상자들이 시험을 완료하고 자료가 locking 되기 전에는 이중눈가림을 해제해서는 안된다. 다만, 연구대상자의 섭취상황을 알아야 하는 응급상황일 경우에는 이중눈가림을 해제할 수 있다. 이 경우 인체적용시험책임자는 의뢰자에게 가능한 한 신속하게 연락해야 한다. 이중눈가림을 해제하게 된 날짜, 시간, 이유를 증례기록서와 근거문서의 적절한 곳에 문서화해야 한다. 의뢰자로부터 받은 이중눈가림 코드 해제 확인 서류 복사본은 근거문서에 보관되어야 한다.

6.8. 순응도 확인

인체적용시험용제품의 섭취상황에 대하여 2 차, 3 차 방문시 연구대상자에게 섭취 후 남은 인체적용시험용제품을 지참하고 방문하도록 지도하여 연구대상자가 지참하고 온 인체적용시험용제품의 잔여량을 반납받고 순응도를 확인한다. 연구대상자가 지참하고 온 잔여량은 반드시 약국에 반납하여 수불장부를 기록하여야 한다.

7. 대상 연구대상자

스크리닝 검사 당시 연령이 만 45 세 이상, 70 세 이하인 성인 여성으로서, 폐경(12 개월 이상 무월경 상태)이며 쿠퍼만지수(Modified Kupperman's index)점수가 15 점 이상이고 스크리닝 검사 당시 BMI 가 23 kg/m^2 이상인 자

8. 연구대상자의 선정기준, 제외기준, 목표한 연구대상자의 수 및 그 근거

8.1. 선정기준

모든 연구대상자는 다음 기준을 만족하여야 인체적용시험에 참여할 수 있다.

(1) 스크리닝 검사 당시 연령이 만 45 세 이상, 70 세 이하인 성인 여성

(2) 폐경(12 개월 이상 무월경 상태)인 자

(3) 쿠퍼만지수(Modified Kupperman's index)점수가 15 점 이상인 자

(4) 스크리닝 검사 당시 BMI 가 23 kg/m^2 이상인 자

(5) 본 인체적용시험에 대한 자세한 설명을 듣고 완전히 이해한 후, 자의로 참여를 결정하고 주의사항을 준수하기로 서면 동의한 자

8.2. 제외기준

연구대상자들이 다음 기준 중 하나라도 해당될 경우 인체적용시험의 참여에서 배제된다.

(1) 호르몬 대체요법을 시행하고 있거나, 최근 6 개월 이내에 호르몬 대체요법을 시행한 경험이 있는 자

(2) 자궁내막증식증, 자궁암, 자궁내막암, 유방암 또는 유방질환의증, 성호르몬 관련암 등이 있거나 과거력이 있는 자

(3) 난소 기능에 영향을 줄 수 있는 화학요법이나 방사선요법을 받거나 받은 경험이 있는 자

(4) 양측난소절제술의 과거력이 있는 자

(5) 치료를 요하는 임상적으로 유의한 급성 또는 만성 심뇌혈관계, 내분비계, 면역계, 호흡기계, 간담도계, 신장 및 비뇨기계, 신경정신계, 근골격계, 염증성 및 혈액·종양성, 위장관계 질환 등이 있는 자

(6) 스크리닝 검사 전 1 개월 이내에 장내 균총 및 갱년기 여성건강과 관련된 의약품 및 건강기능식품을 복용한 자(병용금지약물 참고)

(7) 고추, 고추장 성분에 유의한 과민반응의 병력이 있는 자

(8) 스크리닝 검사 전 3 개월 이내에 항정신병 약물치료를 받은 경험이 있는 자

(9) 알코올 중독 또는 약물 남용이 있거나 의심되는 자

(10) 스크리닝 검사 전 3 개월 이내에 타 인체적용시험에 참여한 자

(11) 진단검사의학 검사에서 다음에 해당하는 결과를 보이는 자

☞ AST, ALT > 참고범위 상한치의 3 배

☞ Serum Creatinine > 2.0 mg/dL

(12) 진단검사의학 검사결과를 비롯한 기타 사유로 인하여 시험책임자가 연구 참여에 부적합하다고 판단한 자

8.3. 목표한 연구대상자 수

본 인체적용시험에는 총 60 명(각 군당 20 명)의 연구대상자가 무작위배정되어 시험을 실시할 것이다. 연구대상자 수 60 명은 20%의 탈락률을 고려한 것이며, 실제로 48 명(각 섭취군 당 16 명)의 연구대상자가 본 인체적용시험을 중대한 위반 없이 완료할 것으로 예상된다.

8.4. 연구대상자 수 산정 근거

본 인체적용시험의 목적은 8 주간 고추발효물 섭취에 의한 장내 균총 및 갱년기 여성건강 개선 효과가 대조제품 섭취와 비교하여 차이가 있음을 증명하고자 함이다.

본 인체적용시험에서는 고추발효물 TCG25-26(유용미생물 고함량 고추장분말)과 고추발효물 TMG25-2(유용미생물 저함량 고추장분말)의 우위성을 검증하기 위한 연구대상자 수를 산출하기 위하여, 고추발효물 TCG25-26(유용미생물 고함량 고추장분말)의 8 주 섭취 후 쿠퍼만지수의 변화량은 -7.8 점, 고추발효물 TMG25-2(유용미생물 저함량 고추장분말)은 -6.8 점, 고추발효물 TFG25-2(시판용 고추장분말)은 -3.7 점으로 가정하였다. 위의 가정에 의해 본 인체적용시험의 연구대상자 수를 다음과 같이 산출하였다 [14).

(1) 5% 유의수준에서 제 2 종 오류는 0.2 로 하여 80% 검정력을 유지한다 (α = 0.05, β = 0.2, 검정력 = 80%)

(2) 기초 평가로부터 시험 종료시점에 이르기까지 고추발효물 TCG25-26(유용미생물 고함량 고추장분말)의 쿠퍼만지수의 변화량은 -7.8 점, 고추발효물 TMG25-2(유용미생물 저함량 고추장분말)의 변화량은 -6.8 점, 고추발효물 TFG25-2(시판용 고추장분말)의 변화량은 -3.7 점으로 가정하였다.

(3) 각 시험군의 표준편차(σ)는 3.9 로 가정하였다.

본 연구에서는 세 군의 변화량을 비교하여 고추발효물 TCG25-26(유용미생물 고함량 고추장분말)과 고추발효물 TMG25-2(유용미생물 저함량 고추장분말)이 우위함을 가정하고 그에 대한 시험예수를 산정한다.

이를 검정하기 위한 가설은 다음과 같다.

H_0: $\mu 1 = \mu 2 = \cdots = \mu k$ versus Ha : $\mu i \neq \mu j$ for some $1 \leq i < j \leq k$

위의 (1)-(3)을 가정했을 때 인체적용시험에 필요한 연구대상자 수는 다음과 같다.

$$\Delta = \frac{1}{\delta^2}\sum_{j=1}^{k}(\mu_j - \bar{\mu})^2 = 0.6009$$

각 군에서 필요한 연구대상자 수 n은 $n = \frac{\lambda}{\Delta}$ = 16이다

위의 계산으로부터 필요한 연구대상자 수를 산출하고 중도 탈락률 20%를 고려하여 다음 식에 의하여 인체적용시험에 필요한 최종 연구대상자 수를 산출하였다.

$$\mathrm{n} = \frac{n_i}{1-r} = 20$$

각 군당 20 명의 연구대상자 수가 산출되었으며, 각 시험군의 배정비를 1:1:1 로 하여, 고추발효물 TCG25-26(유용미생물 고함량 고추장분말)군 20 명, 고추발효물 TMG25-2(유용미생물 저함량 고추장분말)군 20 명, 고추발효물 TFG25-2(시판용 고추장분말)군 20 명이며, 세 시험군을 통틀어 요구되는 총 연구대상자 수는 60 명이다.

8.5. 인체적용시험 기간

IRB 승인일로부터 12개월

9. 인체적용시험 방법

9.1. 인체적용시험의 설계

시험자는 서면동의서를 작성한 자원자에 한하여 스크리닝 검사를 시행하여 선정기준에 부합하는 연구대상자를 선정한다. 연구대상자는 스크리닝 방문으로부터 4 주 이내에 1 차 방문하여 연구대상자 선정/제외기준 적합성을 재검토 받은 후 인체적용시험에 등록되며, 고추발효물 TCG25-26(유용미생물 고함량 고추장분말)군, 고추발효물 TMG25-2(유용미생물 저함량 고추장분말)군, 고추발효물 TFG25-2(시판용 고추장분말)군에 무작위배정 되어 1 차 방문일까지 기초평가(Baseline)를 완료한다. 이후 시험용제품을 하루 1 번씩 8 주간 매일 섭취하면서 4 주마다 수행기관에 방문하여 유효성평가, 활력징후, 약물투여력 및 의학적상태변화 조사, 이상사례 조사 등을 수행한다.

시험자는 인체적용시험용제품 최종 섭취 후 혹은 조기종료 방문 후 필요에 따라 연구대상자 추적 관찰을 실시할 수 있다.

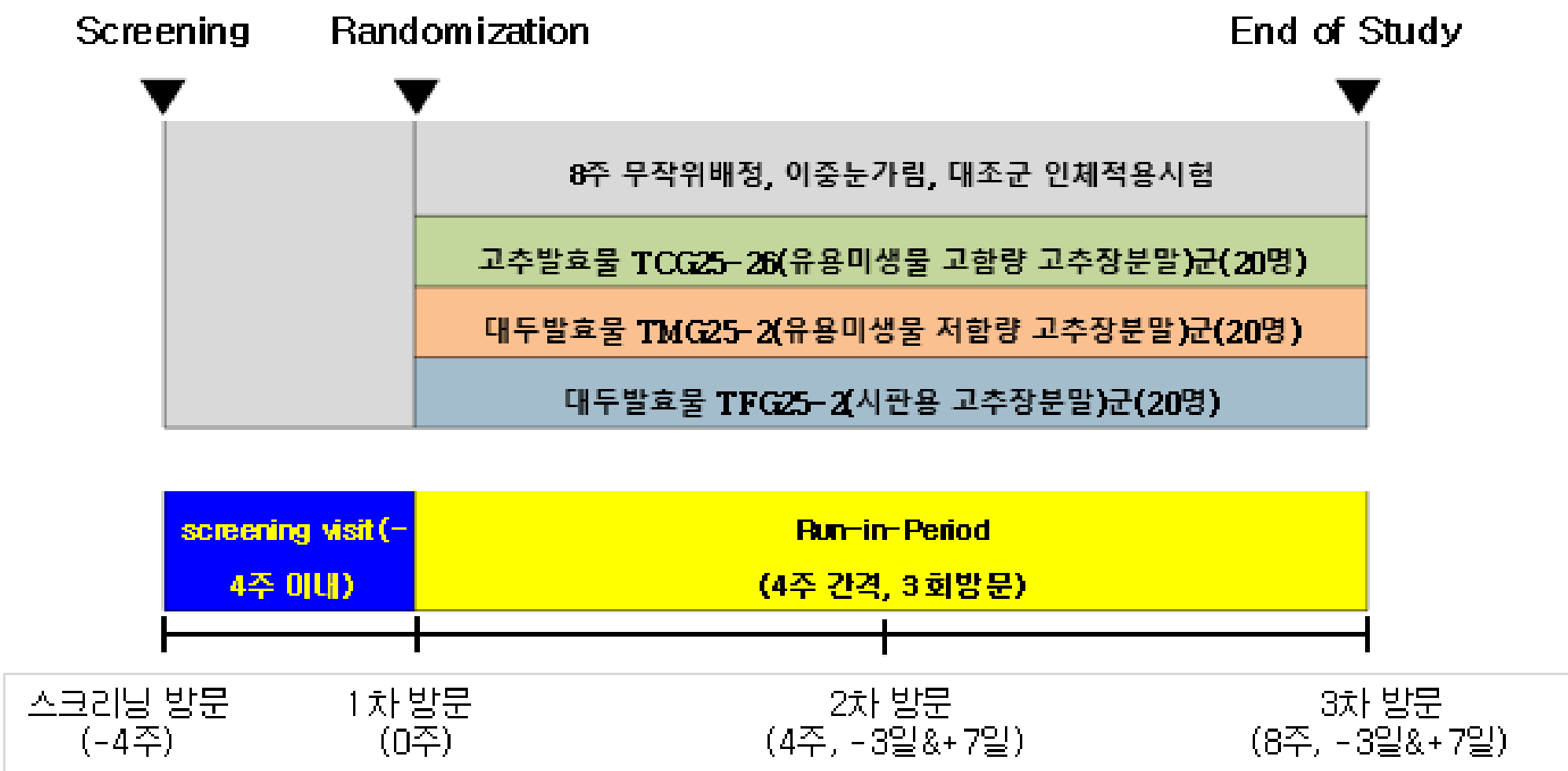

9.2. 병용 투여

(1) 병용가능 약물

- 연구대상자가 시험에 참가하기 1 개월 이전부터 복용하고 있던 약물 중 본 시험의 결과해석에 영향을 미치지 않을 것으로 판단되는 병용약물 (제품)은 시험책임자의 판단 하에 허용한다.
- 기타 질환 또는 이상사례의 치료를 목적으로 일과성으로 사용되는 약제는 담당의사와 상의를 통하여 병용 투여하기로 한다.

- 시험책임자의 판단 없이 임의로 투약한 약물이 시험의 유효성 평가에 영향을 줄 수 있다고 예상되는 경우 이 연구대상자는 탈락하게 된다. 투여한 모든 제품 및 투여사유는 반드시 증례기록서에 기재하고 시험책임자가 서명한다.

(2) 병용금지 약물

다음에 열거하는 건강기능식품 및 약물은 시험기간 동안 병용투여를 금지한다:

- 항생제(페니실린, 세팔로스포린, 모노박탐, 카바페넴, 아미노글리코시드, 퀴놀론 계열) - 연구책임자 판단하에 일시적 섭취는 허용
- 호르몬 제제(에스트로겐, 프로게스토겐, 티볼론 계열)
- 갱년기 여성건강 관련 건강기능식품{홍삼, 회화나무열매추출물, 피크노제놀-프랑스해안송껍질추출물, 백수오 등 복합추출물, 석류추출물농축액, 석류농축액, 석류추출물, 오미자추출물, 루바브뿌리추출물, *Lactobacillus acidophilus*(락토바실러스 아시도필루스) YT1(HU038), 대두추출물등 복합물(메노세라), MS-10 엉겅퀴등복합추출물, 루바브뿌리추출물(ERr731®), 발아발효콩추출물, 루바브뿌리추출물, 단삼주정추출분말 등}
- 장건강 관련 건강기능식품{알로에 전잎, 구아검/구아검가수분해물, 글루코만난(곤약, 곤약만난), 난소화성말토덱스트린, 대두식이섬유, 목이버섯식이섬유, 밀식이섬유, 보리식이섬유, 아라비아검(아카시아검), 이눌린/치커리 추출물, 차전자피식이섬유, 폴리덱스트로스, 알로에 겔, 프락토올리고당, 프로바이오틱스, 라피노스, 분말한천, 이소말토올리고당, 대두올리고당, 커피만노올리고당분말, 락추로스 파우더, 자일로올리고당, 밀전분유래난소화성말토덱스트린, 무화과페이스트, 쇠비름주정추출분말(KDC16-2), 갈락토올리고당 분말(Bimuno®GOS powder), 자일로올리고당(xylooligosaccharide) 분말, *Bacillus coagulans* Unique(바실러스 코아귤런스 유니크) IS-2 프로바이오틱스 등}
- 고추 및 캡사이신을 함유한 한약 및 건강기능식품
- 기타 시험책임자가 인정하지 않는 제제나 제품

연구대상자의 다른 의학적 증상 치료를 담당하는 의사의 판단에 따라 인체적용시험 기간 중 연구대상자의 치료를 위해 병용금기 약물의 사용이 필요한 경우에 해당 연구대상자는 즉시 인체적용시험이 중단되어야 하며, 증례기록서의 마지막 페이지에 관련내용을 자세히 기록해야 한다.

10. 관찰 · 임상검사방법 및 관찰 · 임상검사항목

10.1. 관찰 · 임상검사방법

(1) 연구대상자 동의서(Informed Consent Document, ICD)

연구자는 인체적용시험 전반에 걸쳐 연구대상자가 가질 수 있는 질문에 대해 답변하고 연구대상자의 인체적용시험 참여 지속 의사에 중요할 수 있는 새로운 정보가 입수되는 대로 적절한 시한 내에 이를 공유하는 것을 포함하여 연구대상자가 인체적용시험 참여로 인한 위험 및 이익을 이해하도록 확인하는 책임이 있다. ICD 가 새로운 정보에 따라 갱신되는 경우 연구자는 갱신된 ICD 를 사용하여 연구대상자의 인체적용시험 참여 지속 의사를 다시 한번 확인할 것이다. ICD 는 연구대상자가 인체적용시험에 참여하기 이전에 쉬운 용어를 사용하여 인체적용시험 참여에 따르는 위험 및 이익을 연구대상자에게 설명하기 위해 사용될 것이며, 연구대상자가 인체적용시험 참여에 따르는 위험 및 이익에 대해 만족할 만큼 이해하고 인체적용시험에 참여하고자 하는지 문서화하기 위해 사용될 것이다.

연구자는 각 연구대상자가 제출한 연구대상자 동의서를 확인할 책임이 있다. 여기에는 모든 인체적용시험계획서 상의 절차수행 및 인체적용시험용제품의 복용이전에 적절한 서명 및 날짜가 기재되어야 하는 점이 포함된다.

(2) 스크리닝 번호 및 연구대상자 번호 부여

인체적용시험에 참여하고자 서면 동의한 자원자에게 스크리닝 번호를 부여한다. 스크리닝 번호는 1-S+숫자 세자리로 구성되며(1-S001~), 동의서를 받은 순서대로 부여한다. 연구대상자 번호 부여방법은 스크리닝 검사를 시행하여 본 인체적용시험의 연구대상자로서 적합하다고 판단되는 자를 선정하고, 1 차 방문 시 최종통과 되면 통과된 순서대로 무작위배정하여 연구대상자 번호를 부여한다. 연구대상자 번호는 1-R+숫자 두자리로 구성되며(1-R01~) 연구대상자 번호 숫자부분의 의미는 다음과 같다. 숫자는 참여한 연구대상자의 순서를 나타내며 01~60 까지 나타낼 수 있다. 각 연구대상자에게 부여된 연구대상자 번호는 인체적용시험이 끝날 때까지 연구대상자를 인식하는 연구대상자식별코드(subject identification code)로 사용된다.

(3) 인구학적 조사

연구대상자의 신분증을 통하여 인구학적 정보(성명, 성별, 연령)를 확인한다. 연령은 만 나이로 기록하며, 만약 연구대상자의 실제 상황과 신분증의 기록이 다를 경우(예: 출생신고를 잘못하여 연령에 차이를 보이는 경우) 연구대상자의 실제 정보를 기록하고 그 내용을 근거문서에 기록한다.

(4) 음주력 및 흡연력 조사

연구대상자의 음주력은 음주여부, 음주량을 조사하며, 음주량은 정수로 기재한다. 흡연력은 흡연여부, 흡연량을 조사한다.

계산식	
음주량	알코올 10 g = 12.5 mL
	[알코올 섭취량(mL) × 알코올 도수(%) × 0.8]/100

(5) 병력 및 약물 투여력 조사

연구대상자가 동의서에 서명한 시점에서, 6 개월 이내에 가지고 있는 과거 병력, 악성종양의 경우 5 년 이내의 병력을 확인하고, 증례기록서의 해당 페이지에 기록한다. 인체적용시험기간 동안 질환이 처음으로 발생하거나 병발질환이 시험기간 동안 악화된 경우에는 이상사례로 간주하고 기록한다. 또한 모든 병용약물의 제품명 및 성분명, 용량, 용법, 투여기간 등을 상세히 기록한다.

(6) 신체검진

신체검진은 문진, 시진, 청진, 타진, 촉진 등을 수행하며, 연구대상자가 오한을 느끼지 않도록 수행장소가 적절한 온도를 유지하도록 한다.

(7) 활력징후

활력징후는 좌위 혈압, 맥박수 항목을 측정한다. 혈압은 연구대상자의 팔이 심장의 높이에 위치하도록 하여 측정하며, 단위는 mmHg 이고 정수로 표기한다. 급격한 체위변동 없이 5 분 이상 좌위 자세를 유지한 상태에서 혈압, 맥박수를 측정한다.
활력징후 측정시기가 채혈시기와 일치할 경우, 가급적 활력징후를 먼저 측정한 후 채혈을 하도록 한다. 불가피하게 채혈 후 활력징후를 측정할 경우, 가급적 5분 이상 시간 간격을 두고 활력징후를 측정하도록 한다.

(8) 폐경관련 조사

폐경관련 조사 항목은 초경 나이, 폐경 나이, 폐경일, 폐경 기간, 출산 횟수에 대해 조사한다. 폐경일 및 폐경 나이는 무월경이 12 개월 이상이 되는 시점을 기재하며, 폐경 기간은 폐경일 이후 조사 시점까지로 계산하며 소수점은 버림으로 처리하여 정수로 표기한다.

(9) 신체계측

신체계측 항목은 방문 시 동일한 기계를 사용하여 표준화된 방법으로 측정한다. 신체계측 항목은 신장, 허리둘레, 엉덩이둘레, 허리-엉덩이둘레비(WHR)를 측정하며, 측정방법은 아래와 같다.

① 신장

신장은 단위를 cm 로 하며, 스크리닝 방문 시의 측정값으로 인체적용시험 종료 시까지 기재하도록 하며, 소수점 첫째 자리까지 표기한다.

② 허리둘레

허리둘레는 단위를 cm 로 하며, 줄자를 이용하여 각각 소수점 첫째자리까지 3 회 측정하며 측정치의 평균을 소수점 둘째 자리에서 반올림하여 소수점 첫째자리까지 표기한다. 허리둘레는 배꼽을 기준으로 측정하며, 연구대상자가 측정 중에 자연스럽게 호흡하도록 하며 호기 마지막에 측정한다.

③ 엉덩이둘레

엉덩이둘레는 단위를 cm 로 하며, 줄자를 이용하여 각각 소수점 첫째자리까지 3 회 측정하며 측정치의 평균을 소수점 둘째 자리에서 반올림하여 소수점 첫째자리까지 표기한다. 엉덩이둘레는 측면에서 보아 엉덩이 뒷부분 중 가장 돌출된 부분을 수평으로 측정한다.

④ 허리-엉덩이둘레비(WHR)

허리-엉덩이 둘레비(waist hip ratio, WHR)는 허리둘레를 엉덩이둘레로 나눈 값으로 소수점 셋째 자리에서 반올림하여 소수점 둘째 자리까지 표기한다.

(10) 장내 균총 변화

장내 균총 분석을 위해, 연구대상자는 스크리닝, 2 차 방문 시 제공되는 채변키트에 1 g 이상의 분변을 담아 냉동 보관한다. 보관된 검체는 이니셜 및 코드번호로 관리되며, 운송상자에 동봉하여 외부위탁기관에 이송한 후 분석을 수행한다. 채취된 분변은 분석 후 폐기한다. 검사항목은 다음과 같다.

검사항목	
장내 균총 변화	유익균, 유해균, 기타균의 비율(%)

(11) 쿠퍼만지수(Modified Kupperman's index, KI)

대표적인 갱년기 증상을 점수화하여 갱년기 장애의 정도 및 특징을 파악하는 설문도구 로서 안면홍조, 감각이상(손발 저림), 불면증, 신경질, 우울증, 현기증, 피로감, 관절통이나 근육통, 두통, 가슴두근거림 그리고 의주감(피부에 개미가 기는 느낌, 근질근질함)의 11 가지 갱년기 증상에 대해 평가한다. 각 증상에 대해서 0 점(없음)에서 3 점(심함)의 4 점 척도로 점수화하고, 안면홍조는 4 점의 가중치, 감각이상(손발 저림), 불면증, 신경질은 2 점의 가중치, 나머지는 1 점의 가중치를 두어 총 51 점으로 이루어진다. 15~20 점일 때 경

증, 21~35 점일 때 중증도, 35 점 이상일 때는 중증으로 분류한다[15-17]. 또한 갱년기 증상과 관련하여 질 건조감 또는 질 분비물 감소에 대해서도 0 점(없음)에서 3 점(심함)의 4 점 척도로 점수화하고 1 점의 가중치를 두어 평가한다.

<부록 1> 쿠퍼만지수(Modified Kupperman's index)

(12) 체성분 분석

체중(weight, kg), 체질량지수(body mass index, kg/m^2), 체지방량(body fat mass, kg), 체지방률(percent body fat, %), 제지방량(fat free mass, kg), 복부지방률(waist-hip ratio), 기초대사량(basal metabolic rate, kcal) 측정은 Inbody 970 을 이용하여 측정한다. 최소한의 옷을 입은 상태에서 측정한다.

① 체중: 체중은 단위를 kg 으로 하며, 소수점 첫째 자리까지 표기한다(단, 소수점 둘째 자리까지 나오는 기계일 경우, 둘째 자리에서 반올림하여 첫째 자리까지 표기한다).

② 체질량지수(BMI): 체질량지수(body mass index, kg/m^2)는 체중(단위: kg)을 신장(단위: m)의 제곱으로 나눈 값이며, 소수점 둘째 자리에서 반올림하여 소수점 첫째 자리까지 표기한다.

(13) 지질대사지표

지질대사지표는 12 시간 이상 공복상태에서 채혈하여 측정하는 것을 원칙으로 하고, 채취된 혈액은 분석 후 폐기한다. 검사 항목은 다음과 같다.

✓ Non-HDL-cholesterol 은 계산공식에 의해 산출하며 소수점 첫째 자리까지 표기한다.

검사항목	
지질대사지표	Total cholesterol, Triglyceride, LDL-cholesterol, HDL-cholesterol, Non HDL-cholesterol
**계산식**	
Non-HDL-cholesterol	총콜레스테롤 - (HDL-C)

(14) 염증관련지표

CRP, ESR 은 12 시간 이상 공복상태에서 채혈하여 측정하는 것을 원칙으로 하며, 채취된 혈액은 분석 후 폐기한다.

검사항목	
염증관련지표	CRP, ESR

(15) 혈당관련지표

glucose, insulin 은 12 시간 이상 공복상태에서 채혈하여 측정하는 것을 원칙으로 하며, 채취된 혈액은 분석 후 폐기한다. 검사항목은 다음과 같다.

HOMA-IR 은 계산공식에 의해 산출하며, 소수점 둘째 자리에서 반올림하여 소수점 첫째 자리까지 표기한다. HOMA-β는 계산공식에 의해 산출하며, 소수점 첫째 자리에서 반올림하여 정수로 표기한다. QUICKI 는 계산공식에 의해 산출하며, 소수점 넷째 자리에서 반올림하여 소수점 셋째 자리까지 표기한다.

✓HOMA-IR, HOMA-β, QUICKI 는 연구종료 후 계산식으로 일괄 산출한다.

검사항목	
혈당관련지표	glucose, insulin, HOMA-IR, HOMA-β, QUICKI
**계산식**	
HOMA-IR	[공복 시 혈당(mg/dL) x 공복 시 인슐린 (μU/mL)] / 405
HOMA-β	306 x 공복 시 인슐린(μU/mL) / {공복 시 혈당(mg/dL)-63}
QUICKI	1 / [log(공복 시 인슐린, μU/mL) + log(공복 시 혈당, mg/dL)]

(16) 단쇄지방산

단쇄지방산 분석을 위해, 연구대상자는 스크리닝, 2 차 방문 시 제공되는 채변키트에 1 g 이상의 분변을 담아 냉동보관한다. 보관된 검체는 운송상자에 동봉하여 외부위탁기관에 이송한 후 분석을 수행한다. 채취된 분변은 분석 후 폐기한다.

검사항목	
단쇄지방산	Acetic acid, Propionic acid, Butyric acid

(17) 삶의 질 평가 설문지(EQ-5D; EuroQol-5 Dimensions)

EQ-5D 는 건강관련 삶의 질을 측정하는 도구로 Euro Quality of Life (Euro Qol) Group 에 의해 개발되었으며 현재의 건강상태를 묻는 5 개의 영역인 ① 운동능력(mobility), ② 자기관리(self-care), ③ 일상생활(usual activity), ④ 통증/불편감(pain/discomfort), ⑤ 불안/우울(anxiety/depression)로 구성되어 있다. 각각의 질문에 세 단계 (즉, 수준 1, 전혀 문제없음; 수준 2, 중등도 문제 있음; 수준 3, 심각한 문제 있음) 중에서 현재의 본인의 건강상태를 가장 잘 설명하는 응답을 선택하도록 한다. 5 개 영역 모두 불편이 없으면 EQ-5D index 는 1 이되며, 산정 방법에 따라 산출된 EQ-5D index 는 소수점 셋째 자리까지 기재한다.

EQ-5D index = 1 -(0.050 + 0.096 × M2 + 0.418 × M3 + 0.046 × SC2 + 0.136 × SC3 + 0.051 × UA2 + 0.208 × UA3 + 0.037 × PD2 + 0.151 × PD3 + 0.043 × AD2 + 0.158 × AD3 + 0.050 × N3)

(M: 운동능력, SC: 자기관리, UA: 일상생활, PD: 통증/불편감, AD: 불안/우울)

※ 변수정의

변수명	정의
M2	운동능력이 '수준 2' 인 경우 1; 그렇지 않으면 0
M3	운동능력이 '수준 3' 인 경우 1; 그렇지 않으면 0
SC2	자기관리가 '수준 2' 인 경우 1; 그렇지 않으면 0
SC3	자기관리가 '수준 3' 인 경우 1; 그렇지 않으면 0
UA2	일상활동이 '수준 2' 인 경우 1; 그렇지 않으면 0
UA3	일상활동이 '수준 3' 인 경우 1; 그렇지 않으면 0
PD2	통증/불편감이 '수준 2' 인 경우 1; 그렇지 않으면 0
PD3	통증/불편감이 '수준 3' 인 경우 1; 그렇지 않으면 0
AD2	불안/우울이 '수준 2' 인 경우 1; 그렇지 않으면 0
AD3	불안/우울이 '수준 3' 인 경우 1; 그렇지 않으면 0
N3	'수준 3' 이 하나라도 있을 때 1; 그렇지 않으면 0

<부록 2> 삶의 질 평가 설문지

(18) 진단검사의학 검사

진단검사의학 검사는 12 시간 이상 공복상태에서 채혈하여 측정하는 것을 원칙으로 하되, 시험책임자의 판단에 따라 달리 시행할 수 있으며, 채취된 혈액은 분석 후 폐기한다. 검사 항목은 다음과 같다.

검사항목	
혈액학적 검사	WBC, RBC, Hb, Hct, PLT
혈액생화학적 검사	ALP, gamma-GT, AST, ALT, total bilirubin, total protein, albumin, BUN, creatinine, CK, LD, uric acid

✓ 스크리닝 전 최근 1 개월 이내의 진단검사의학 검사 결과로 대체할 수 있다. 추가로 필요한 진단검사의학 검사항목은 해당 인체적용시험 스크리닝 방문일에 검사한다{단, 유효성 평가항목은 예외임(재검사 필요함)}

(19) 검체분석기관/위탁기관

① 검체분석기관: 원광대학교병원 / (재)발효미생물산업진흥원/ ㈜한국의과학연구원

② 검체분석기관장: 서일영 / 정도연/ 정문규

③ 검체분석기관 주소: 전북 익산시 무왕로 895 / 전라북도 순창군 순창읍 민속마을길 61-28 / 대전 중구 대흥로 28 번길 6

(20) 식이섭취조사

식이섭취조사는 식사기록법에 따르며, 연구대상자는 스크리닝, 2 차 방문 시

식이기록지를 교부 받아, 1 차, 3 차 방문 전 3 일(평일 2 일, 주말 1 일) 동안 섭취한 음식물을 모두 기록하고, 시험자는 식이기록지를 회수하여 식이섭취 분석을 시행한다.

<부록 3> 식이기록지

(21) 신체활동 조사

연구대상자는 1 차, 3 차 방문 시 세계신체활동설문(Global Physical Activity Questionnaire: GPAQ)에 의한 신체활동조사 설문지를 작성한다. GPAQ 는 신체활동의 측정을 위하여 개발된 설문지로서 여가활동, 집안활동, 일과 관련된 활동, 장소 이동 시 활동 등 문항으로 구성되어 있다. 평가항목으로 사용되는 신체활동량(MET; Metabolic Equivalent Task)은 물리적인 활동이나 GPAQ 의 데이터 분석에 사용되는 것으로 사람의 휴식대사율에 대한 작업대사율의 상대적인 비율을 나타내며, MET value 가 높을수록 신체활동량이 많다는 것을 의미한다.

<부록 4> 신체활동조사 설문지

(22) 연구대상자 이상사례 모니터링

이상사례에 대한 정보는 수시로 연구대상자에게 자발적인 보고를 하도록 하며, 그 외에 섭취기간 동안 전화방문과 정기방문 시 시험담당자의 면담 및 문진 등 진료를 통하여 확인하다. 이상사례 조사에는 시작일 및 종료일, 이상사례의 정도 및 결과, 인체적용시험용제품과 관련하여 취해진 조치 및 인체적용시험용제품과의 인과관계, 인체적용시험용제품 이외 의심되는 약제명, 이상사례에 대한 치료여부 및 내용 등을 포함한다.

(23) 선정기준/제외기준 확인 (연구대상자 적합성 평가)

스크리닝 방문 시에 연구대상자 동의여부와 인구학적 조사, 병력 및 약품 투여력 조사, 이학적 검사소견, 기타 검사결과를 종합하여 선정기준/제외기준에 합당하는 연구대상자 선정이 이루어졌는지 평가하여 기록한다.

(24) 무작위 배정 및 인체적용시험용제품 처방

선정기준/제외기준에 합당한 연구대상자를 고추발효물 TCG25-26(유용미생물 고함량 고추장분말)군 또는 고추발효물 TMG25-2(유용미생물 저함량 고추장분말)군 또는 고추발효물 TFG25-2(시판용 고추장분말)군으로 무작위배정한다. 무작위배정은 선정기준/제외기준 평가일인 1 차 방문에 이루어지며, 이때에 최초 섭취가 이루어진다. 이후 섭취시작 4 주, 8 주에 연구대상자가 방문하여 인체적용시험용제품을 처방 및 반납하도록 한다.

(25) 반납제품 회수 및 순응도 확인

반납제품 회수 및 인체적용시험용제품의 섭취 순응도는 2 차 방문, 3 차 방문에 연구대상자가 지참하고 온 인체적용시험용제품의 잔량을 제품관리자 또는 연구자가 점검한다.

섭취하고 남은 잔량은 반드시 약국에 반납하여 수불장부를 기록하여야 한다. 순응도는 100%를 초과할 수 없으며, 초과할 경우 100%로 간주한다. 순응도는 정수로 표기한다.

$$제품\ 순응도(\%) = \frac{섭취한\ 제품수}{섭취해야할\ 제품수} \times 100$$

10.2. 채혈량

(단위: mL)

항목	1 회 채혈량	Screening	1 차 방문	2 차 방문	3 차 방문
진단검사의학 검사 (혈액학적 검사, 혈액생화학적 검사), 지질대사지표	6	6	-	-	6
염증관련지표(CRP, ESR)	5	5	-	-	5
혈당관련지표	3	3	-	-	3
방문별 채혈량	-	14	0	0	14

10.3. 관찰 · 임상검사항목(방문별)

(1) 스크리닝 방문(-4 주)

이 인체적용시험에 참가하도록 선택된 연구대상자는 시험에 대한 설명을 듣고 다음 순서에 따라 평가를 받는다.

① 연구대상자를 시험에 참여시키기 전에 시험 과정을 설명하고, 연구대상자에게 서면 동의서를 받는다.

② 연구대상자는 순서대로 스크리닝 번호를 지정받는다.

③ 연구대상자의 인구학적 정보와 병력(외과적 수술력을 포함한 과거력 및 현병력) 및 약물 투여력을 조사/기록한다.

④ 음주 및 흡연력을 조사한다.

⑤ 신체검진, 활력징후(혈압 및 맥박수), 신체계측, 체성분 분석을 실시한다.

⑥ 쿠퍼만지수(Modified kupperman’s index) 검사를 실시한다.

⑦ 선정/제외기준을 만족시키지 못하는 연구대상자를 대상으로 이후 계획된

검사를 실시하지 않을 수 있다.

⑧ 지질대사지표, 염증관련지표, 혈당관련지표, EQ-5D 검사를 실시한다

⑨ 진단검사의학 검사를 실시한다.

⑩ 장내 균총과 단쇄지방산 검사를 위한 채변 키트를 제공한다.

⑪ 다음 방문일을 지정해준다.

(2) 1 차 방문(0 주)

1 차 방문은 스크리닝 방문일 이후에 이루어지며, 다음의 항목을 평가한다.

① 약물투여력 및 의학적 상태변화를 확인한다.

② 활력징후(혈압 및 맥박수)를 실시한다.

③ 연구대상자 선정/제외기준을 최종평가하고, 무작위배정을 실시한다. 단, 선정/제외기준 최종평가에 통과하지 못한 자는 이후 계획한 검사를 실시하지 않을 수 있다.

④ 식이섭취조사 및 신체활동조사를 실시한다.

⑤ 인체적용시험용제품 섭취방법에 대하여 교육을 하고, 인체적용시험용제품을 처방한다.

⑥ 장내 균총과 단쇄지방산 검사를 위한 채변 키트를 수거한다.

⑦ 다음 방문일을 지정해준다.

(3) 2 차 방문(4 주)

인체적용시험용제품의 최초 섭취 이후 4 주째에 2 차 방문이 이루어지며, 다음의 항목을 평가한다.

① 약물투여력 변화를 확인한다.

② 활력징후(혈압 및 맥박수)를 실시한다.

③ 체중을 측정한다.

④ 연구대상자로부터 남은 인체적용시험용제품을 회수하여 개수를 확인하고 순응도를 평가한다.

⑤ 인체적용시험용제품 섭취방법에 대하여 교육을 하고, 인체적용시험용제품을 처방한다.

⑥ 이상사례 발생유무를 확인한다.

⑦ 장내 균총과 단쇄지방산 검사를 위한 채변 키트를 제공한다.

⑧ 다음 방문일을 지정해 준다.

(4) 3 차 방문(8 주; 시험 종료 방문)

인체적용시험용제품의 최초 섭취 이후 8 주째에 3 차 방문이 이루어지며, 다음의 항목을 평가한다.

① 약물투여력 변화를 확인한다.

② 음주 및 흡연력을 조사한다.

③ 신체검진, 활력징후(혈압 및 맥박수), 신체계측을 실시한다.

④ 체성분 분석, 쿠퍼만지수(Modified kupperman’s index), 지질대사지표, 염증관련지표, 혈당관련지표, EQ-5D 검사를 실시한다.

⑤ 진단검사의학 검사를 실시한다.

⑥ 식이섭취조사 및 신체활동조사를 실시한다.

⑦ 연구대상자로부터 남은 인체적용시험용제품을 회수하여 개수를 확인하고 순응도를 평가한다.

⑧ 이상사례 발생유무를 확인한다.

⑨ 장내 균총과 단쇄지방산 검사를 위한 채변 키트를 수거한다.

⑩ 필요한 경우 연구대상자에게 다음 방문 시기를 지정해 준다.

(5) 추적/추가방문(필요 시)

섭취 종료 혹은 조기종료 방문 시까지 지속되는 이상사례가 있었던 경우 혹은 섭취종료 이후 혹은 예정된 방문외(unscheduled)에 추가적인 추적관찰이 필요하다고 시험책임자 또는 연구대상자에 의해 요청이 있을 경우 재 방문하여 다음의 항목을 평가한다.

① 활력징후를 측정한다.

② 신체검진 검사를 실시한다.

③ 지난 방문 이후로 연구대상자의 병력이나 치료제품에 변화가 있는 경우 또는 병용제품이 있는 경우 증례기록서에 기록한다.

④ 이상사례 발생유무와 내용을 조사한다.

단, 시험자의 판단에 따라 비정상적인 결과에 대한 진단검사의학 검사 등이 시행될 수 있다.

11. 예측 부작용 및 사용상의 주의사항

본 인체적용시험용제품을 섭취하였을 때 소화불량, 속쓰림 등이 나타날 수 있으나, 그 외에 발생하는 특징적인 부작용이 없는 것으로 보고되고 있다. 다만, 인체적용시험용제품을 구성하고 있는 재료에 대해 알레르기(allergy)가 있는 경우 피부증상(피부 가려움, 두드러기, 습진, 피부 발적 등), 위장증상(구역, 구토, 복통과 설사), 호흡기증상(재채기, 콧물, 호흡곤란 등) 등을 보일 수 있다. 심한 경우 아나필락시스 쇼크가 발생할 수도 있다. 이와 같은 알레르기를 예방하기 위하여 스크리닝 시 알레르기가 있는 자원자는 인체적용시험 참여가 금지된다. 또한 인체적용시험 진행 중 알레르기가 발병할 경우 해당 연구대상자는 즉시 중단하고 인체적용시험에서 탈락된다.

12. 인체적용시험의 중지 및 탈락기준

☞ 기관생명윤리위원회 (IRB)는 중대한 이상사례가 나타난 경우에는 IRB 검토에 의해 시험을 중지시키거나 인체적용시험 승인을 취소할 수 있다

12.1. 인체적용시험 종료

인체적용시험은 시험에 참여한 연구대상자의 마지막 방문으로 완료되었다고 본다. 시험을 조기 중단한 연구대상자는 시험을 종료한 것으로 보지 않는다. 그러나, 시험 종료 후에도 임상적으로 의미 있는 비정상이 남아 있을 때에는 그 비정상이 해소되거나 임상적으로 안정된 목표점에 도달할 때까지 연구자가 추적 관찰해야 한다.

12.2. 인체적용시험 중지 및 탈락기준

시험에 참여하는 모든 연구대상자의 시험 완료여부를 기록하고, 제품 섭취나 관찰이 중단된 경우에는 해당 이유를 기록한다. 해당 연구대상자의 시험을 중단할 수 있는 사유는 다음과 같다:

- 선정/제외기준을 위반한 경우
- 연구대상자에게 중대한 이상사례 (SAE)이 발생한 경우 또는 이상사례 (AE)으로 인해 연구대상자가 시험중단을 요구하는 경우
- 섭취 전 검사에서 발견하지 못한 전신질환이 발견된 연구대상자
- 연구대상자 또는 법정대리인이 시험 참가동의를 철회하는 경우
- 연구자나 연구대상자에 의해 중대한 시험계획을 위반하는 경우
- 연구대상자의 추적이 안 되는 경우
- 연구대상자에게 인체적용시험용제품을 섭취하는데 문제가 있는 경우
- 인체적용시험용제품 섭취기간 또는 경과 관찰 기간 동안 연구자의 지시 없이 연구결과 또는 판정에 영향을 미칠 수 있는 약물 (제품)등을 복용한 경우
- 기타 시험책임자/담당자의 판단에 의해 연구 진행이 적합하지 못하다고 판단되는 경우

연구대상자가 시험을 완료하기 전에 중단한다면, 인체적용시험 증례기록서와 근거문서에 중단의 이유를 기록해야 한다. 연구대상자에게 배정되었던 시험 제품은 다른 연구대상자에게 배정될 수 없다.

13. 효과 평가기준, 평가방법 및 해석방법(통계 분석 방법)

본 인체적용시험의 연구대상자로부터 얻어진 자료는 크게 Safety 군, ITT(Intention To Treat)군, FA(Full Analysis)군과 PP(Per Protocol)군으로 분석된다.

- Safety 군은 인체적용시험에 참여하여 최소한 1 회 이상 인체적용시험용제품을 섭취한 연구대상자를 대상으로 분석한다.
- ITT 군 분석은 연구대상자 번호를 부여받은 연구대상자를 대상으로 한다.
- FA 군 분석은 무작위배정되어 인체적용시험용제품을 섭취 후 최소 일회 이상의 유효성 평가 결과가 있는 연구대상자를 대상으로 한다. 아래의 연구대상자는 FA 분석에서 제외하며, 특정 결론에 영향을 미칠 가능성이 있는 계획서 위반을 포함한다. 사소한 위반은 이에 해당하지 않는다.
  - 선정/제외기준 위반
  - 인체적용시험용제품을 한번도 섭취하지 않은 자
  - 무작위배정 이후 자료(유효성 또는 안전성 평가 결과값)가 전무한 자
- PP 군 분석은 FA 군에 포함되는 연구대상자 중 중대한 위반 없이 인체적용시험계획서에 따라 인체적용시험을 완료한 연구대상자를 대상으로 한다. 아래와 같은 위반사항은 PP 군 분석에서 제외하게 된다.
  - 병용금지약물의 복용
  - 기타 중대한 인체적용시험계획서 위반사항이 발생한 경우 등

  이 외 중대한 계획서 위반 여부는 분석군이 확정되기 전에 Data Review Meeting 을 통해 결정한다.
- 유효성 평가에 대한 자료는 PP 군을 주 분석대상으로 하되, ITT 군 및 FA 군을 추가적으로 분석하여 유효성을 평가할 수 있다. 안전성 평가에 대한 자료는 Safety 군에 대해 분석하여 본 인체적용시험용제품에 대한 안전성을 평가한다.
- 분석에 사용할 프로그램은 Window 용 SPSS 또는 SAS 를 이용하고, 통계학적 유의수준은 p 값이 0.05 미만으로 설정한다.
- 결측치는 LOCF(Last Observation Carried Forward)에 의하여, 결측치 발생 전 마지막 관측치로 대치한다.
- 집단 간 기저치 분포의 차이가 있는지를 확인하기 위한 검정은 Chi-Square test 또는 Fisher’s exact test 와 Independent t-test 를 이용한다.

13.1. 유효성평가

1 차 유효성 평가 변수(장내 균총 변화, 쿠퍼만지수) 및 2 차 유효성 평가 변수(체성분 분석, 신체계측지표, 지질대사지표, 염증관련지표, 혈당관련지표, 단쇄지방산, EQ-5D) 결과에 대해서 섭취군 별로 기술통계량을 제시하고, 섭취군 간 비교와 섭취군 내 비교를 실시한다. 섭취군 간 비교의 경우, 정규성 검정을 수행하여 정규성을 만족하였을 때, 변화량(섭취 8 주 후-섭취 전)에 대해서 independent t-test 를 이용하고,

만족하지 못하였을 때 Mann-Whitney U test 를 이용하여 분석한다. 고추발효물 TCG25-26(유용미생물 고함량 고추장분말)군과 고추발효물 TFG25-2(시판용 고추장분말)군, 고추발효물 TMG25-2(유용미생물 저함량 고추장분말)군과 고추발효물 TFG25-2(시판용 고추장분말)군, 고추발효물 TCG25-26(유용미생물 고함량 고추장분말)군과 고추발효물 TMG25-2(유용미생물 저함량 고추장분말)군 간 변화량(섭취 8 주 후-섭취 전)에 대해서 independent t test 또는 Mann-Whitney U test 를 이용하여 분석한다.

또한 고추발효물 TCG25-26(유용미생물 고함량 고추장분말)군, 고추발효물 TMG25-2(유용미생물 저함량 고추장분말)군, 고추발효물 TFG25-2(시판용 고추장분말)군 간 차이에 대해서 정규성 만족하였을 때 Linear mixed model 을 이용하고, 만족하지 못하였을 때 Friedman’s test 를 이용하여 분석한다.

섭취군 내 비교의 경우, 정규성 검정을 수행하여 정규성을 만족하였을 때, 섭취 전 대비 8 주 시점의 변화에 대하여 paired t test 를 이용하고, 만족하지 못하였을 때 Wilcoxon signed-rank test 를 이용하여 분석한다. 각 유효성 평가 항목간 상관 관계를 비교하고자 Pearson correlation test 를 실시할 수 있다.

체중의 반복측정에 대해서 섭취군 간 비교와 섭취군 내 비교를 RM-ANOVA/Linear mixed model 또는 Friedman’s test 를 이용하여 분석한다. 섭취군 간 차이가 있는 항목에 대하여 사후검정(post-hoc analysis)을 실시한다. 장내 균총 변화 결과에 대해서 반응자/비반응자를 분류하여 분석할 수 있으며, chi-square test 또는 Fisher’s exact test 를 이용하여 분석할 수 있다.

단, 정규성이 심각하게 위배되는 경우 로그변환방법(logarithm transformation) 등의 변수변환을 실시한 후 검정하거나 비모수검정법인 Wilcoxon signed-rank test, Mann-whitney test 등을 실시할 수 있다.

연속형 자료의 경우 분포에서 비정상적으로 벗어난 유효성 평가지표에 대해 이상치(±1.5SD 이상)로 정의하고, 분석에서 제외할 수 있다.

생활습관(식이섭취, 신체활동, 음주, 흡연 등) 및 동질하지 않은 각 유효성 평가 항목의 기저치 및 인구학적정보 항목은 covariate 로 보정하여 ANCOVA, ranked ANOVA 를 실시할 수 있다. 유효성 평가에 영향을 미칠 것으로 판단되는 항목(BMI, 쿠퍼만지수 등)에 대하여 층화분석(sub group-analysis)을 실시할 수 있다. 잠재적 혼동요인(나이, 식이섭취, 신체활동, 음주, 흡연, 염증지표, 이상사례, 병용약물, 병력, 이상치 등)에 따라 추가분석을 실시할 수 있다.

13.2. 안전성분석

(1) 이상사례

인체적용시험 기간 동안 발생한 모든 이상사례에 대하여 전체와 섭취군 별로 빈도, 퍼센트로 요약한다. 섭취군 간 이상사례의 발생 양상을 chi-square test 또는 Fisher’s exact test 를 이용하여 분석한다.

(2) 진단검사의학 검사, 활력징후

진단검사의학 검사와 활력징후 결과에 대하여 섭취군 별로 기술통계량을 제시하고, 섭취군 간 비교와 섭취군 내 비교를 실시한다. 섭취군 간 비교의 경우, 변화량(섭취 8 주 후-섭취 전)에 대하여 independent t-test 를 이용하여 분석한다. 섭취군 내 비교의 경우, 섭취 전 대비 8 주 시점의 변화에 대하여 paired t test 를 이용하여 분석한다. 또한, 활력징후의 반복측정에 대해서 각 섭취군 간 비교와 섭취군 내 비교를 RM-ANOVA/Linear mixed model 등을 적용하여 분석한다. 단, 정규성검정을 통하여 정규성 만족을 하지 못할 경우, 비모수적 분석방법을 이용한다 (각 섭취군 내 비모수적 분석방법: Wilcoxon signed-rank test, 섭취군 간 비모수적 분석방법: Mann-Whitney U test).

14. 이상사례

14.1. 이상사례(Adverse Event, AE)

이상사례는 인체적용시험용제품을 섭취한 연구대상자에게 일어나는 모든 원하지 않은 의학적 사건이다. 이상사례는 반드시 인체적용시험용제품과 인과관계를 필요로 하지는 않는다. 따라서 이상사례는 인체적용시험용제품과의 관련여부와 상관없이 인체적용시험용제품의 섭취와 일시적으로 관련된 바람직하지 못하고 의도되지 않은 모든 징후(비정상적인 결과 포함), 증상 또는 질환이 될 수 있다. 여기에는 새로 발생하거나 또는 기초평가일 상태와 비교하여 발현 정도 또는 발현 횟수가 악화되는 것, 실험실적 검사이상을 포함한 비정상적 진단결과의 관찰도 포함된다.

14.2. 약물이상반응(Adverse Drug Reaction, ADR)

약물이상반응은 인체적용시험용제품의 임의 용량에서 발생한 모든 유해하고 의도되지 않은 반응으로서 인체적용시험용제품과의 인과관계를 부정할 수 없는 경우를 말한다.

14.3. 중대한 이상사례(Serious Adverse Event, SAE)

ICH/KGCP 에 의해 내려진 중대한 이상사례의 정의는 다음과 같다:

- 사망하거나 생명에 대한 위험이 발생한 경우
- 입원할 필요가 있거나 입원 기간을 연장할 필요가 있는 경우
- 영구적이거나 중대한 장애 및 기능 저하를 가져온 경우
- 태아에게 기형 또는 이상이 발생한 경우
- 위의 사례 외에 약물의존성이나 남용의 발생 또는 혈액질환 등 그 밖에 의학적으로 중요한 상황이 발생하는 사례

위에 열거된 상황 이외의 경우에도 신속한 보고가 필요한지 여부를 판단하기 위해 의학적·과학적 판단을 해야 한다. 예를 들어, 즉각적으로 연구대상자의 생명을 위협하지 않으며 사망 또는 입원을 유발하지는 않으나, 연구대상자의 상태를 악화시키고 또는 위의 정의에서 명시된

상황을 예방하기 위한 개입이 요구될 수 있다. 어떠한 이상사례가 상당히 유의한 임상적 영향을 갖는 임상징후나 증상과 연관되는 것으로 시험책임자에 의해 판단되는 경우 중대한 이상사례로 여겨진다. 단, 증상 악화와 관련이 없는 다음과 같은 사유로 인한 입원에 대해서는 중대한 이상사례로 간주되지 않는다.

- 진단 또는 기존에 가지고 있던 질환에 대해 선택적인 수술을 받기 위한 입원 또는 입원기간의 연장(선택적인 수술을 받는 동안 발생한 예상하지 못한 이상사례는 중대한 이상사례의 기준에 맞으면 보고되어야 한다.)
- 임상시험 전 예정된 치료를 위한 입원이나 입원기간의 연장(ex: 기존질환, 건강검진 등)
- 입원은 병원에서 24 시간 이상 머문 경우에 해당하며, 입원하지 않더라도 중대한 것으로 분류될 수 있는 다른 사유가 있을 수 있다(ex: 흉통 등)

이상사례의 정도

1 = 경증 (Mild)	연구대상자가 거의 느끼지 못할 정도로 정상적인 일상생활 (기능)을 저해하지 않는 정도. 대부분 치료가 필요하지 않는 정도
2 = 중등증 (Moderate)	연구대상자가 불편감을 느낄 수 있으며, 정상적인 일상생활 (기능)을 저해하는 정도. 연구대상자가 시험을 계속할 수는 있으나 치료가 필요할 수도 있는 정도
3 = 중증 (Severe)	연구대상자가 매우 불편하여 일상생활 (기능)이 불가능하고, 시험의 계속적인 참여가 불가능한 정도. 치료나 입원이 필요할 수 있는 정도

인체적용시험용제품과의 인과관계

인과관계	판단근거
명확히 관련이 있음 (Definitely related)	- 인체적용시험용제품을 섭취하였다는 증거가 있는 경우<br>- 인체적용시험용제품 섭취가 다른 어떤 이유보다 가장 개연성 있게 설명되는 경우<br>- 섭취 중단으로 이상사례가 사라지는 경우<br>- 재섭취(Rechallenge, 가능한 경우에만 실시) 결과가양성인경우<br>- 이상사례가 인체적용시험용제품 또는 동일계열의 이 식품에 대해 이미 알려져 있는 정보와 일관된 양상을 보이는 경우
관련이 있다고 생각됨 (Probably related)	- 인체적용시험용제품을 섭취하였다는 증거가 있는 경우<br>- 인체적용시험용제품 섭취와 이상사례 발현의 시간적 순서가 타당한 경우<br>- 이상사례가 다른 원인보다 이 인체적용시험용제품 섭취에 의해 더욱 개연성 있게 설명되는 경우<br>- 섭취중단으로 이상사례가 사라지는 경우
관련이 있을 가능성이 있음 (Possibly related)	- 인체적용시험용제품을 섭취하였다는 증거가 있는 경우<br>- 인체적용시험용제품 섭취와 이상사례 발현의 시간적 순서가 타당한 경우<br>- 이상사례가 다른 가능성 있는 원인들과 같은 수준으로 본제에 기인한다고 판단되는 경우<br>- 섭취중단으로(실시된 경우) 이상사례가 사라지는 경우
관련이 없다고 생각됨 (Probably not related)	- 인체적용시험용제품을 섭취하였다는 증거가 없는 경우<br>- 이상사례에 대해 보다 가능성 있는 다른 원인이 있는 경우<br>- 섭취중단결과(실시된 경우)가 음성이거나 모호한 경우
명확히 관련이 없다고 생각됨 (Definitely not related)	- 인체적용시험용제품을 섭취하였다는 증거가 없는 경우<br>- 이상사례에 대해 가장 개연성 있게 설명되는 다른 어떤 이유가 있는 경우<br>- 섭취중단결과(실시된 경우)으로 이상사례가 소실되지 않는 경우
불명 (Unknown)	- 정보가 불충분하거나 상충되어 판단할 수 없고 이를 보완하거나 확인할 수 없는 경우

이상사례와 관련하여 취해진 조치

0 = 취해진 조치 없음<br>(No action taken)
1 = 인체적용시험용제품의 일시적 섭취 중단<br>(Study product temporarily interrupted)
2 = 인체적용시험용제품의 섭취 중단<br>(Study product permanently discontinued)
3 = 치료약물 병용투여<br>(Concomitant medication taken)
4 = 비약물치료<br>(Non-drug therapy given)
5 = 입원/입원기간의 연장<br>(Hospitalization/prolonged hospitalization)

이상사례 결과

1 = 완전치유(후유증 없음)
2 = 치유(후유증 있음)
3 = 진행 중
4 = 영구적 손상
5 = 사망

14.4. 시험책임자/담당자에 의한 이상사례의 평가 및 보고

- 시험책임자는 시험 시작일로부터 시험 종료 시점까지 발생한 이상사례를 보고한다. 중대한 이상사례의 기준을 만족시키는 이상사례는 "중대한 이상사례 보고서 양식"을 사용하여 보고한다. 의뢰자는 인체적용시험계획서에 제시된 기간을 경과한 후 연구자가 보고하는 안전성 정보에 대해서도 평가한다.

- 이상사례 기록: 이상사례의 심각성, 발현 정도 또는 섭취제품과의 연관성과 관계없이 모든 이상사례는 근거문서와 증례기록서에 의학적 용어로 기록되어야 한다. 징후 및 증상이 일반적인 병인에서 기인할 때 가능한 경우 이에 대한 진단이 실시되어야 한다. 연구자는 증례기록서에 이상사례와 인체적용시험용제품 섭취 사이의 관련성에 관한 소견을 기록해야 한다. 이상사례 관리를 위해 요구되는 모든 측정 방법들은 의뢰자의 지시사항에 따라 근거문서에 기록되어야 한다.

- 인체적용시험의뢰자의 책무: 의뢰자는 명시되지 않은 제품 섭취와 관련된

모든 중대한 이상사례를 시험책임자에게 보고하여야 한다.

- 시험책임자/담당자의 책무: 시험 중 발생한 모든 중대한 이상사례는 시험책임자/담당자에 의해서 인지된 후 24 시간 이내에 해당 의뢰자의 연락 담당자에게 보고되어야 한다. 중대한 이상사례는 중대한 이상사례 보고서 양식을 통해 의뢰자 (필요한 경우 IRB)에게 전달되어야 하며, 이는 연구담당자에 의해 서명되어야 한다. 중대한 이상사례의 첫 보고는 이메일, 팩스나 전화로 가능하다. 중대한 이상사례는 이메일로 보고하는 것이 권장된다. 전화 보고 이후 중대한 이상사례에 대한 보고는 구두보고 후 72 시간 이내 (근무일 기준)에 연구담당자에 의해서 중대한 이상사례 보고서 양식을 작성/완료하여 이메일로 전송되어야 한다.

- 시험 종료 시까지 해결되지 않거나, 연구대상자의 연구참여 중단으로 해결되지 않은 모든 중대한 이상사례는 다음과 같이 될 때까지 추적조사를 실시해야 한다:
    - 이상사례의 회복
    - 이상사례의 안정화
    - 기초 평가일 상태로 회복
    - 인체적용시험용제품 또는 인체적용시험 실시 이외의 다른 사항에서 기인한 것으로 판단되는 경우
    - 추가적 정보를 얻을 수 없는 가능성이 높을 경우

<u>모든 이상사례 보고 연락처</u>
의뢰자: (재)발효미생물산업진흥원<br>담당자: 기능성평가팀 팀장 양희종<br>전화 : 063-650-2034<br>팩스 : 063-650-9590<br>HP : 010-8845-0789<br>Email : godfiltss@naver.com

시험책임자 : 한아름 교수

전화 : 063-859-2543

HP : 010-8648-7371

Email : qibosarang@naver.com

15. 인체적용시험 후 연구대상자의 진료 및 치료기준

이상사례로 인해 인체적용시험에서 탈락된 연구대상자는 다른 적절한 치료를 받을 수 있도록 지도하며, 시험이 종료된 연구대상자에서 기대하지 않았던 지연 이상사례가 발생한 경우 정당한 진료를 한다.

16. 연구대상자의 안전 보호에 관한 대책

16.1. 인체적용시험 실시기관

실시기관의 장은 해당 인체적용시험의 실시에 필요한 공간 및 설비와 전문인력을 갖추고, 해당 시험을 적정하게 실시할 수 있도록 준비에 완벽을 기해야 한다.

16.2. 시험책임자

시험책임자는 시험이 인체적용시험계획서, GCP/KGCP 에 대한 현 ICH 지침 및 기타 해당 허가 요구사항에 부합하도록 보장할 책임이 있다. GCP/KGCP 는 연구대상자의 시험 참여에 관련된 연구의 기획, 실행, 기록 및 보고에 대한 국제적 윤리 및 과학적 질의 기준이다. 이 기준의 준수는 연구대상자의 권리, 안전 및 복지에 대한 공식적인 보장, 헬싱키 선언에서 비롯된 원칙의 준수, 시험 자료의 신뢰를 의미한다.

16.3. 기관생명윤리위원회 (IRB)

인체적용시험의 승인을 얻거나 승인 받은 시험을 변경하여 실시하고자 하는 경우, 인체적용시험계획서 또는 변경 계획서에 대하여 IRB 의 승인을 받는다. 승인 이전에 연구대상자를 시험에 참여 시킬 수 없다.

시험을 시작하기 전에 시험책임자나 의뢰자는 IRB 에 다음 문서들의 최근 완성된 사본을 제출한다:

- 최종 인체적용시험계획서 및 수정사항
- 인체적용시험 의뢰자가 승인한 연구대상자 동의서와 연구대상자에게 제공되는 기타 모든 문서
- 인체적용시험 자료집 또는 해당 정보
- 인체적용시험 의뢰자가 동의한 연구대상자 모집 공고문
- 인체적용시험 관련 상해에 대한 보상 및 연구대상자에 대한 지불 정보
- 연구지원금, 의뢰자의 이름, 연관기관 및 연구대상자 사례비에 관련된 정보
- IRB 가 책임을 완수하기 위해 요구하는 기타 모든 문서

본 시험은 IRB 가 최종 인체적용시험계획서, 수정사항, 연구대상자 동의서, 해당되는 연구대상자 모집 공고문 및 연구대상자 보상 프로그램에 대한 완전한 허가를 내리고, 의뢰자가 이 허가의 사본을 받은 후에야 시작될 수 있다. 허가서는 날짜를 명시하고 허가된 문서들을 정확히 명시해야 한다. 그러나, 시험 도중 시험책임자 또는 의뢰자는 다음 문서들을 해당될 경우 IRB 에 제출해 검토와 허가를 받는다:

- 인체적용시험계획서의 변경사항
- 연구대상자 동의서와 기타 서면 문서들의 변경사항
- 인체적용시험 의뢰자가 허용한 새로운 또는 변경된 연구대상자 모집 방법
- 중대하거나, 예상되지 않았거나, 인체적용시험용제품과 관련된 이상사례의 보고
- 연구대상자의 안전이나 시험의 실행에 영향을 줄 수 있는 새로운 정보
- 연구대상자의 즉각적인 피해를 제거하기 위한 인체적용시험계획서의 변화
- 시험책임자가 변경될 경우
- IRB 의 기타 모든 요구사항

인체적용시험의 종료 시, 시험책임자는 IRB 에 시험의 완료를 공지해야 한다.

16.4. 인체적용시험 의뢰자와 응급연락

응급상황 발생시 시험책임자 및 담당자들은 인체적용시험계획서에 기재되어 있는 전화번호로 의뢰자에게 연락해야 한다.

16.5. 응급상황 시 인체적용시험용제품 확인 (이중눈가림 해제)

본 인체적용시험에서는 시험책임자 (또는 인체적용시험용제품 제품관리자)와 의뢰자가 무작위배정 코드를 각각 보관하도록 한다. 일반적인 경우 모든 연구대상자들이 시험을 완료하고 자료의 입력이 완료되기 전에는 이중눈가림을 해제해서는 안된다. 다만, 연구대상자의 섭취상황을 알아야 하는 응급치료의 경우에는 시험책임자의 판단 하에 이상사례가 발생한 연구대상자 개인으로 제한하여 이중눈가림을 해제할 수 있다. 이 경우 시험책임자는 의뢰자에게 가능한 한 24 시간 이내에

신속하게 연락해야 한다. 이중눈가림을 해제하게 된 날짜, 시간, 이유를 증례기록서와 자료문서의 적절한 곳에 문서화해야 한다. 또한 이중눈가림이 해제된 봉인 봉투는 의뢰자에게 보내 재봉인을 하여 보관하여야 한다. 의뢰자로부터 받은 이중눈가림 코드 해제 확인 서류 복사본은 근거문서에 보관되어야 한다.

16.6. 응급 치료

연구대상자의 시험 참여 도중 및 이후, 시험책임자와 시험기관은 시험과 관련된 임상적으로 중요한 연구대상자의 이상사례에 대하여 적절한 치료가 제공되도록 해야 한다. 중대한 이상사례 또는 예상하지 못한 이상사례가 발생할 경우 적절한 치료가 제공될 수 있도록 모든 연구대상자는 보험에 가입하게 된다. 따라서 시험책임자 및 시험기관은 연구대상자가 시험기간 중 의학적 조치가 필요할 때 연구대상자에게 이를 알려야 한다.

16.7. 이상사례 발생시 조치

이상사례 발생시 즉시 필요한 검사 및 치료를 받을 수 있도록 관리한다. 중대한 이상사례 발생시에는 시험을 중지하고 이상사례 보고방법 및 응급절차에 따라 신속하고 적절한 조치를 취한다.

17. 그 밖에 인체적용시험을 안전하게 과학적으로 실시하기 위하여 필요한 사항

17.1. 인체적용시험계획서의 숙지

본 시험은 Helsinki 선언에 입각하여 연구대상자의 권리와 복지를 염두에 두고 준비된 것으로서, 시험책임자 및 담당자들이 연구계획을 정확히 분석 및 숙지하고, 연구대상자의 문제점을 적극적으로 대응한다.

17.2. 연구대상자 모집 방법

연구대상자 모집은 별첨된 연구대상자 모집 문건을 인체적용시험 실시기관내・외 공고 및 홈페이지 게시를 통하여 모집한다. 연구대상자 모집 문건을 통해 취약한 환경의 연구대상자(원내 직원)가 모집될 수 있으나, 연구책임자 및 연구자와 직접적으로 관련이 있는 경우는 배제하여 진행할 예정이며, 그 외의 경우는 '17.3. 인체적용시험의 동의', '17.4. 정확한 연구대상자의 선정'에서 명시된 동의 및 선정 절차에 따라 본 연구에 참여를 결정하게 된다.

17.3. 인체적용시험의 동의

연구대상자의 동의는 대상자가 연구에 참가하기 전에 독립된 장소에서 시행하며 시험책임자 및 위임받은 자가 별첨된 설명문과 동의서에 따라 대상자에게 인체적용시험에 대한 시험내용 및 인체적용시험용제품의 효과, 이상사례 및 안전성에 대한 모든 사항을 설명한 후 자발적으로 본 연구에 참여하겠다는 서면 동의를 받는다. 설명으로부터 동의를 결정하기까지의 시간은 충분히 주도록 한다.

17.4. 정확한 연구대상자의 선정

본 인체적용시험에 앞서 연구대상자와의 충분한 면담 및 검사를 통하여 연구대상자 적합여부에 대하여 철저히 평가한다.

17.5. 인체적용시험의 진행 점검

시험책임자는 의뢰자에게 주기적으로 이상사례, 시험진행 상황, 결과 등에 대하여 보고하며, 의뢰자는 주기적으로 시험 진행 상황에 대하여 점검을 실시한다.

17.6. 인체적용시험 실시기관의 모니터링

연구대상자의 권리와 복지 보호, 보고된 시험 관련 자료가 근거문서와 대조하여 정확하고, 완전하며, 검증이 가능한지 여부 확인, 승인된 인체적용시험계획서, ICH 및 GCP 에서 정한 지침에 따라 모든 증례기록서와 근거문서, 그리고 적용법령이 요구가 수행되는지의 여부 확인을 위하여 모니터링을 실시한다.

인체적용시험에 대한 모니터링은 의뢰자의 담당 모니터가 정기적인 시험기관 방문과 전화를 통해서 이루어 질 것이다. 방문 시 모니터는 기본적으로 연구대상자기록 원본, 제품 관리 기록, 자료 보관(연구 파일) 등을 확인한다. 또한, 모니터는 시험진행과정을 잘 살피고, 문제가 있을 경우 연구자와 상의한다.

이들 방문의 적절한 시간은 연구자와 모니터가 협의하여 배분하여야 한다. 연구자는 또한 GCP/KGCP 에 정의된 것과 같이, 모니터가 증례기록서에 기입된 자료들을 확인할 수 있는 연구대상자의 원 자료들 (source documents : 병원 또는 개인 차트, 실험실 결과 기록, 예약 기록 등)을 볼 수 있도록 해주어야 한다.

17.7. 연구대상자의 비밀유지

연구대상자의 신원을 파악할 수 있는 기록은 비밀로 보장될 것이며, 시험의 결과가 출판될 경우에도 연구대상자의 신원을 비밀상태로 유지한다. 구체적인 내용은 다음과 같다:

본 시험에 관련된 의뢰자, 모니터 및 점검자는 본 시험의 모니터링과 점검 및 진행사항 관리를 위한 목적으로 연구대상자의 기록을 열람할 수 있다. 연구자는 본 시험의 계약이 체결됨으로 의뢰자 또는 모니터 및 점검자가 연구대상자의 차트와 증례기록서 기록을 검증하기 위하여 해당 문서를 검토하거나 복사할 수도 있음을 숙지하여야 한다. 이러한 정보들은 기밀로 보관되어야 하며, 기밀 보관을 위한 시설과 그 관리기준을 갖추고 있어야 한다. 한편, 증례기록서 등 시험에 관련된 모든 서류에는 연구대상자 이름이 아닌 연구대상자식별코드(일반적으로 연구대상자의 이니셜)로 기록하고 구분한다.

17.8. 연구대상자를 위한 보험가입

본 인체적용시험에서는 연구대상자의 이상사례 발생시 적절한 치료가 제공될 수 있도록 시험에 참여하는 모든 연구대상자를 대상으로 보험에 가입한다.

17.9. 인체적용시험 자료 및 기록보관

ICH 및 GCP/KGCP 에서 정한 지침에 따라 시험책임자/기관은 모든 인체적용시험 문서뿐만 아니라, 각 연구대상자로부터 수집한 자료를 보충하는 모든 증례기록서와 근거문서, 그리고 적용법령이 요구하는 특정 문서들을 유지보관 하여야 한다. 기본문서 및 기타 자료는 이중장금장치가 있는 곳에서 보관하며, 연구 종료 후 3 년간 보관하며, 임상개발 중단 시 공식적인 중단일로부터 3 년간 보관하며, 의뢰자 요청 시 합의 하에 최대 3 년간 연장 보관이 가능하다. 시험책임자/기관은 불의의 또는 조기의 문서유실을 막기 위한 조치를 취해야 한다. 만약, 자료보관의 책임을 맡은 시험책임자가 은퇴하거나 재배정 되는 경우 또는 기타의 이유로 기록의 보관에 대한 책임을 지지 않게 될 경우, 자료의 보관 업무는 이를 수행할 다른 사람에게 인계되어야 한다. 의뢰자는 새로운 자료 관리자의 성명 및 소재지에 관한 통보문을 받아야 한다. 의뢰자에게 서면으로 승인을 받기 이전에는 어떤 경우에도 시험책임자는 연구문서를 이전하거나 처리할 수 없다. 만일 시험에 관련된 문서들을 의뢰자가 검토해야 한다면 시험책임자는 이를 허용하여야 한다.

18. 참고문헌

1) http://kosis.kr. 국가통계포털. 2022년 우리나라 년도별 중위연령. 2024.04.30 (검색).

2) Van Keep P. A. & Kellerhals J. M. (1976). The ageing woman. About the influence of some social and cultural factors on the changes in attitude and behaviour that occur during and after the menopause. Acta obstetricia et gynecologica Scandinavica, 51, 19-27.

3) The Society of Korean Medicine Obstetrics and Gynecology. Oriental Obstetrics &Gynecology (下). 1st rev. ed. Seoul: Euiseongdang. 2012:265-76, 408-9, 418, 432-4.

4) Morishige K, Morishige K, Matsumoto K, Ohmichi M, Nishio Y, Adachi K et al. Clinical features affecting the results of estrogen replacement therapy on bone density in Japanese postmenopausal women. Gynecol Obstet Invest. 2001;52(4):223-6.

5) Cauley JA, Zmuda JM, Ensrud KE, Bauer DC, Ettinger B et al. Timing of estrogen replacement therapy for optimal osteoporosis prevention. J Clin Endocrinol Metab. 2001;86:5700-5.

6) Chung YJ, Kim MR, Jeong HW, Yoon BK, Lee BS, Kang BM et al. Changing Korean Menopausal Women's Awareness on Hormone Therapy: 7-years after Women's Health Initiative Study. J Korean Soc Menopause. 2012;18(2):94-9.

7) Rossouw JE, Anderson GL, Prentice RL, LaCroix AZ, Kooperberg C, Stefanick ML et al. Risks and benefits of estrogen plus progestin in healthy postmenopausal women: principal results From the Women's Health Initiative randomized controlled trial. JAMA. 2002;288:321-33.

8) Chlebowski RT, Hendrix SL, Langer RD, Stefanick ML, Margery G, Dorothy L et al. WHI Investigators. Influence of estrogen plus progestin on breast cancer and mammography in healthy postmenopausal women. the women's health Initiative randomized trial. JAMA. 2003;289:3243-53

9) Shin, D., Hong, S. B., Geum, J. H., Ma, J. Y., & Chung, H.-S. (2016, September 30). Effects of Schisandrae Fructus on menopause symptoms in ovariectomized mice. Journal of Korean Medicine. The Society of Korean Medicine. https://doi.org/10.13048/jkm.16033

10) 이희삼(Heui Sam Lee), 전호정(Ho Jung Jeon), 이상덕(Sang Duk Lee), 문재유(Jae Yu Moon), 김애정(Ae-Jung Kim), and 류강선(Kang Sun Ryu). "식이 뽕잎이 흰쥐의 장내균총 조성에 미치는 영향." 한국식품과학회지 33.2 (2001): 252-255.

11) 장소희, 김효진, 하창우, 최정훈, 김성혁, 이태성, 손은화. (2023). 청양고추의 항산화 및 미백효과. 한국산학기술학회 논문지, 24(6), 377-385, 10.5762/KAIS.2023.24.6.377

12) 김재용 (Jae Yong Kim),박경욱 (Kyung Wook Park),양현숙 (Hyun Sook Yang),조영숙 (Young Sook Cho),정창호 (Chang Ho Jeong),심기환 (Ki Hwan Shim),이성태 (Sung Tae

Yee),and 서권일 (Kwon Il Seo). "양파고추장 메탄올추출물의 항암 및 면역활성." 한국식품저장유통학회지 12.2 (2005): 173-178.

13) Mahoro, Patience et al. “Protective Effect of Gochujang on Inflammation in a DSS-Induced Colitis Rat Model.” Foods (Basel, Switzerland) vol. 10,5 1072. 12 May. 2021

14) Costa JPL, Brito HO, Galvão-Moreira LV, Brito LGO, Costa-Paiva L, Brito LMO. Randomized double-blind placebo-controlled trial of the effect of Morus nigra L. (black mulberry) leaf powder on symptoms and quality of life among climacteric women. Int J Gynaecol Obstet. 2020 Feb;148(2):243-252. doi: 10.1002/ijgo.13057. Epub 2019 Dec 11. PMID: 31736077.

15) 옥선명, and 김경수. "폐경 후 및 폐경 주변기 여성의 폐경 증상과 척추 골밀도의 연관성." Korean Journal of Family Medicine 18.8 (1997): 844-854.

16) Lim EY, Lee SY, Shin HS, Lee J, Nam YD, Lee DO, Lee JY, Yeon SH, Son RH, Park CL, Heo YH, Kim YT. The Effect of Lactobacillus acidophilus YT1 (MENOLACTO) on Improving Menopausal Symptoms: A Randomized, Double-Blinded, Placebo-Controlled Clinical Trial. J Clin Med. 2020 Jul 9;9(7):2173. doi: 10.3390/jcm9072173. PMID: 32660010; PMCID: PMC7408745.

17) Rural Development Administration. (2015), 백수오 등 복합추출물의 여성갱년기 증상개선 효능에 대한 다기관인체임상연구. https://doi.org/10.23000/TRKO201500010535

<부록 1> 쿠퍼만 지수(Modified Kupperman' s index, KI)

스크리닝 번호	1 - S	1 - R	
방 문	☐ 스크리닝 방문	☐ 3 차 방문	
작성일	20 / /	연구대상자 서명	

쿠퍼만 지수(Modified Kupperman' s index, KI)

갱년기 증상	건강상태정도				가중치	점수
	0(없음)	1(약간)	2(보통)	3(심함)		
1. 안면홍조(얼굴이 화끈거림)	0	1	2	3	4	
2. 감각이상(손발저림)	0	1	2	3	2	
3. 불면증(잠을 못이룸)	0	1	2	3	2	
4. 신경질(특별한 이유없이 화가 남)	0	1	2	3	2	
5. 우울증	0	1	2	3	1	
6. 현기증(어지럼증)	0	1	2	3	1	
7. 피로감(쉽게 피곤함을 느낌)	0	1	2	3	1	
8. 두통(머리아픔)	0	1	2	3	1	
9. 관절통/근육통	0	1	2	3	1	
10. 가슴 두근거림	0	1	2	3	1	
11. 의주감(개미가 기어가는 듯한 감각)	0	1	2	3	1	
갱년기 증상 점수						점
질 건조감/질 분비물 감소	0	1	2	3	1	점

**점수 = 증상 정도 ✕ 가중치

(예) 안면홍조 증상이 심할 경우 증상 정도는 3 이며, 가중치는 4 이므로 안면홍조의 점수는 12 점(3✕4)으로 채점함.

<부록 2> 삶의 질 평가 설문지(EQ-5D; EuroQol-5 Dimensions)

스크리닝 번호	1 - S		1 - R	
방 문	☐ 스크리닝 방문		☐ 3 차 방문	
작성일	20 / /	연구대상자 서명		

삶의 질 평가 설문지<br>(EQ 5D: EuroQol-5 Dimensions)		
• 아래의 각 문항에서, 오늘 당신의 건강상태를 가장 잘 설명해주는 하나의 항목에 표시해 주십시오.		
차원	항목	척도
운동능력	나는 걷는데 지장이 없다	☐
	나는 걷는데 다소 지장이 있다	☐
	나는 종일 누워있어야 한다	☐
자기관리	나는 목욕을 하거나 옷을 입는데 지장이 없다	☐
	나는 혼자 목욕을 하거나 옷을 입는데 다소 지장이 있다	☐
	나는 혼자 목욕을 하거나 옷을 입을 수가 없다	☐
일상활동	나는 일상활동을 하는데 지장이 없다	☐
	나는 일상활동을 하는데 다소 지장이 있다	☐
	나는 일상활동을 할 수가 없다	☐
통증/불편	나는 통증이나 불편감이 없다	☐
	나는 다소 통증이나 불편감이 있다	☐
	나는 매우 심한 통증이나 불편감이 있다	☐
불안/우울	나는 불안하거나 우울하지 않다	☐
	나는 다소 불안하거나 우울하다	☐
	나는 매우 심하게 불안하거나 우울하다	☐
총점		

<부록 3> 식이섭취조사

식이기록지

방 문	☐ 1차 방문 ☐ 3차 방문
연구대상자 번호	1 - R
연구대상자 이니셜	

연구대상자 확인	본 식이기록지에 기록된 내용은 모두 본인이 기재 및 수정하였음을 확인합니다.
	날짜: 년 월 일 (서명)

* 식이기록지는 방문 전 3일(평일 2일, 주말 1일)을 작성해주세요

원광대학교병원

[식이기록지 - 예시]

· 하루 동안에 먹은 음식을 모두 기록해 주세요.
· **볼펜**으로만 작성해주세요(샤프, 연필, 잉크펜 사용금지).
· 오기 시 잘못 기록된 부분은 한줄로 긋고 다시 기록해주세요(수정액, 수정테이프사용 금지).
· 하루 동안 섭취한 음료(차류, 우유, 커피, 쥬스 등 포함)을 모두 기록해주세요.
· 섭취한 음식의 재료명을 정확히 기입해주세요.
  (예 – 제육볶음에서 양파를 빼고 먹었다면, 양파는 기록하지 말 것.)
· 샐러드, 샤브샤브, 견과류 등은 음식에 들어간 재료명에 따른 각각의 먹은 양을 기입해주세요.
  (예 – 샐러드를 먹었을 경우 → 양상추 5 젓가락, 양배추 4 젓가락, 방울토마토 2 개, 키위드레싱 2 숟가락)
· 떡, 부침개, 김 등 크기가 분명하지 않은 음식들은 크기를 그림란에 정확히 기입해주세요.
  (예 – 손가락 한마디정도가 2 cm)

식사	식사 시간	식사 장소	음식명 (조리명)	재료명	눈대중	그림
식전	7:10	집	보리차		2 종이컵	
아침 식사	9:30	집	식빵<br>흰우유<br>딸기잼	서울우유	1 장<br>200 ml<br>1/2 숟가락	
간식	11:00	학교	자판기커피	밀크커피	1 종이컵	
점심 식사	1:00	학교 식당	비빔밥<br><br>풋고추멸치조림<br><br>배추김치	백미<br>시금치나물<br>콩나물무침<br>고추장<br>당근<br>풋고추, 멸치<br>간장	1 대접<br>(또는 드신 그릇의 양을 나타내주세요)<br><br>3 젓가락<br>(또는 ~큰술)<br>5 쪽	
간식	4:30	학교 매점	칸쵸(과자)<br>포카리스웨트		1 각<br>1 병(500 ml)	
저녁 식사	7:00	집	콩밥<br>돼지고기김치찌개<br><br>고등어조림<br>풋고추<br>된장<br>조미김	백미, 검은콩<br>돼지고기, 김치, 양파, 두부<br><br>고등어, 무	1/2 공기<br>1 대접<br><br>1 토막, 1 토막<br>1 개<br>1/2 티스푼<br>8 장	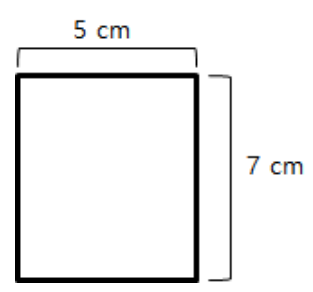
간식	9:00	집	사과<br>콩떡	검은콩, 쌀	1/2 개<br>1 개	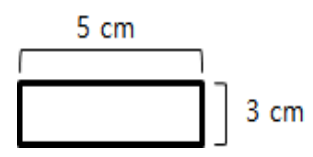

날짜:　　　년　　월　　일　　　　　　　　연구대상자 번호: 1-R

[식이기록지 - 1 일]

- 밥은 공기기준, 국은 대접기준으로 작성하여 주시기 바랍니다(예-1 공기, 1/2 대접).

식사	식사 시간	식사 장소	음식명 (조리명)	재료명	눈대중	그림
식전						
아침 식사						
간식						
점심 식사						
간식						
저녁 식사						
간식						

날짜: 년 월 일 연구대상자 번호: 1-R

[식이기록지 - 2 일]

- 밥은 공기기준, 국은 대접기준으로 작성하여 주시기 바랍니다(예-1 공기, $^1/_2$ 대접).

식사	식사 시간	식사 장소	음식명 (조리명)	재료명	눈대중	그림
식전						
아침 식사						
간식						
점심 식사						
간식						
저녁 식사						
간식						

날짜: 년 월 일 연구대상자 번호: 1-R

[식이기록지 - 3 일]

- 밥은 공기기준, 국은 대접기준으로 작성하여 주시기 바랍니다(예-1 공기, 1/2 대접).

식사	식사 시간	식사 장소	음식명 (조리명)	재료명	눈대중	그림
식전						
아침 식사						
간식						
점심 식사						
간식						
저녁 식사						
간식						

<부록 4> 신체활동조사

연구대상자 번호	1 - R		
방 문	☐ 1 차 방문		☐ 3 차 방문
작성일	20 / /	연구대상자 서명	

세계신체활동설문(GPAQ)

▣ 직업 및 가사 활동

본 설문은 급료를 받거나 받지 않는 일, 가사 활동, 농업/어업 활동, 음식 마련위한 각종 활동 및 구직 활동에 관한 것입니다

1. **숨이 많이 차거나 심박동이 많이 증가하는 매우 힘든 일**을 10분 이상 지속적으로 하십니까?
(매우 힘든일 : 무거운 물건을 나르거나 들기, 땅파기 또는 건설 현장 일 등)
☐ 예 ☐ 아니오(4번 문항으로 가시오)

2. 그런 매우 힘든 일을 일주일에 보통 며칠하십니까?
☐ 1일 ☐ 2일 ☐ 3일 ☐ 4일 ☐ 5일 ☐ 6일 ☐ 7일(매일)

3. 그런 매우 힘든 일을 하루에 보통 얼마나 하십니까?
☐☐ 시간 ☐☐ 분

4. **숨이 조금 차거나 심박동이 조금 증가하는 힘든 일**을 10분 이상 지속적으로 하십니까?
(힘든 일 :빠르게 걷기, 가벼운 물건 나르기 등)
☐ 예 ☐ 아니오(7번 문항으로 가시오)

5. 그런 힘든 일을 일주일에 보통 며칠하십니까?
☐ 1일 ☐ 2일 ☐ 3일 ☐ 4일 ☐ 5일 ☐ 6일 ☐ 7일(매일)

6. 그런 힘든 일을 하루에 보통 얼마나 하십니까?
☐☐ 시간 ☐☐ 분

▣ 일상에서 장소 이동을 위한 걷기, 자전거 타기

* 이미 언급한 직장(일터)및 가정에서의 신체활동은 제외 하십시오.
본 설문은 일상에서 단순히 장소 이동을 위한 활동을 묻는 것으로, 예를 들면, 출퇴근을 위해, 쇼핑하기 위해, 시장 가기 위해, 종교 시설(교회 등)을 가기 위한 활동에 관한 것입니다

7. 장소 이동을 위해 최소 10분 이상 걷거나 자전거(페달용)를 탑니까?
☐ 예 ☐ 아니오(10번 문항으로 가시오)

8. 장소 이동을 위한 걷기와 자전거(페달용)를 타는 날은 일주일에 보통 며칠입니까?
☐ 1일 ☐ 2일 ☐ 3일 ☐ 4일 ☐ 5일 ☐ 6일 ☐ 7일(매일)

9. 장소 이동을 위해 걷기와 자전거(페달용) 타기는 하루에 보통 얼마나 하십니까?
☐☐ 시간 ☐☐ 분

▣ 여가 시간 활동

* 이미 언급한 직장(일터)및 가정에서의 신체활동 그리고 일상에서의 걷기나 자전거 타기는 제외 하십시오
본 설문은 여가 시간에 하는 스포츠, 체력 단련 운동 및 레크리에이션 활동에 관한 것입니다

10. **숨이 많이 차거나 심박동이 많이 증가하는 매우 힘든 활동**을 10분 이상 지속적으로 하십니까? [매우 힘든 활동 : 줄넘기,등산, 에어로빅, 스포츠 댄스, 조깅, 달리기, 배드민턴(경기용), (빠른)수영, 축구, 농구, 스쿼시, 단식 테니스, 빠른 속도로 자전거 타기, 등]
☐ 예 ☐ 아니오(13번 문항으로 가시오)

11. 그런 매우 힘든 활동을 일주일에 보통 며칠하십니까?
☐ 1일 ☐ 2일 ☐ 3일 ☐ 4일 ☐ 5일 ☐ 6일 ☐ 7일(매일)

12. 그런 매우 힘든 활동을 하루에 보통 얼마나 하십니까?
☐☐ 시간 ☐☐ 분

13. 숨이 **조금 차거나 심박동이 조금 증가하는 힘든 활동**을 10분 이상 지속적으로 하십니까? [힘든 활동 : 빠르게 걷기, 생활체조(댄스 등), 에어로빅, 배드민턴(비경기용), 탁구, 볼링, (느리게)자전거 타기, (느린) 수영, 골프, 배구 등)]
☐ 예 ☐ 아니오(16번 문항으로 가시오)

14. 그런 힘든 활동을 일주일에 보통 며칠하십니까?
☐ 1일 ☐ 2일 ☐ 3일 ☐ 4일 ☐ 5일 ☐ 6일 ☐ 7일(매일)

15. 그런 힘든 활동을 하루에 보통 얼마나 하십니까?
☐☐ 시간 ☐☐ 분

▣ 좌업 생활

본 설문은 직장(일터) 및 가정에서 그리고 특정 장소로 오고 갈 때, 앉아있거나 기댄 상태로 보낸 시간을 묻는 것으로, 잠자는 시간은 제외 하십시오
예) 책상에 앉아 책 읽기, 친구와 얘기 나누기, 카드 게임을 하는 경우 그리고 승용차, 버스, 기차 여행 시 그리고 TV 시청을 위해 앉아서 보낸 경우 등

16. 앉아있거나 기댄 상태로 보낸 시간은 하루에 보통 얼마입니까?
☐☐ 시간 ☐☐ 분

<부록 5> 헬싱키 선언

헬싱키 선언

- 인간을 대상으로 하는 의학연구에 있어서의 윤리 원칙 -

A. 서문

1. 세계의사회는 헬싱키 선언을 통하여 개인을 식별할 수 있는 인체유래물이나 정보를 이용하는 연구를 포함한 인간 대상 의학연구의 윤리원칙을 제안하고 발전시켰다. 이 선언은 전문을 포괄적으로 이해하여야 하며 각 조항은 관련된 다른 조항을 고려하여 적용하여야 한다.
2. 세계의사회에 위임된 권한에 따라 이 선언은 의사에 게 우선적으로 적용한다. 세계의사회는 인간 대상 의학연구에 관련된 다른 연구자들도 이 원칙을 준수하도록 권장한다.

B. 일반 원칙

3. 세계의사회 은 "환자의 건강은 의사의 최 우선 직무이여야 한다,"로 의사의 의무를 촉구하며, 은 "의사는 진료에 임할 때 환자의 최 선의 이익에 입각하여 진료하여야 한다."고 선언한다.
4. 의사의 의무는 의학연구와 관련된 사람을 포함하여 환 자의 건강, 안녕 및 권리를 증진시키고 보호하는 것이다. 의 사는 지식과 양심에 따라 이 의무를 다하여야 한다.
5. 의학의 발전은 궁극적으로 인간 대상 의학연구를 포함하는 연구에 기반을 둔다.
6. 인간 대상 의학연구의 근본적인 목적은 질병의 원인, 발 생 과정, 경과를 이해하고, 예방법, 진단절차, 치료법을 향상시키는 데 있다. 입증된 최선의 시술일지라도 안전성, 효과, 효능, 접근성, 질에 대한 지속적인 연구로 재평가하여야 한다.
7. 의학연구는 모든 연구대상자에 대한 존중을 함양하고 보장하며 그들의 건강과 권리를 보호하는 윤리기준을 따라 야 한다.
8. 의학연구의 근본적인 목적이 새로운 지식의 창출이지 만 이러한 목적이 결코 연구대상자 개인의 권리와 이익보다 우선할 수는 없다.
9. 의학연구를 수행하는 의사는 연구대상자의 생명, 건강, 존엄, 완전성, 자기결정권, 사생활 및 개인정보의 비밀을 보호할 의무가 있다. 연구대상자 보호의 책임은 항상 의사 또는 다른 의료전문가들에게 있으며 비록 그들이 동의를 하였다고 하더라도 결코 그 책임이 연구대상자에게 있 지 않다.
10. 의사는 인간 대상 연구에 있어서 적용 가능한 국제적 규범과 기준뿐 아니라 자국의 윤리적, 법적, 제도적 규범과 기준을 동시에 고려해야 한다. 어떤 국가적 또는 국제적 차 원의 윤리적, 법적, 규제 요건도 이 선언에서 제시하는 연구대상자에 대한 보호조치를 축소하거나 없애서는 안 된다.

11. 의학연구는 환경에 미칠 수 있는 위해를 최소화하는 방법으로 수행하여야 한다.
12. 인간 대상 의학연구는 적절한 윤리적·과학적 교육과 훈련을 받은 자격이 있는 사람이 수행하여야 한다. 환자나 건강한 자원자에 대한 연구는 적절한 자격을 갖추고 숙련된 의사나 관련 의료전문가가 감독하여야 한다.
13. 의학연구에서 그 대표성이 부족할 수 있는 집단에게 연구에 참여할 수 있는 적절한 기회가 제공하여야 한다.
14. 의학연구와 진료를 겸하는 의사는 잠재적 예방, 진단 및 치료의 가치에 의해 연구가 정당화될 수 있는 범위에서 만, 그리고 연구 참여가 연구대상자가 되는 환자의 건강에 부정적으로 영향을 미치지 않을 것이라는 믿을 만한 충분 한 근거를 가지고 있는 경우 자신의 환자를 연구에 참여하 게 해야 한다.
15. 연구에 참여한 결과로서 해를 입은 연구대상자에게 적 절한 배상과 치료를 보장해야 한다.

C. 위험, 부담 및 이익

16. 의료행위와 의학연구에서 대부분의 의학적 중재행위는 위험과 부담을 수반한다. 인간 대상 의학연구는 연구목적의 중요성이 연구대상자의 위험과 부담보다 더 중대할 경우에 한하여 수행할 수 있다.
17. 모든 인간 대상 의학연구의 수행에 앞서, 연구 대상이 되는 개인과 집단에게 예견할 수 있는 위험 및 부담은 이들 과 더불어 그 연구로 영향을 받는 다른 개인이나 집단에게 예측되는 이득과 비교하여 신중한 평가를 선행해야 한다. 위험을 최소화할 수 있는 조치를 하여야 한다. 연구자는 위험을 지속적으로 감시하고 평가하여 기록하여야 한다.
18. 위험을 적절하게 평가하였고 충분히 관리할 수 있다는 확신이 없다면, 의사는 인간 대상 연구에 참여해서는 안 된다. 위험이 잠재적 이익을 초과하는 것으로 밝혀지거나 확 정적 결과에 대한 결정적 증거가 있을 때, 의사는 그 연구를 계속할지, 변경할지 또는 즉각 중단할지를 평가를 해야 만한다.

D. 취약한 집단 및 개인

19. 일부 집단과 개인은 특별히 취약하여 부당한 취급을 받 거나 추가적인 해를 입을 가능성이 높을 수 있다. 모든 취약 한 집단과 개인은 특성에 맞게 배려된 보호를 받아야 한다.
20. 취약한 집단과 함께하는 의학연구는 오직 이 집단의 건강상의 요구나 우선순위에 부합하는 연구이고, 취약하지 않은 집단에서는 수행할 수 없는 연구일 때 정당화된다. 더 불어, 이 집단은 해당 연구결과로 얻은 지식, 의료행위, 또는 시술의 혜택을 받을 수 있어야 한다.

E. 과학적 요건 및 연구계획서

21. 인간 대상 의학연구는 일반적으로 인정되는 과학적 원 칙을 따라야 하고, 과학문헌에 대한 면밀한 지식, 그 밖에 관련된 정보 출처, 적절한 실험, 그리고, 필요할 경우, 동물실험에 기초해야 한다. 연구에 이용된 동물의 복지는 존중되어야 한다.
22. 인간을 대상으로 하는 각각의 연구에 대한 설계와 수행은 계획서에 명확하게 기술하고 그 정당성을 확보하여야 한다. 연구계획서는 관련된 윤리적 고려사항을 명시하여야 하며, 본 선언의 원칙이 어떻게 반영되었는지 기술하여야 한다. 연구계획서는 재원, 의뢰자, 소속 기관, 잠재적 이해상충, 연구대상자 보상 및 이 연구에 참여한 결과로 인해 해를 입었을 경우 연구대상자에 대한 치료 그리고/또는 배상 규정에 관한 정보를 포함하고 있어야 한다. 임상시험의 경우 임상시험 종료 후 적절한 사후관리에 관한 적절한 계획도 기술하여야 한다.

F. *연구윤리위원회*

23. 연구를 시작하기 전에 연구계획서를 관련된 연구윤리 위원회에 심의, 조언, 지도, 승인을 위하여 제출하여야 한다. 위원회는 운영에 있어서 투명하여야 하며, 연구자, 의뢰자 그리고 기타 부당한 영향으로부터 독립적이어야 하며, 적법한 절차에 따라 자격을 갖추어야 한다. 위원회는 관련된 국제 규범과 기준뿐 아니라, 연구가 수행되는 국가의 법률과 규정을 고려하여야 한다. 그러나 본 선언에서 제시한 연구대상자를 위한 어떤 보호기준도 축소하거나 없앨 수 없다. 위원회는 수행 중인 연구를 감시할 권리를 가져야 한다. 연구자는 위원회에 감시 정보, 특히 중대한 유해사례에 대한 정보를 제공해야 한다. 연구계획서는 위원회의 심의와 승인이 없이는 변경할 수 없다. 연구가 종료된 후 연구자는 연구결과와 결론의 요약이 포함된 최종보고서를 위원회에 제출해야 한다.

G. *사생활과 비밀유지*

24. 연구대상자의 사생활을 보호하고 개인정보의 비밀유지를 위해 모든 주의를 기울여야 한다.

H. *충분한 설명에 의한 동의*

25. 의학연구 대상자로서 충분한 설명에 의한 동의를 할 수 있는 사람의 참여는 자발적이어야 한다. 연구자는 가족이나 지역사회의 지도자와 의논할 수는 있지만, 충분한 설명에 의한 동의가 가능한 사람이 자유롭게 동의한 것이 아니라면 연구에 등록하여서는 안 된다.
26. 충분한 설명에 의한 동의를 할 수 있는 사람이 관련된 의학연구에서, 각 잠재적인 연구대상자에게 각 연구의 목적, 방법, 재원의 출처, 가능한 모든 이해상충, 연구자의 소속기관, 연구에서 예견되는 이익과 잠재적 위험, 연 구에 수반되는 불편, 연구 종료 후 지원, 그리고 기타 연구에 관련된 측면들에 대해 충분하게 설명하여야 한다. 잠재적인 연구대상자에게 어떠한 불이익 없이

연구참여를 거절할 수 있는 권리와, 참여에 대한 동의를 언제든지 철회할 수 있는 권리가 있다는 것을 충분히 설명하여야 한다. 또한, 정보의 전달 방법뿐만 아니라 잠재적인 개별 연구대상자의 특정 정보 요구에도 특별한 주의를 기울여야 한다. 의사 또는 다른 적절한 자격을 갖춘 사람은 잠재적인 연구대상자가 정보를 이해한 것을 확인한 후에 잠재적인 연구 대상자의 자발적인 충분한 설명에 의한 동의를 구해야 하 며, 동의는 가급적이면 서면으로 받는다. 만약 동의를 서 면으로 할 수없는 경우라면, 증인의 입회하에 그 사실을 문서로 작성해 두어야 한다. 모든 의학연구의 대상자에게는 연구의 일반적 성과와 결과에 대한 설명을 받을 선택권 이 주어져야 한다.

27. 의사는 연구 참여에 대해 충분한 설명에 의한 동의를 구할 때, 잠재적인 연구대상자가 의사에게 의존적 관계에 있 는지 또는 강압에 의해 동의하는지 특히 주의하여야 한다. 그런 상황에서라면 그 관계에서 완전히 독립적인 적절한 자 격의 사람이 충분한 설명에 의한 동의를 구해야 한다.

28. 충분한 설명에 의한 동의를 할 능력이 없는 잠재적인 연구대상자인 경우, 의사는 합법적인 대리인에게 충분한 설 명에 의한 동의를 구해야 한다. 충분한 설명에 의한 동의를 할 능력이 없는 잠재적인 연구대상자들이 속한 집단의 건강 증진을 목적으로 하고, 동의가 가능한 사람으로 대체해서 수 행할 수 없고, 최소 위험과 최소 부담만을 수반하는 연구를 제외하고는 이들은 자신들에게 이익의 가능성이 없는 연구에 포함되어서는 안 된다.

29. 충분한 설명에 의한 동의를 할 능력이 없다고 여겨지는 잠재적인 연구대상자가 연구 참여에 대한 결정에 찬성할 수 있다면, 의사는 합법적인 대리인의 동의와 함께 본인의 찬성을 구해야 한다. 잠재적인 연구대상자의 반대의사를 존중하여야한다.

30. 예를 들어 의식이 없는 환자와 같이, 신체적 또는 정 신적으로 동의능력이 없는 연구대상자를 포함하는 연구는 충분한 설명에 의한 동의를 못하게 하는 신체적 또는 정신 적 상태가 연구 대상 집단의 필수적인 특성인 경우에만 수행될 수 있다. 그런 경우 의사는 합법적인 대리인으로부터 충 분한 설명에 의한 동의를 구해야 한다. 법정대리인과 접촉할 수 없지만 연구를 지체해선 안 된다면, 동의를 할 수 없는 상 태에 있는 피험자를 대상으로 하는 구체적인 이유가 연구계 획서에 명시되어 있고 연구윤리위원회가 그 연구를 승인한 경우에만 동의 없이도 연구를 수행할 수 있다. 단, 연구에 계 속 참여할지에 대한 동의는 대상자나 그의 합법적인 대리인 으로부터 가능한 한 빨리 받아야 한다. 31. 의사는 환자에게 진료의 어떤 측면이 연구와 관계되는 지 충분히 알려주어야 한다. 환자가 연구 참여를 거부하거나 참여를 철회하여도 환자-의사 관계에 부정적인 영향을 미쳐 서는 안 된다.

32. 인체유래물은행이나 유사한 저장소에 보관된 인체유 래물이나 정보에 관한 연구처럼 개인을 식별할 수 있는 인체 유래물이나 정보를 이용하는 의학연구의 경우, 의사는 그 수 집, 보관 및 재사용에 관하여 충분한 설명에 의한 동의를 구해야 한다. 동의를 얻는 것이 불가능하거나 비현실적인 예외적 상황은 있을 수 있다. 그런 경우는 연구윤리위원회의 심의와 승인을 받은 경우에만 수행하여야 한다.

I. 위약의 사용

33. 새로운 시술의 이익, 위험, 부담 및 효과는 입증된 최 선의 시술의 이익과 위험, 부담, 효과와 비교하여 검증해야 한다. 다음의 경우는 제외한다. 입증된 시술이 없는 경우, 위약을 사용하거나 시술을 하지 않는 것을 허용할 수 있다; 또는 설득력 있고 과학적으로 타당한 방법론적 이유로 입증된 최선의 시술보다 효과가 낮은 시술을 사용하거나, 위약을 사용하거나 시술을 하지 않는 것이 시술의 효능과 안전 성을 결정하는데 필요하며, 입증된 최선의 시술보다 효과가 낮은 시술을 받거나, 위약을 받거나, 시술을 받지 않는 환 자라도 입증된 최선의 시술을 받지 않은 결과로 중대하거나 돌이킬 수 없는 위해를 입을 추가적인 위험이 없는 경우 이 러한 선택의 남용을 방지하기 위하여 최대한의 주의를 기울 여야 한다.

J. 임상시험 후 지원

34. 의뢰자와 연구자 및 연구시행 국가의 정부는 임상시 험에서 이로운 것으로 확인된 시술이 여전히 필요한 참여 자들을 위하여 이들이 임상시험 종료 후에도 이용할 수 있는 지원책을 임상시험에 앞서 마련하여야 한다. 이러한 정 보는 충분한 설명에 의한 동의 과정에서 참여자에게 공개하 여야 한다.

K. 연구등록 및 결과의 출간 및 배포

35. 모든 인간 대상 연구는 최초 연구대상자를 모집하기 전에 일반대중이 접근할 수 있는 데이터베이스에 등록하여야 한다.

36. 연구자, 저자, 의뢰자, 편집인 및 발행인은 모두 연구 결과의 출간 및 배포에 관한 윤리적 책무를 진다. 연구자는 인간 대상 연구의 결과를 일반대중에게 공개할 의무가 있으 며, 보고의 완성도와 정확성에 책임을 진다. 모든 당사자들 은 윤리적인 보고에 대한 인정된 지침을 준수하여야 한다. 긍정적 결과뿐 아니라 부정적이고 확정되지 않은 결과도 출 판하거나 다른 방법으로 대중에게 공개하여야 한다. 출판물 에는 재원 출처, 소속기관 및 이해상충에 대해 밝혀야 한다. 이 선언의 원칙을 따르지 않은 연구의 보고서는 출간하도록 허용해서는 안 된다.

L. 임상 실무에서 입증되지 않은 시술

37. 입증된 시술이 없거나 다른 시술이 있지만 효과적이지 않은 환자치료의 경우, 의사의 판단에 의해 그 시술이 환자의 생명을 구하거나 건강을 회복시키거나 또는 고통을 경감할 희망이 있다면, 의사는 전문가의 조언을 구한 후, 환자 또는 합법적인 대리인의 충분한 설명에 의한 동의를 얻어 입증되지 않은 시술방법을 사용할 수 있다. 이 시술은 이후에 그 안전성과 유효성 평가를 위해 고안된 연구의 대상이 되어야 한다. 어떠한 경우라도 항상 새로운 정보는 기록하여야 하고 적절하게 공개하여야 한다
