# Supplementary material for: Effects of gochujang formulations on menopausal symptoms and gut microbiota in postmenopausal women: an 8-week randomized, double-blind trial
Source: Front Nutr. 2026 Sep 10;13:1849944. doi: 10.3389/fnut.2026.1849944 (PMC13600830; doi:10.3389/fnut.2026.1849944)
Supplement: Supplementary file 2 [file Data_Sheet_2.PDF]

Healthcare Claims Co., Ltd.

506-1, 1115 Jeongnyeorip-ro, Deokjin-gu, Jeonju-si, Jeonbuk-do, 54858, Republic of Korea
Tel. 063-213-8285 / Fax. 063-276-8287 / Representative: Gi-chan Ha

Document No.: HC-2026-0127-01
Date: January 27, 2026
To: Wonkwang University Hospital
Cc: Professor Han A-Lum, Wonkwang University Hospital

Subject: Clarification Regarding IRB Approval and CRIS Registration

1. Our institution served as a contract research organization (CRO) responsible for the operational support of this clinical trial, and worked in collaboration with the principal investigator and the sponsoring institution to support study conduct based on IRB approval and the approved study protocol.

2. This study was conducted after obtaining formal approval from the Institutional Review Board(IRB) on April 28, 2025, and all study procedures, including participant recruitment, were performed diligently in accordance with the approved protocol (Appendix. Certification of IRB Review).

3. Registration in the Clinical Research Information Service (CRIS) was completed on October 30, 2025, which represents a delay in administrative registration following the initiation of the study. In the CRIS system, submission of the IRB approval letter is mandatory at the time of registration, and the IRB approval date and approved study details are automatically reflected in the system. Accordingly, CRIS registration records, including IRB approval information and any protocol amendment approvals, cannot be arbitrarily modified or altered by the investigators due to the system's structural safeguards.

4. In addition, the entry of the number of study participants as 45 during CRIS registration was a clerical input error. The study was designed and conducted based on the IRB-approved sample size of 60 participants, as clearly stated in

the IRB approval letter. Participant recruitment, data collection, and all analyses were performed strictly in accordance with the IRB-approved protocol.

Upon identification of this discrepancy, the CRIS registration record was corrected to reflect the approved sample size of 60 participants. This administrative input error and subsequent correction did not affect the ethical conduct of the study or the scientific validity and reliability of the study results.

In summary:

- The study was initiated after IRB approval;
- The study was conducted in full compliance with the approved protocol;
- The delayed CRIS registration and the incorrect entry of the sample size were administrative and clerical issues only and did not affect the timing, ethical integrity, or scientific reliability of the study.

Accordingly, we respectfully request that the delayed CRIS registration and the corrected clerical error not be interpreted as evidence of inappropriate study conduct, as this study was performed in full compliance with IRB requirements and established ethical standards for clinical research.

Appendix. Certification of IRB Review

Healthcare Claims Co., Ltd.

Certification of IRB Review

You can varify the document by using the QR code.

※ Relevant documents will be retained for 3 years in accordance with institution policy						
**To**	**Sponsor**	Ministry of Agriculture, Food and Rural Affairs				
	**Principal Investigator**	A Lum Han / WONKWANG UNIVERSITY HOSPITAL				
**File No. (The number received from the IRB)**		WKUH IRB 2025-03-030-002	**Submission Type**	Response to Contingent Approval	**Notification Date**	2025.04.30
**Study Title**		Korean	고추발효물(고추장분말)의 장내 균총 및 갱년기 여성건강에 대한 유효성 및 안전성을 평가하기 위한 8 주, 무작위배정, 이중눈가림 인체적용시험			
		English	A 8 weeks, randomized, double-blind human trial to evaluate the efficacy and safety of Fermented hot pepper(Gochujang powder)on intestinal microflora and menopausal women's health			
**Study Nick Name**						

**Research classification**	Other Research		
**Number of Subjects**	Total	60 persons	
	Korea	60 persons	
	Institution	60 persons	
**Approval period**	2025-04-28 ~ 2026-04-27		
**Sponsor**	Ministry of Agriculture, Food and Rural Affairs		
**List of submission documents**	1. 보험 증권<br>2. 위원회 검토 답변서<br>3. 변경대비표		
**Approval date**	2025.04.28	**Review type**	Expedited Review
**Determination**	● Approved ○ Approved with conditions		
**Contents of review**	The Institutional Review Board (IRB) has reviewed your research study titled “A 8 weeks, randomized, double-blind human trial to evaluate the efficacy and safety of Fermented hot pepper(Gochujang powder)on intestinal microflora and menopausal women's health“ under<br>Expedited Review and has determined that it is approved as of 2025.04.28.<br>This content is not included in the basic translation service. An additional fee is required.<br><br>This protocol was resubmitted following the previous expedited review decision as approval with revision.<br>The revised protocol has been approved for one year of research (number of participant: 60).<br>Please use the informed consent form which was approved (perforated) by the IRB. Furthermore,<br>if you wish to continue the research beyond the approval period, you must submit an interim report<br>at least two months prior to the approval expiry date (April 27, 2026), i.e. by February 27, 2026.		

The above document is verified and provided by Ethical People Co., Ltd. at the request of the R-Bay user. We hereby certify that the translation of the above document is true, accurate, and complete to the best of our knowledge.

Please contact us at ethical@r-bay.co.kr

Ethical People Co., LTD CEO 이 선우 Lee

통지서

QR코드를 읽어 문서 진위여부를 확인할 수 있습니다.

※ 본 과제의 문서보존기간은 3년입니다.

수신	지원(의뢰)기관	농림축산식품부			
	연구책임자	한아름 / 원광대학교병원 / 가정의학과			
File No.		WKUH IRB 2025-03-030-002	신청유형	시정보고서	통지일자 2025.04.30
과제명	국문	고추발효물(고추장분말)의 장내 균총 및 갱년기 여성건강에 대한 유효성 및 안전성을 평가하기 위한 8 주, 무작위배정, 이중눈가림 인체적용시험			
	영문	A 8 weeks, randomized, double-blind human trial to evaluate the efficacy and safety of Fermented hot pepper(Gochujang powder)on intestinal microflora and menopausal women's health			
Study Nick Name					

연구구분	임상시험 외 연구			
대상자 수	전체	60명		
	국내	60명		
	본원	60명		
연구승인기간	2025-04-28 ~ 2026-04-27			
지원(의뢰) 기관	농림축산식품부			
제출서류 목록	1. 보험 증권<br>2. 위원회 검토 답변서<br>3. 변경대비표			
심사일	2025.04.28		심사종류	신속심사
중간보고 시기	2026년 02월 27일까지		비고	
심사결과	● 승인 ○ 시정승인			
내용	본 연구과제는 기심사 시 시정승인 과제로 수정하여 제출한 바 1년간 연구의 진행을 승인합니다. 그러므로 연구대상자의 동의서는 IRB에서 천공을 받아 동의 취득을 하시기 바랍니다. 그리고 승인 유효기간 이후에도 임상연구를 지속하기 위해서는 적어도 승인 만료(2026.04.27), 2개월 전(2026.02.27)까지 임상연구의 진행상황에 대하여 중간보고 의뢰를 하셔야 함을 안내드립니다.			
별첨				

원광대학교병원기관생명윤리위원회
